# Impaired fracture healing is associated with callus chondro-osseous junction abnormalities in periostin-null and osteopontin-null mice

**DOI:** 10.3389/ebm.2024.10066

**Published:** 2025-01-22

**Authors:** Marc Teitelbaum, Maya D. Culbertson, Charlene Wetterstrand, J. Patrick O’Connor

**Affiliations:** ^1^ Department of Orthopaedics, Rutgers-New Jersey Medical School, Newark, NJ, United States; ^2^ Rutgers-School of Graduate Studies, Newark Health Sciences Campus, Newark, NJ, United States

**Keywords:** periostin, osteopontin, endochondral ossification, chondro-osseous junction, fracture healing, osteoclast, cyclooxygenase-2

## Abstract

Periostin and osteopontin are matricellular proteins abundantly expressed in bone fracture callus. Null mutation of either the periostin (*Postn*) gene or the osteopontin (*Spp1*) gene can impair bone fracture healing. However, the cell and molecular pathways affected by loss of POSTN or SPP1 which lead to impaired fracture healing are not well understood. To identify potential pathways, a detailed radiological, histological, and immunohistochemical analysis of femur fracture healing in *Postn*-null (*Postn*KO), *Spp1*-null (*Spp1*KO), and normal (WT) mice was performed. Apparent changes in specific protein levels identified by immunohistochemistry were confirmed by mRNA quantitation. Comparisons between the *Postn*KO and *Spp1*KO fracture calluses were confounded by interactions between the two genes; loss of *Postn* reduced *Spp1* expression and loss of *Spp1* reduced *Postn* expression. Consequently, alterations in fracture healing between mice heterozygous for the *Postn*-null allele (*Postn*HET) as well as the *Postn*KO and *Spp1*KO mice were similar. Calluses from *Postn*HET, *Postn*KO, and *Spp1*KO mice all had dysmorphic chondro-osseous junctions and reduced numbers of osteoclasts. The dysmorphic chondro-osseous junctions in the *Postn*HET, *Postn*KO, and *Spp1*KO calluses were associated with reduced numbers of MMP-13 expressing hypertrophic chondrocytes, consistent with delayed cartilage resolution. Unlike collagen X expressing callus chondrocytes, chondrocytes only expressed MMP-13 when localized to the chondro-osseous junction or after traversing the chondro-osseous junction. Cyclooxygenase-2 (COX-2) expression also appeared to be reduced in osteoclasts from the *Postn*HET, *Postn*KO, and *Spp1*KO calluses, including in those osteoclasts localized at the chondro-osseous junction. The results indicate that POSTN and SPP1 are necessary for normal chondro-osseous junction formation and that signaling from the chondro-osseous junction, possibly from COX-2 expressing osteoclasts, regulates callus vasculogenesis and chondrocyte hypertrophy necessary for endochondral ossification during fracture healing.

## Impact statement

Impaired healing in *Postn*-null and *Spp1*-null mice provided evidence for a regulatory role of the callus chondro-osseous junction in bone fracture healing. Delayed callus cartilage resolution and reduced callus vasculogenesis in *Postn*-null and *Spp1*-null calluses were associated with abnormal chondro-osseous junction morphology, reduced transition of chondrocytes into MMP13 expressing hypertrophic chondrocytes, and reduced COX-2 expression in chondro-osseous junction osteoclasts. The results indicate that the chondro-osseous junction is not just the site at which callus cartilage is resorbed prior to bone formation but that the chondro-osseous junction has a critical regulatory role in endochondral ossification. Loss of *Postn* or *Spp1* reduced expression of the other gene, suggesting that expression of these matricellular proteins is coordinated during fracture healing and that *Postn* and *Spp1* are important for normal chondro-osseous functioning. The results advance our understanding of matricellular proteins in bone regeneration and identify new roles for the chondro-osseous junction in endochondral ossification.

## Introduction

Bone fractures normally heal through tissue regeneration [1]. Immediately after fracture, a hematoma forms at the site which is accompanied by local tissue hypoxia as factors are released from degranulating platelets and from damaged nerves located on or within the bone [2–4]. An inflammatory response quickly follows and the fracture site is rapidly populated with myeloid and other cells that have migrated to or proliferated at the fracture site to form the presumptive callus [5]. New bone is then directly made by periosteal cells to form buttresses of bone ringing the external, peripheral edges of the callus. Within the now well-defined external callus, cells differentiate into chondrocytes and a chondro-osseous junction is formed between the bony peripheral edges of the callus and callus chondrocytes. Osteoclasts localize at the chondro-osseous junction to form a margin at which endochondral bone formation initiates [6].

Endochondral ossification is characterized by chondrocyte hypertrophy adjacent to the chondro-osseous junction, destruction of the hypertrophic chondrocytes and associated extra-cellular matrix, vasculogenesis, and osteoblast-mediated bone formation [7, 8]. Vasculogenesis is necessary for endochondral ossification during fetal bone development, bone growth, and fracture healing [9, 10]. The osteoclasts at the chondro-osseous junction, sometimes called chondroclasts, are thought to destroy the hypertrophic cartilage matrix and enable vasculogenesis necessary for fracture healing [11]. As healing progresses, the two chondro-osseous junctions, one proximal and one distal to the fracture, advance from the callus peripheries as endochondral ossification continues. Eventually, the chondro-osseous junctions converge to bridge the fracture with new bone. Bony bridging of the fracture results in a significant increase in bone structural mechanics. The external callus decreases in size as woven bone within the callus undergoes osteoclast-mediated remodeling into more mechanically stable lamellar bone until the fracture is fully healed.

The molecules and mechanisms necessary to regulate the temporal and spatially overlapping physiological, cellular, and molecular processes governing fracture healing are not well defined [12–14]. While the roles of certain cytokines, lipid mediators, and growth factors in fracture healing have been studied extensively [13], the roles of non-structural extracellular matrix proteins, or matricellular proteins, in bone fracture healing are less clear. Matricellular proteins interact with other extracellular matrix components, growth factors, and cell surface receptors to affect cell signaling, cell-cell interactions, and the local tissue environment [15, 16]. As such, matricellular proteins may act to coordinate the cellular and molecular processes involved in fracture healing.

Periostin (POSTN) and osteopontin (SPP1) are matricellular proteins expressed in the fracture callus and loss of *Spp1* or *Postn* in mice leads to fracture healing deficits [17–20]. Mice that are homozygous for a targeted null mutation of *Postn* are viable and fertile, though with notable phenotypes including reduced bone quality [21]. Mice that are homozygous for a targeted null mutation of *Spp1* are also viable and fertile [22]. *Postn* and *Spp1* are associated with multiple physiological processes that can affect fracture healing including inflammation, chondrogenesis, osteogenesis, vasculogenesis, and osteoclastogenesis [19, 23–29].

*Spp1* mRNA and POSTN are localized in hypertrophic chondrocytes at the callus chondro-osseous junction and SPP1 and POSTN are integrin αvß3 ligands [20, 30–32]. Osteoclasts abundantly express integrin αvß3 and are localized at the callus chondro-osseous junction [33, 34]. The proximity of hypertrophic chondrocytes expressing SPP1 and POSTN and osteoclasts expressing integrin αvß3 at the callus chondro-osseous junction suggests that *Spp1* or *Postn* can affect fracture healing by regulating events at the chondro-osseous junction. To test this hypothesis, we undertook a detailed analysis of the morphological events that occur during femur fracture healing in normal mice and mice deficient in *Postn* or *Spp1*. Our analysis indicates that *Postn* and *Spp1* are necessary for formation of a normal callus chondro-osseous junction and that reductions in callus vasculogenesis and chondrocyte hypertrophy in *Postn* or *Spp1* deficient mice are associated with reduced osteoclast cyclooxygenase-2 (COX-2) expression.

## Materials and methods

### Animal models

Mice with a targeted null mutation in the periostin gene (*Postn* ^tm1.1(KOMP)Vlcg^) were purchased from Jackson Laboratory (Stock #024186, Bar Harbor, ME). Homozygous *Postn* ^tm1.1(KOMP)Vlcg^ null mice (*Postn*KO) are viable and fertile but with reduced mechanical integrity in connective tissues [35, 36]. The *Postn* ^tm1.1(KOMP)Vlcg^ mice were bred with C57BL/6 mice to produce normal (WT), *Postn* ^tm1.1(KOMP)Vlcg^ heterozygous (*Postn*HET), and *Postn*KO mice.

Mice with a targeted null mutation in the osteopontin gene (*Spp1* ^tm1Blh^) were purchased from Jackson Laboratory (Stock #004936, Bar Harbor, ME) [22]. The *Spp1* ^tm1Blh^ null mice (*Spp1*KO) have a C57BL/6 genetic background and were maintained by homozygous null breeding to produce *Spp1*KO mice. Mice were genotyped via allele-specific PCR amplification of DNA extracted from tail clip biopsies (Table 1). Immunohistochemistry was conducted to confirm loss of *Postn* and *Spp1* protein expression in the fracture calluses of homozygous null mice. Six to fourteen mice were used for each genotype at each time point of which at least 3 were males. All animal procedures were approved by the Rutgers-New Jersey Medical School Institutional Animal Care and Use Committee (IACUC; protocol #201800006).

**TABLE 1:** Study oligodeoxynucleotide primers.

Genotyping primers			
Gene allele	Designation	Forward	Reverse
*Spp1^tm1Blh^*	*Spp1*KO	GCC​TGA​AGA​ACG​AGA​TCA​GC	GTC​TGG​AGA​ACA​TGG​GTG​CT
*Spp1*	WT	GGG​TGC​AGG​CTG​TAA​AGC​TA	GTC​TGG​AGA​ACA​TGG​GTG​CT
*Postn^tm1.1(KOMP)Vlcg^*	*Postn*KO	CGG​TCG​CTA​CCA​TTA​CCA​GT	CAG​TTC​CTA​CCC​CAC​AGG​AG
*Postn*	WT	CAT​CCT​AAA​TAC​CCT​CCA​GTG​C	GGA​CTT​CAT​CAA​TCA​GGT​GGA

RTqPCR Primers			
Gene	Protein	Forward	Reverse
*Actb*	ß-actin	GGG​CTA​TGC​TCT​CCC​TCA​CG	AGACGAACATAGCACAGCTTC TCTT
*Mmp-13*	Matrix Metallopeptidase 13	GAG​TGC​CTG​ATG​TGG​GTG​AAT	CCAGAAGGTCCATCAAATGGG T
*Acp5*	Tartrate-resistant Acid Phosphatase 5	GGT​TCC​AGG​AGA​CCT​TTG​AG	TTCCAGCCAGCACATACC
*Ctsk*	Cathepsin-K	AGGCAGCTAAATGCAGAGGGT ACA	AGC​TTG​CAT​CGA​TGG​ACA​CAG AGA
*Acan*	Aggrecan	CAT​GAG​AGA​GGC​GAA​TGG​AA	TGATCTCGTAGCGATCTTTCTT CT
*Pecam1*	CD-31	CCAAAGCCAGTAGCATCATGG TC	GGATGGTGAAGTTGGCTACAG G
*Ptgs2*	Cyclooxygenase-2	GGG​CAG​GAA​GTC​TTT​GGT​C	GGT​AAC​CGC​TCA​GGT​GTT​G
*B2m*	ß-2-microglobulin	CTGCTACGTAACACAGTTCCA CCC	CATGATGCTTGATCACATGTCT CG
*Postn*	Periostin	CCT​GCC​CTT​ATA​TGC​TCT​GCT	AAACATGGTCAATAGGCATCA CT
*Spp1*	Osteopontin	GCA​GCC​ATG​AGT​CAA​GTC​AGC	GCC​TCT​TCT​TTA​GTT​GAC​C
*Bglap*	Osteocalcin	CCA​TGA​GGA​CCA​TCT​TTC​TGC	CAG​GTC​CTA​AAT​AGT​GAT​ACC

### Fracture procedure and radiography

Male and female mice aged 15 weeks were anesthetized by intraperitoneal injection of ketamine and xylazine (0.1 and 0.01 mg/g body weight, respectively) prior to retrograde insertion of 0.01-inch diameter stainless steel pin in the right femoral canal to stabilize the impending fracture. At 16 weeks of age, the mice were anesthetized again and underwent a closed, diaphyseal fracture of the right femur using a custom-made, three-point controlled impact device (BBC Specialty Automotive Center, Linden, NJ) as described previously [37]. For all genotypes, male mice weighed significantly more than the females.

C57BL/6 and *Spp1*KO mice had significantly higher body weights than *Postn*KO mice. C57BL/6 mice weighed 23.17 ± 3.70 g (mean ± SD), *Spp1*KO 24.42 ± 3.23 g (mean ± SD), *Postn*KO 21.28 ± 3.19 g (mean ± SD) and *Postn*HET 22.47 ± 3.89 g (mean ± SD). Ventral-dorsal radiographs of the mice were made using an XPERT80 digital radiography cabinet (KUBTEC, Stratford, CT) with exposure settings of 65 kV, 90 µA, and 8 s immediately after fracture and then at 7, 10, 14, 21, and 28 days after fracture (dpf) or until the endpoint (see Supplementary Figure S1). Fixed femurs (see below) were stored in 70% ethanol prior to high-resolution computerized tomography (µCT) scanning. Specimens were scanned using a Bruker Skyscan 1275 system (Micro Photonics Inc., Allentown, PA) at 73 kVp, with an intensity of 133 µA, a voxel size of 12 μm isotropic, and with a 0.5 mm aluminum filter. Scanner images were reconstructed (NRecon), analyzed (CTan), and viewed (CTvol) using software from the manufacturer (Bruker, Kontich, Belgium). A detailed description of the method used to measure fracture callus volume and fracture callus bone (calcified tissue) volume can be found in the Supplementary Material (see Supplementary Figure S2).

### Histology

After euthanasia, femurs were resected and then fixed in an aqueous solution of bronopol (3% w/v), diazolidinyl urea (3% w/v), zinc sulfate hepta-hydrate (1.2% w/v), sodium citrate (0.29% w/v), ascorbic acid (0.025% w/v), and 20% (v/v) ethanol for 2 days at room temperature. The specimens were then either immediately decalcified in 0.5 M disodium EDTA for 2 weeks at 4°C or analyzed by µCT before decalcification. The decalcified specimens were embedded in paraffin, cut into 5 µm thick longitudinal sections parallel to the intramedullary canal in the dorsal-ventral plane, and mounted onto TruBond 380 glass slides (Newcomer Supply, Middleton, WI). Mounted sections were deparaffinized using three changes of xylene and rehydrated in a graded ethanol series. Osteoclasts were detected by tartrate-resistant acid phosphatase (TRAP) staining with a hematoxylin counterstain. Cartilage was visualized by safranin-O staining with fast green and hematoxylin counterstaining. Bone was visualized using aniline blue staining with Biebrich scarlet-acid fuchsin and hematoxylin counterstaining (Masson’s trichrome staining). Sections were cover-slipped with Cytoseal mounting medium (Fisher Scientific, Waltham, MA). Unless otherwise indicated, stains and reagents were from Sigma-Aldrich (St. Louis, MO).

### Immunohistochemistry (IHC)

Antibodies used are listed in Table 2. Tissue sections were deparaffinized and rehydrated as described above before antigen retrieval in 10 mM sodium citrate pH 6.0 buffer (70–80°C, 1 h) for COX-2, CD31, POSTN, SPP1, F4/80 and Cathepsin K (CTSK) detection, or in phosphate-buffered saline (PBS) with 25 mg/mL testicular hyaluronidase (37°C for 1 h; Worthington Biochemical Corporation, Lakewood, NJ) for MMP-13 and Collagen X detection. Endogenous peroxidases were quenched with 3% H_2_O_2_ in PBS (30 min at room temperature) followed by non-specific epitope blocking with SuperBlock (room temperature for 1 h; ThermoFisher, Waltham, MA). After washing with PBS, 150 µL of primary antibody diluted in Antibody Diluent pH7.4 (IHC World, Woodstock, MD) was applied to each histological section and incubated overnight in a humidified chamber at 4°C. See Table 2 for antibody dilutions. Following several washes in PBS, sections were incubated in POLINK-2 Plus Rabbit polymeric HRP secondary antibody per the manufacturer’s instructions (IHC World) and then washed with PBS before colorimetric detection using diaminobenzidine (GBI Labs, Bothell, WA). Sections were counterstained with methyl green to detect cell nuclei.

**TABLE 2:** Study rabbit antibodies.

Antibody target	Company	Catalog number	Type	Dilution	Antigen retrieval
CTSK (cathepsin K)	Novus Biologicals	NBP1-45460	PAb	1:100	10 mM sodium citrate, 1 h, 75°C
CD-31	Cell Signaling	77699	MAb	1:500	10 mM sodium citrate, 1 h, 75°C
Collagen X	abcam	ab260040	MAb	1:1,000	25 mg/mL hyaluronidase, 1 h, 37°C
COX-2	Cayman	160,126	PAb	1:700	10 mM sodium citrate, 1 h, 75°C
F4/80	Cell Signaling	70076	MAb	1:1,000	10 mM sodium citrate, 1 h, 75°C
MMP-13	abcam	ab219620	MAb	1:100	25 mg/mL hyaluronidase, 1 h, 37°C
SPP1 (osteopontin)	Novus Biologicals	NB600-1043	PAb	1:50	10 mM sodium citrate, 1 h, 75°C
POSTN (periostin)	Sino Biological	50450-RP02	PAb	1:5,000	10 mM sodium citrate, 1 h, 75°C

### Histology and immunohistochemistry image collection and analysis

Digital images of callus fracture sections were captured using an Olympus BX53 microscope and DP73 camera (Olympus Corporation of America, Center Valley, PA). Cartilage and bone areas were measured for each specimen using OsteoMeasure Software (OsteoMetrics Inc., Decatur, GA) from safranin-O and trichrome stained sections, respectively, and normalized as a percentage of total callus area. TRAP positive cells were manually counted using OsteoMeasure and divided by callus area to generate the density of TRAP positive cells per mm^2^. Similar procedures for image collection and analysis were conducted with the IHC samples to determine COX-2, Cathepsin K (CTSK), MMP-13, CD31, Collagen X (COL10A1), and F4/80 positive areas or cell numbers in the callus. Examples showing how MMP-13 expressing chondrocytes and vascular lumens surrounded by CD31 expressing cells were counted are shown in Supplementary Figure S3. A comparison between using TRAP staining and IHC detection of CTSK to count callus osteoclasts showed a high degree of correlation (R^2^ = 0.91, see Supplementary Figure S4).

### Fracture callus RNA isolation, cDNA synthesis, and qPCR

Fracture calluses were resected at 14 days after fracture (dpf) from at least 3 male and 3 female mice of each genotype. Calluses were flash-frozen in liquid nitrogen and stored at −80°C until RNA extraction [38]. Briefly, each callus was homogenized in Trizol Reagent (1 mL per 50 to 100 mg callus weight) using a Precellys 24 Omni Bead Homogenizer (Bertin Technologies SAS; Montigny-le-Bretonneux, France). After Trizol extraction, the RNA was further purified using a Qiagen RNeasy mini kit as per the manufacturer’s instructions (Qiagen, Hilden, Germany). RNA concentration and integrity were determined by absorbance and agarose gel electrophoresis, respectively.

Target mRNA levels were measured using RT-qPCR as follows. cDNA was prepared from each RNA preparation using an Applied Biosystems High-Capacity cDNA kit (Waltham, MA). The cDNA from 0.1 µg of total RNA along with 1 µM of each primer were used in each 25 µL qPCR reaction (SYBR Green PCR Master Mix, Applied Biosystems 4309155). Amplifications were performed using a Bio-Rad Laboratories CFX96 Real-Time System (Hercules, CA). At least 3 qPCR reactions were performed with each cDNA preparation for every target mRNA and the mean threshold cycle (Ct) value was used for further comparisons. At least 6 callus RNA preparations (3 male and 3 female) were measured for each genotype and for each target mRNA. Target mRNA levels were normalized to corresponding β-actin mRNA Ct values. Relative gene expression was determined using the 2^−ΔΔCT^ method [39].

### Statistical analyses

Statistical analyses were performed using OriginPro 2022b (OriginLab Corp., Northhampton, MA). Callus histomorphometry data, including the number of TRAP positive+ cells, cartilage area, bone area, and all immunohistochemistry gene quantifications were analyzed by 3-way ANOVA using genotype, days post-fracture (dpf) and gender as independent variables. Post-hoc tests utilized Tukey or Holm-Sidak corrections to identify significant differences between groups. Detailed information regarding the statistical analyses is shown in the Supplementary Material.

## Results

### Loss of *Postn* or *Spp1* affects fracture callus formation and remodeling

**FIGURE 1:**
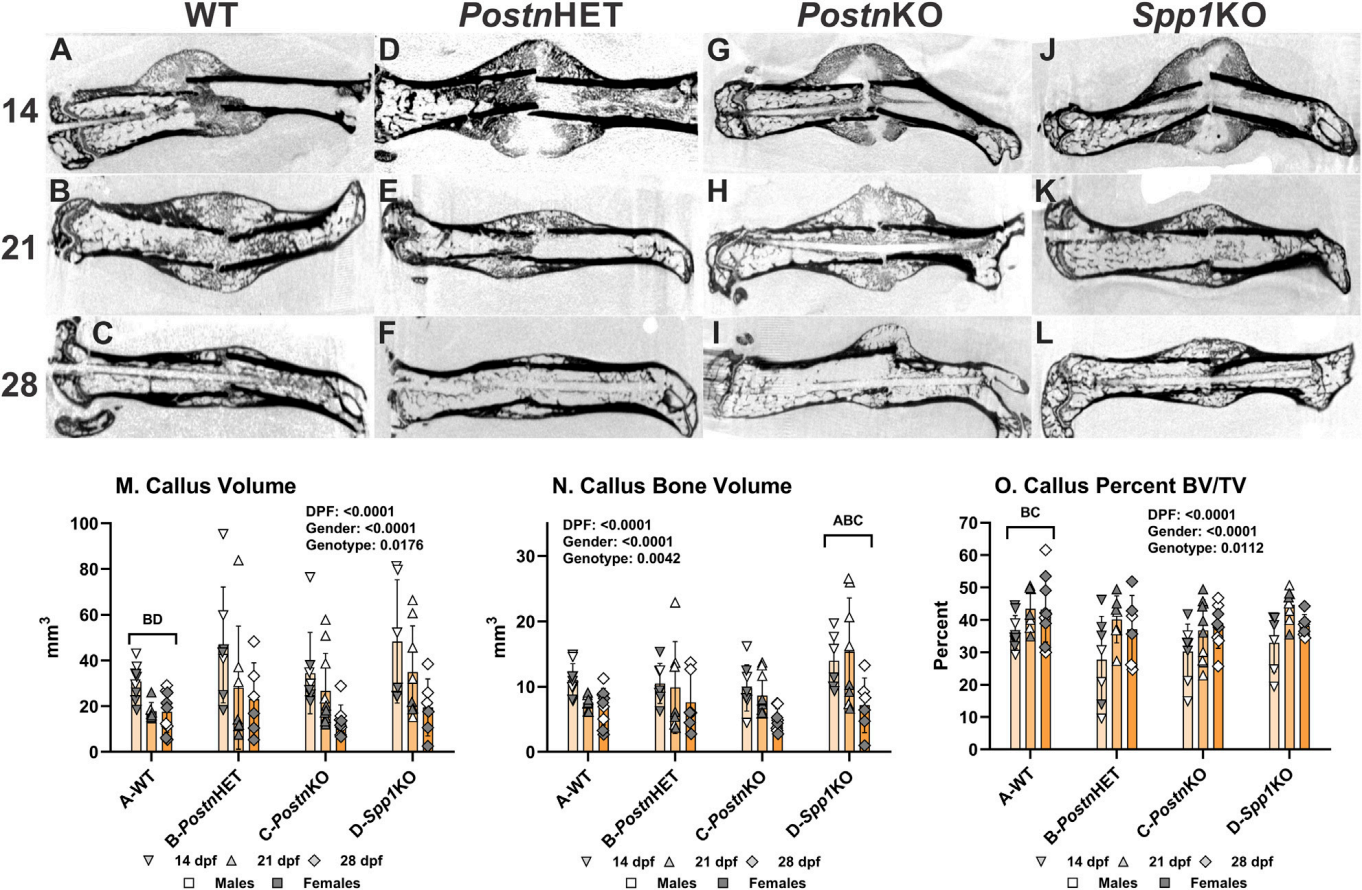
µCT Analysis of Fracture Callus Bone in *Postn* and *Spp1* Deficient Mice. Fractured mouse femurs were collected from C57BL/6 (WT), *Postn* ^+/−^ (*Postn*HET), *Postn* ^−/−^ (*Postn*KO), and *Spp1* ^−/−^ (*Spp1*KO) mice and imaged by µCT scanning. Shown are representative images from the reconstructed fractured femur scans at 14, 21, and 28 dpf for WT **(A–C)**, *Postn*HET **(D–F)**, *Postn*KO **(G–I)**, and *Spp1*KO mice **(J–L)**, respectively. Callus total volume [TV, panel **(M)**], bone volume [BV, panel **(N)**] and normalized bone volume [BV/TV, panel **(O)**] were determined from µCT imaging data collected from at least three female and three male mice of each genotype and at each time point. The effects of time after fracture, gender, and genotype were determined by 3-way ANOVA and P-values are reported [insets, panels **(M–O)**]. Significant differences between genotypes (p < 0.05) are noted in each graph.

µCT was performed on femurs resected at 14, 21, and 28 days post-fracture (dpf) to visualize and quantify morphological differences in the facture calluses between wild type (WT), *Postn*HET, *Postn*KO, and *Spp1*KO genotypes (see Supplementary Figure S1 for digital radiographs). Longitudinal sections from reconstructed µCT volumes are shown for each time point and genotype in Figure 1. Healing appeared to proceed normally in the WT control C57BL/6 mice (Figures 1A–C) with apparent bridging at 14 dpf, definitive bridging by 21 dpf, and callus remodeling evident at 28 dpf [37]. Healing also appeared to proceed reasonably well in the *Postn*HET mice with definitive bridging by 21 dpf and evident callus remodeling at 28 dpf, though at 14 dpf less calcified tissue was evident in the external callus (Figures 1D–F). The external fracture callus in the *Postn*KO and *Spp1*KO mice at 14 dpf appeared to have central areas devoid of calcified tissue (Figures 1G, J). By 21 dpf, the *Postn*KO and *Spp1*KO calluses appeared to bridge the fracture (Figures 1H, K). Callus remodeling was evident at 28 dpf in the *Postn*KO and *Spp1*KO mouse fracture calluses (Figures 1I, L). However, less bone was evident in the 28 dpf *Postn*KO callus and the extent of callus remodeling appeared reduced for both genotypes.

The µCT data were also used to quantify total callus volume (TV) and callus bone (calcified tissue) volume (BV). As expected, TV, BV, and BV/TV varied with time after fracture for all genotypes showing a general pattern of decreasing callus TV (means of 39, 27, and 18 mm^3^ at 14, 21, and 28 dpf) and BV (means of 11, 10, and 6.5 mm^3^ at 14, 21, and 28 dpf) but increasing BV/TV from 14 dpf (means of 32, 41, and 39% at 14, 21, and 28 dpf; Figures 1M–O). In addition, male mice also had significantly larger calluses (TV; means of 37 vs. 19 mm^3^) and more callus bone (BV; means of 11.5 vs. 7.4 mm^3^), though normalized callus bone volume (BV/TV) was in general greater in the female mice (means of 34% vs. 41%; see Supplementary Material). Across all time points and both genders, callus TV was significantly greater in the *Postn*HET (33.1 mm^3^) and *Spp1*KO (34.3 mm^3^) as compared to the WT (22.4 mm^3^) and *Postn*KO (25.1 mm^3^) mice. Consistent with the overall callus volume, BV was significantly greater in the *Spp1*KO mice (12.5 mm^3^) and lowest in the *Postn*KO mice (7.8 mm^3^), though differences between WT (8.6 mm^3^), *Postn*HET (9.4 mm^3^), and *Postn*KO were not significant. In contrast, BV/TV across all time points and both genders was significantly lower in the *Postn*HET (34.9%) and *Postn*KO (35.1%) mice as compared to the WT mice (40.8%), though no statistical difference was detected for the *Spp1*KO mice (39.2%).

### SPP1 and POSTN expression in normal, *Postn*-deficient, and *Spp1*-deficient fracture calluses

**FIGURE 2:**
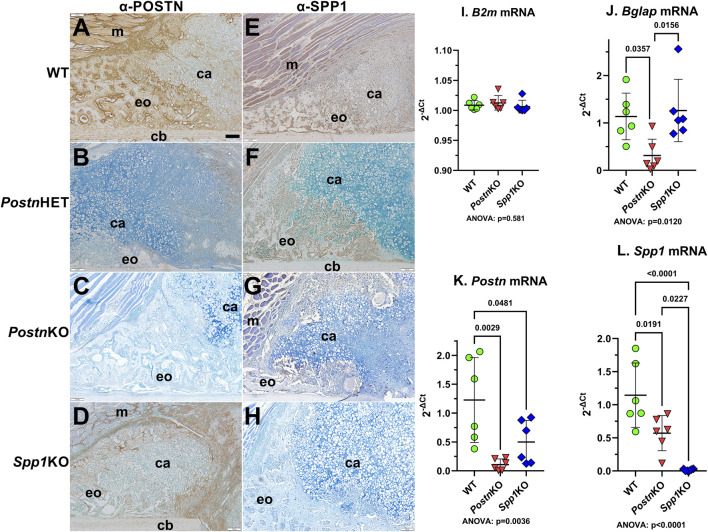
Immunohistochemical Detection of POSTN and SPP1 in normal and *Postn* or *Spp1* deficient mouse femur fracture calluses. Mouse femurs collected at 10 dpf were used to detect POSTN **(A–D)** or SPP1 **(E–H)** in WT **(A, E)**, *Postn*HET **(B, F)**, *Postn*KO **(C, G)** or *Spp1*KO **(D, H)** calluses. The images show approximately one-quarter of the fracture callus and are oriented with the femur cortical bone at the bottom and fracture site in the bottom right corner. Muscle (m), cartilage (ca), cortical bone (cb), and areas of apparent new bone formation (eo) are labeled. POSTN and SPP1 were readily detected in the WT mouse calluses [**(A, E)**, respectively]. POSTN expression appeared reduced in the *Postn*HET callus **(B)**, while SPP1 expression appeared to be near normal levels **(F)**. POSTN expression was not detected in the *Postn*KO callus **(C)** which also appeared to have reduced levels of SPP1 though with some expression evident in callus chondrocytes **(G)**. POSTN appeared less abundant in the *Spp1*KO callus **(D)** as compared to WT **(A)** but was still readily detected. As expected, SPP1 was not detected in the *Spp1*KO callus **(H)**. RTqPCR quantitation of control ß2 microglobulin [*B2m*; **(I)**], osteocalcin [*Bglap*; **(J)**], *Postn* **(K)**, and *Spp1* **(L)** mRNA levels in callus RNA prepared from three male and three female WT, *Postn*KO, or *Spp1*KO mice at 14 dpf paralleled the immunohistochemical detection results. *Postn* and *Spp1* mRNA levels appeared absent from their corresponding KO callus RNA preparations. *Postn* mRNA was also significantly reduced in the *Spp1*KO callus RNA while *Spp1* mRNA was significantly reduced in the *Postn*KO callus RNA. ANOVA and post-hoc P values are shown in **(I, J, K, L)**. Scale bar equals 100 µm **(A)**.

Immunohistochemistry was performed on 10 dpf calluses to confirm normal expression of POSTN and SPP1 in WT mice and loss of POSTN and SPP1 expression in *Postn*KO and *Spp1*KO mice, respectively (Figure 2).

POSTN was broadly expressed throughout the WT callus (Figure 2A). POSTN levels appeared higher in osteoblast-rich areas and along the chondro-osseous junction with apparent lower levels in callus chondrocytes. POSTN expression levels appeared to be reduced in the *Postn*HET callus with POSTN localization limited to fibroblasts and newly differentiated chondrocytes at the center of the callus and to chondrocytes near the chondro-osseous junction (Figure 2B). As expected, POSTN was absent in the *Postn*KO callus (Figure 2C). POSTN expression was detected in the *Spp1*KO callus though at an apparent reduced level as compared to WT (Figure 2D).

SPP1 was also broadly expressed throughout the WT fracture callus with SPP1 localized in callus osteoblasts and chondrocytes (Figure 2E). As expected, SPP1 expression appeared absent in the *Spp1*KO callus (Figure 2H). SPP1 levels appeared reduced in the *Postn*HET callus with expression in osteoblasts and in a subset of chondrocytes at or near the chondro-osseous junction (Figure 2F). SPP1 expression in the *Postn*KO callus appeared to be even further reduced as compared to the *Postn*HET callus (Figure 2G).

**FIGURE 3:**
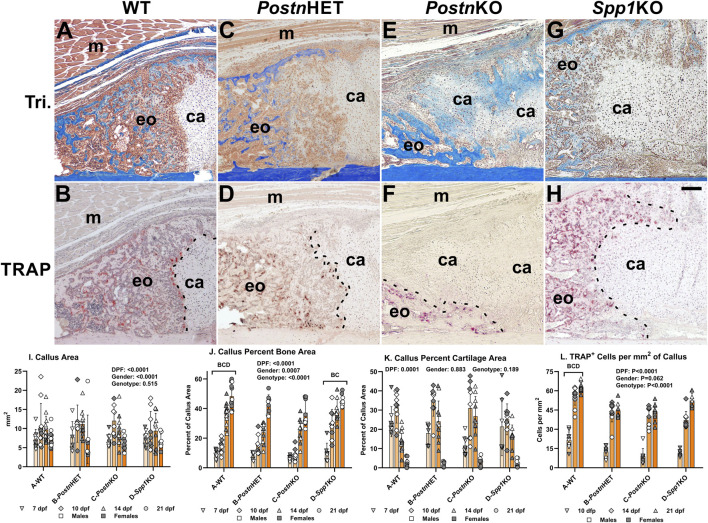
Histomorphometric Analysis of *Postn* and *Spp1* Deficient Mouse Fracture Calluses. Longitudinal sections from 14 dpf mouse femur fracture calluses stained with Masson’s trichrome **(A, C, E, G)** or for tartrate resistant acid phosphatase (TRAP) activity **(B, D, F, H)** are shown from WT **(A, B)**, *Postn*HET **(C, D)**, *Postn*KO **(E, F)**, and *Spp1*1KO mice **(G, H)**. The images show approximately one-quarter of the fracture callus and are oriented with the femur cortical bone at the bottom and fracture site in the bottom right corner. Muscle (m), cartilage (ca), and areas of apparent new bone formation (eo) are labeled. The chondro-osseous junction is highlighted with a dashed line in panels **(B, D, F, H)**. Femur fracture calluses were used to measure callus area **(I)**, normalized callus bone area **(J)**, and normalized callus cartilage area **(K)** at 7, 10, 14, and 21 dpf. Cartilage was identified by safranin-O staining (not shown). Tartrate resistant acid phosphatase (TRAP) staining was used to identify and count callus TRAP^+^ cells at 10, 14, and 21 dpf **(L)**. The effect of time after fracture, gender, and genotype were determined by 3-way ANOVA and P-values are noted [insets, **(I, J, K, L)**]. Differences between genotypes are noted in each graph. Scale bar equals 100 µm **(H)**.

*Postn* and *Spp1* mRNA levels were measured in total RNA prepared from 14 dpf WT, *Postn*KO, and *Spp1*KO calluses by RTqPCR. As expected, ß-2-microglobulin (B2m) mRNA levels were similar between calluses from all genotypes (Figure 2I), while *Postn* and *Spp1* mRNA levels were significantly reduced in the *Postn*KO and *Spp1*KO calluses, respectively (Figures 2K, L). Similar to the IHC results, *Spp1* mRNA and *Postn* mRNA levels were significantly lower than WT levels in the *Postn*KO and *Spp1*KO calluses, respectively. We also noted that osteocalcin (*Bglap*) mRNA levels were reduced in the *Postn*KO mouse calluses (Figure 2J), which was consistent with the reduced callus BV/TV (Figure 1O) and reduced callus bone area (Figure 3J) in *Postn* deficient mice.

### Loss of *Postn* or *Spp1* affects fracture callus morphology

Masson’s trichrome staining was performed on tissue sections of fracture calluses collected at 7, 10, 14, and 21 dpf to visualize morphological differences between mouse genotypes during the healing process. Images from one quadrant of the external fracture callus of a WT, *Postn*HET, *Postn*KO, and *Spp1*KO mouse at 14 dpf are shown in Figures 3A, C, E, G, respectively. External callus bone tissue and cartilage were identified based on staining and morphology and quantified at each time point as shown in Figures 3I–K. Similarly, TRAP staining was performed on serial sections to identify osteoclasts in the fracture calluses (Figures 3B, D, F, H). At 14 dpf, the external callus of the WT mouse has apparent newly formed bone, is highly cellularized, and has a clearly demarcated chondro-osseous junction that is lined with osteoclasts (TRAP^+^ cells; Figures 3A, B). While the 14 dpf *Postn*HET callus also appears to have a considerable amount of new bone and is highly cellularized, the chondro-osseous junction is neither distinct nor abundantly populated with osteoclasts (Figures 3C, D). The *Postn*KO mouse callus appears to have less new bone, more cartilage, and a poorly defined chondro-osseous junction (Figure 3E, F). The *Spp1*KO mouse callus appears to have amounts of new bone and cartilage that are comparable to the WT callus (Figures 3G, H).

Similar to the *Postn*HET and *Postn*KO chondro-osseous junctions though, the *Spp1*KO chondro-osseous junction also appears to have fewer osteoclasts and displays an abnormal morphology.

Callus area, percent bone area, percent cartilage area, and density of TRAP^+^ cells were quantified at each time point and for each genotype as shown in Figures 3I–L. As expected, callus area was dependent upon time after fracture and gender but was not dependent upon genotype.

For all genotypes, callus percent bone area increased with time after fracture (Figure 3J) while callus percent cartilage area peaked at 10 dpf before declining (Figure 3K). Callus percent bone area was dependent upon time after fracture, gender, and genotype with WT significantly different from all other genotypes and *Spp1*KO different from *Postn*HET and *Postn*KO. In contrast, callus percent cartilage area was only dependent upon on time after fracture and not gender or genotype. The density of osteoclasts (TRAP^+^ cells/mm^2^) was dependent upon time after fracture and genotype, but not gender (Figure 3L). Osteoclast density rapidly increased between 10 and 14 dpf for all genotypes but overall osteoclast density was greater in the WT mouse fracture calluses than in the *Postn*HET, *Postn*KO, or *Spp1*KO mouse calluses.

### Loss of *Postn* or *Spp1* alters chondrocyte hypertrophy

**FIGURE 4:**
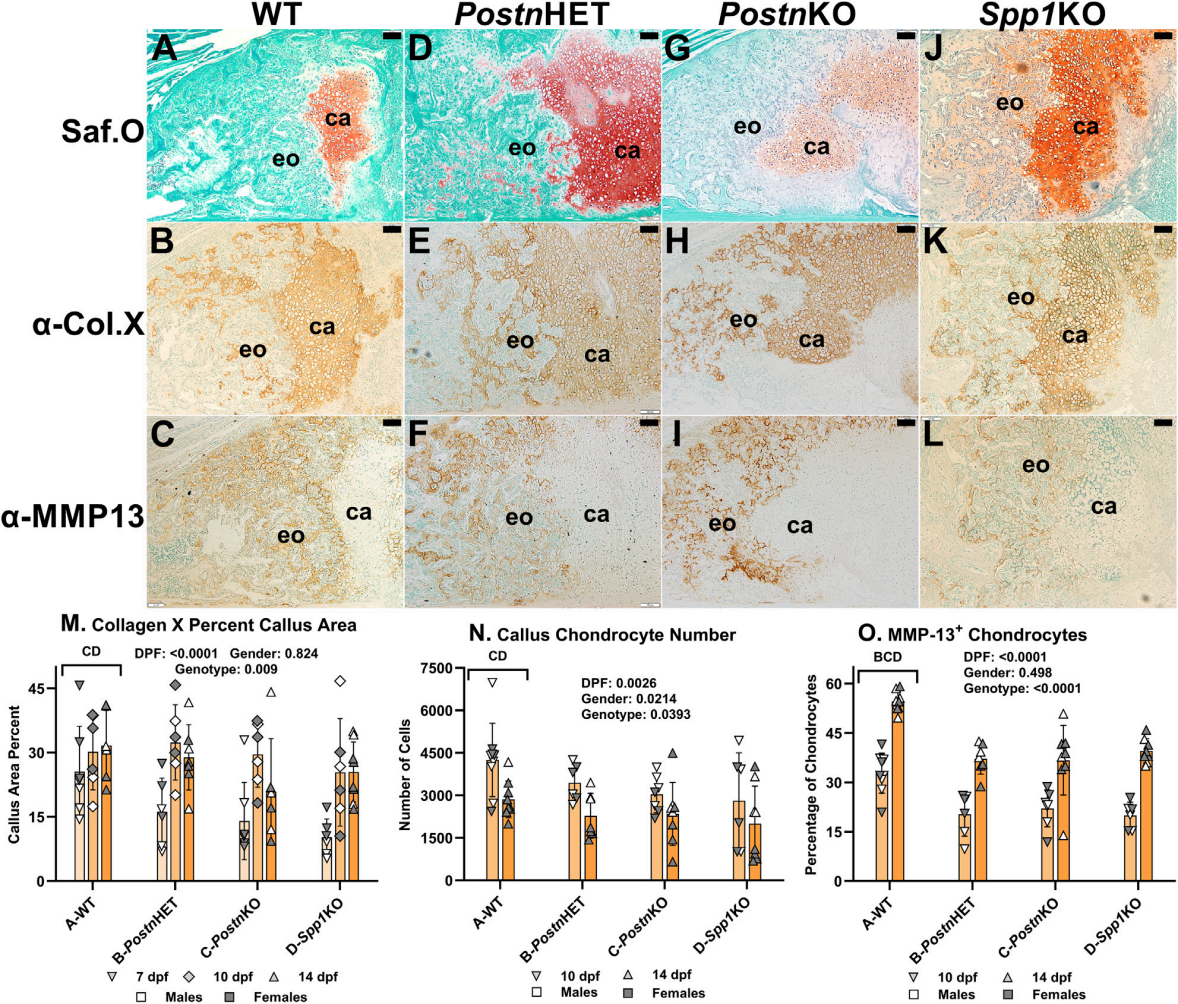
Immunohistochemical Analysis of Chondrocyte Hypertrophy in *Postn* and *Spp1* Deficient Mouse Fracture Calluses. Longitudinal sections from 14 dpf mouse femur fracture calluses were stained with safranin-O and fast green **(A, D, G, J)** or used to detect Collagen X **(B, E, H, K)** and MMP-13 **(C, F, I, L)** by immunohistochemistry. Specimens were from WT **(A–C)**, *Postn*HET **(D–F)**, *Postn*KO **(G–I)** and *Spp1*KO **(J–L)** mice. Areas of callus cartilage (ca) and endochondral ossification (eo) are noted. Collagen X expression (brown) was prominently detected around callus cartilage chondrocytes but also around chondrocytes in areas of endochondral bone formation for all genotypes. In contrast, MMP-13 expression (brown) was detected around chondrocytes in areas of endochondral ossification but rarely around chondrocytes in the callus cartilage. Quantitative analysis found reduced collagen X staining area, numbers of chondrocytes, and MMP-13^+^ chondrocytes in *Postn*KO and *Spp1*KO mouse calluses **(M–O)**. The number of MMP-13^+^ chondrocytes also was significantly reduced in the *Postn*HET specimens as compared to WT **(O)**. The effects of time after fracture, gender, and genotype were determined by 3-way ANOVA and P-values are shown in each graph [insets, **(M–O)**]. Scale bars equal 100 µm.

The effects of *Postn* and *Spp1* null mutations on callus chondrocyte hypertrophy were assessed by immunohistochemical (IHC) detection and quantification of Collagen X (COL10A1) and Matrix Metallopeptidase-13 (MMP13). Serial sections from mouse fracture calluses collected at 7, 10, or 14 dpf were stained with Safranin-O to detect cartilage (Figures 4A, D, G, J, see also Figure 3K) or used for IHC detection of collagen X (Figures 4B, E, H, K) or MMP-13 (Figures 4C, F, I, L). Safranin-O stained areas appeared to roughly correlate with callus areas expressing collagen X. In contrast, callus cells expressing MMP-13 were generally not evident in callus cartilage areas stained with Safanin-O. Instead, cells expressing MMP-13 also appeared to express collagen X and were localized in areas of newly forming bone.

The percentage of callus cartilage area that expressed collagen X was determined at 7, 10, and 14 dpf (Figure 4M). Callus area expressing collagen X was dependent upon time after fracture and mouse genotype, but not gender. Across all time points, the percent of callus area that stained for COL10A1 by IHC was significantly lower for the *Postn*KO (21%) and *Spp1*KO (21%) mouse calluses as compared to WT (29%; p < 0.009).

Callus chondrocytes were counted at 10 and 14 dpf (Figure 4N) and compared to the number of callus chondrocytes expressing MMP-13, and thus presumably undergoing hypertrophy (Figure 4O). The number of callus chondrocytes was significantly reduced in the *Postn*KO and *Spp1*KO mouse calluses as compared to WT (p = 0.039). The number of callus chondrocytes also decreased between 10 dpf to 14 dpf by 33% in WT, 34% in *Postn*HET, 23% in *Postn*KO, and 29% in *Spp1*KO calluses. Conversely, the percentage of callus chondrocytes expressing MMP-13 increased between 10 and 14 dpf for all genotypes (p < 0.0001), even though the percentage of MMP-13 expressing chondrocytes was significantly lower in the *Postn*HET, *Postn*KO, and *Spp1*KO mouse calluses as compared to WT (p < 0.0001).

### Fracture callus vascularization is reduced by loss of *Postn* or *Spp1*

**FIGURE 5:**
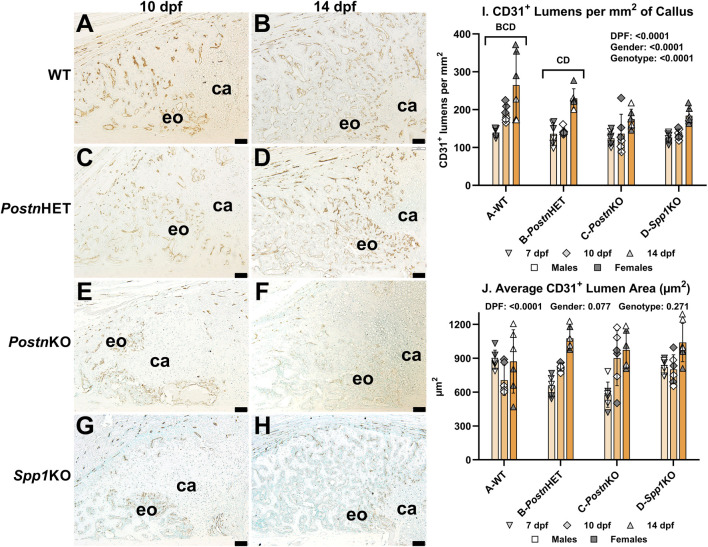
Immunohistochemical Assessment of Fracture Callus Vasculogenesis in *Postn* and *Spp1* Deficient Mice. Endothelial cells were identified in femur fracture callus longitudinal sections by immunohistochemical staining for CD31 (*Pecam1*) at 7 (not shown), 10 **(A, C, E, G)**, and 14 dpf **(B, D, F, H)** and from WT **(A, B)**, *Postn*HET **(C, D)**, *Postn*KO **(E, F)** and *Spp1*KO mice **(G, H)**. Avascular callus cartilage (ca) and vascularized areas of endochondral bone formation (eo) are shown. Quantitative analysis of vascular lumens surrounded by CD31^+^ cells found a reduced density of lumens in the *Postn*HET, *Postn*KO, and *Spp1*KO specimens as compared to WT **(I)**. The reduced lumen density in the *Postn*HET, *Postn*KO, and *Spp1*KO calluses was not associated with changes in mean lumen area **(J)**. The effect of time after fracture, gender, and genotype were determined by 3-way ANOVA and P-values are shown in each graph [insets, **(I, J)]**. Scale bars equal 100 µm.

Neovascularization of fracture calluses collected at 7, 10, and 14 dpf was assessed in WT, *Postn*HET, *Postn*KO, and *Spp1*KO mice by immunohistochemical (IHC) detection of CD31 (Figure 5). CD31 is encoded by *Pecam1* and is expressed by vascular endothelial cells [40]. At 10 and 14 dpf, blood vessels expressing CD31 were abundant in areas of the WT fracture callus that had or were undergoing endochondral ossification but were absent in the cartilage (Figures 5A, B). Blood vessels were similarly abundant in the *Postn*HET fracture calluses at 10 and 14 dpf (Figures 5C, D). However, fewer blood vessels were apparent in the 10 and 14 dpf fracture calluses of the *Postn*KO and *Spp1*KO mice (Figures 5E–H). CD31^+^ lumens in the fracture callus, and therefore presumptive blood vessels, were counted at 7, 10, and 14 dpf for all mouse genotypes and normalized to callus area (Figure 5I). Blood vessel density was dependent upon time after fracture, gender, and genotype. For all genotypes, the number of callus CD31^+^ lumens increased with time after fracture. However, the rate of increase appeared greater in the WT and *Postn*HET calluses, leading to a relative paucity of new blood vessels in the *Postn*KO and *Spp1*KO fracture calluses. The reduced density of CD31^+^ lumens in fracture calluses of male mice was consistent with previously observed sexual dimorphism in vasculogenesis associated with wound healing [41–43]. Quantification of the new blood vessels lumen area found an apparent time-dependent increase in lumen area but no effect of gender or genotype was detected (Figure 5J).

### Effects of *Postn* or *Spp1* disruption on callus macrophages and osteoclasts

**FIGURE 6:**
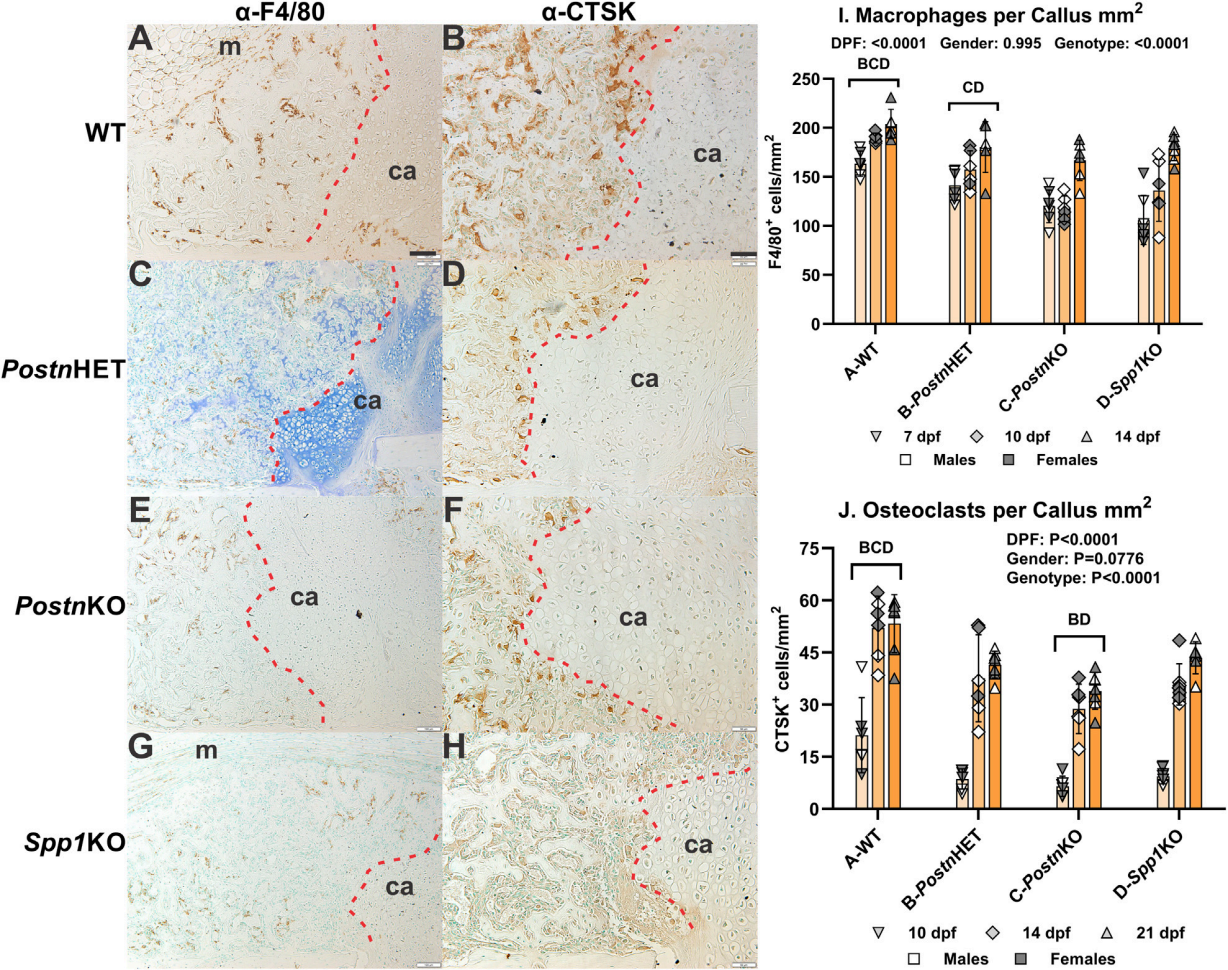
Effects of *Postn* or *Spp1* Deficiency on Callus Macrophages and Osteoclasts. Immunohistochemistry was used to identify macrophages expressing F4/80 [*Adgre1*; **(A, C, E, G)**] or osteoclasts expressing cathepsin K [*Ctsk*; **(B, D, F, H)**] in 7 (not shown), 10 (not shown), 14 (AH), and 21 dpf (not shown) fracture calluses of WT **(A, B)**, *Postn*HET **(C, D)**, *Postn*KO **(E, F)**, and *Spp1*KO mice **(G, H)**. Callus cartilage (ca) and muscle surrounding the callus (m) are noted. The chondro-osseous junction is highlighted with a dashed red line. F4/80 expressing macrophages were detected in areas of endochondral bone formation but were less abundant at the chondro-osseous junction and rare in the callus cartilage. CTSK expressing osteoclasts were also observed in areas of endochondral bone formation, localized at the chondro-osseous junction, and absent from callus cartilage. Quantitative analyses found significant reductions in macrophage **(I)** and osteoclast **(J)** density for all tested genotypes as compared to WT. The effects of time after fracture, gender, and genotype were determined by 3-way ANOVA and Pvalues are shown in each graph [insets, **(I, J)**]. Scale bar equals 100 µm for F4/80 **(A)** and 50 µm for CTSK **(B)** immunostaining.

Fracture callus distribution of macrophages and osteoclasts was determined by immunohistochemical detection of F4/80 and cathepsin K (CTSK), respectively (Figure 6). At 14 dpf, F4/80^+^ cells were detected in calluses from all genotypes (Figures 6A, C, E, G). The F4/80^+^ cells appeared to be absent from callus cartilage but enriched in areas of newly formed bone. Osteoclasts detected by CTSK expression were evident along the callus chondro-osseous junction and within areas of newly formed bone (Figures 6B, D, F, H). Relative to WT mouse calluses, IHC detection of CTSK appeared to be reduced in the *Postn*HET and *Postn*KO mouse calluses and further reduced in the *Spp1*KO mouse callus. Interestingly, F4/80^+^ cells were rarely observed in the region adjacent to the chondro-osseous junction.

The F4/80^+^ and CTSK^+^ cells in fracture calluses collected at 7, 10, and 14 dpf were counted for each mouse genotype and normalized to callus area. The density of F4/80^+^ cells was dependent upon time after fracture and genotype, but not gender (Figure 6I). The density of F4/80^+^ cells increased with time after fracture for all genotypes, but the density of F4/80^+^ cells was reduced in the *Postn*KO and *Spp1*KO mouse calluses. Similarly, the density of CTSK^+^ osteoclasts was dependent upon on time after fracture and genotype but not gender (Figure 6J). The density of osteoclasts increased with time after fracture for all genotypes. Osteoclast density at all time points was highest in the WT mouse calluses and lowest in the *Postn*KO mouse calluses.

### Loss of *Postn* or *Spp1* reduces osteoclast COX-2 expression

**FIGURE 7:**
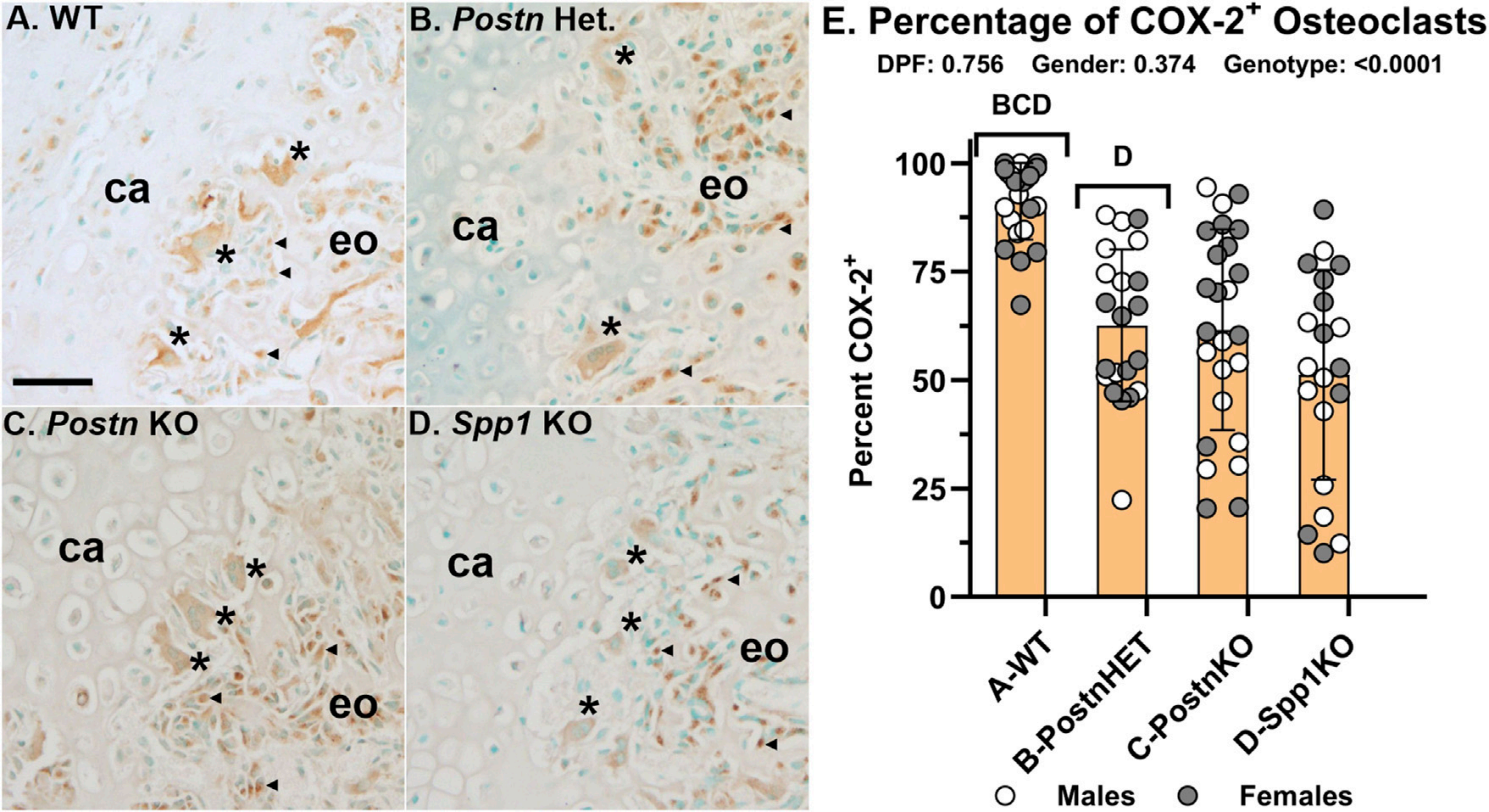
COX-2 Expression is Reduced in Fracture Callus Osteoclasts of *Postn* and *Spp1* Deficient Mice. COX-2 (*Ptgs2*) expression was detected in fracture callus cells by immunohistochemistry. Shown are sections from callus specimens along the chondro-osseous junction collected at 14 dpf for WT **(A)**, *Postn*HET **(B)**, *Postn*KO **(C)**, and *Spp1*KO mice **(D)**. Relative expression of COX-2 in apparent callus osteoblasts (◀) and osteoclasts (*) from WT mice appeared similar **(A)**. In contrast, osteoclast COX-2 expression relative to osteoblast expression appeared lower in the *Postn*HET specimens **(B)**, lower still in the *Postn*KO specimens **(C)**, and weak in the *Spp1*KO specimens **(D)**. The number of osteoclasts that appeared to express COX-2 were compared to the number of TRAP expressing osteoclasts as a percentage **(E)**. Similar to the apparent reduction in osteoclast COX-2 expression, the percentage of COX-2 expressing osteoclasts was reduced in the *Postn* and *Spp1* deficient mouse fracture callus specimens collected at 10, 14, and 21 dpf. The effect of genotype on osteoclast COX-2 expression was significant, but not those of time after fracture or gender as determined by 3-way ANOVA. P-values are shown [inset, **(E)**]. Callus cartilage (ca) and areas of endochondral ossification (eo) are shown. Scale bars equal 20 µm **(A)**.

Fracture callus cells expressing COX-2 were detected by IHC (Figure 7). In 14 dpf WT fracture calluses, COX-2 expression was easily detectable in osteoblasts, some chondrocytes, and osteoclasts, including those at the chondro-osseous junction (Figure 7A). Strong COX-2 expression was evident in osteoblasts and some chondrocytes of the *Postn*HET and *Postn*KO mouse calluses (Figures 7B, C). COX-2 expression was evident but comparatively reduced in osteoclasts of the *Postn*HET and *Postn*KO mouse calluses as compared to the osteoclasts and osteoblasts in the WT mouse calluses and the osteoblasts in the *Postn*HET and *Postn*KO mouse calluses. In the *Spp1*KO mouse calluses, COX-2 expression was evident in osteoblasts and some chondrocytes but osteoclast COX-2 expression was markedly faint in comparison to callus osteoclasts from the WT, *Postn*HET, and *Postn*KO mice (Figure 7D).

Callus osteoclasts and COX-2 expressing osteoclasts were counted at 10, 14, and 21 dpf from WT, *Postn*HET, *Postn*KO, and *Spp1*KO mice (Figure 7E). The percentage of osteoclasts expressing COX-2 was not dependent upon time after fracture or gender. However, the percentage of osteoclasts expressing COX-2 was reduced in the *Postn*HET (63%), *Postn*KO (62%), and *Spp1*KO (51%) calluses as compared to calluses from WT mice (91%).

### Changes in callus mRNA levels associated with loss of *Postn* or *Spp1*

**FIGURE 8:**
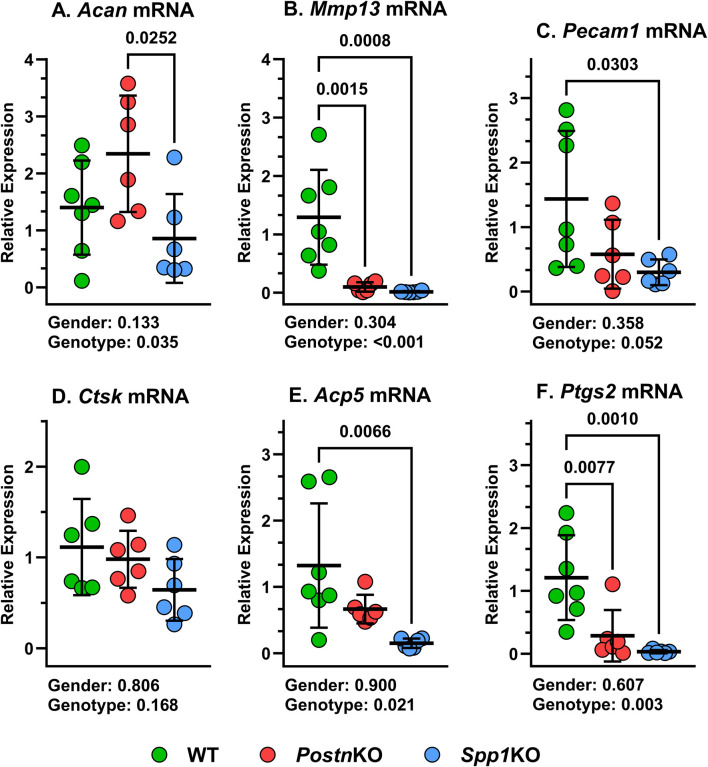
Loss of *Postn* and *Spp1* alters callus gene expression at 14 dpf. RNA was prepared from mouse calluses collected at 14 dpf from at least three male and three female mice of each genotype. mRNA levels for **(A)** *Acan*, **(B)** *MMP13*, **(C)** *Pecam1*, **(D)** *Ctsk*, **(E)** *Acp5*, and **(F)** *Ptgs2* were determined by RTqPCR and normalized to *Actb* (ß-actin) mRNA levels using the 2^−ΔCT^ method. mRNA levels were compared between genotypes and gender using 2-way ANOVA and post-hoc analyses. P values are shown beneath each graph.

Total RNA was isolated from 14 dpf calluses of WT, *Postn*KO, and *Spp1*KO mice and target mRNA levels were measured by RT-qPCR. Chondrogenesis and chondrocyte hypertrophy were assessed by measuring Aggrecan (*Acan*) and MMP-13 (*Mmp13*) mRNA levels, respectively. *Acan* mRNA levels were similar between WT, *Postn*KO and *Spp1*KO mouse calluses, though *Acan* mRNA levels were greater in *Postn*KO calluses than *Spp1*KO calluses (Figure 8A). In contrast and similar to the IHC results (Figure 4), *Mmp13* mRNA levels were significantly reduced in the *Postn*KO and *Spp1*KO mouse calluses as compared to WT (Figure 8B). Vasculogenesis was assessed by measuring CD31 (*Pecam1*) mRNA levels and similar to the IHC results (Figure 5), *Pecam1* mRNA levels were significantly reduced in the *Spp1*KO mice, though not in the *Postn*KO mouse calluses (P = 0.13; Figure 8C). Osteoclast activity was assessed by measuring Cathepsin K (*Ctsk*) and Tartrate Resistant Acid Phosphatase (*Acp5*) mRNA levels. While IHC results showed significant reductions in TRAP^+^ cell density (Figure 3) and CTSK^+^ cell density (Figure 6), only mRNA levels for *Acp5* were significantly lower in the *Spp1*KO mouse calluses (Figures 8D, E). COX-2 (*Ptgs2*) mRNA levels were also measured and were significantly reduced in both the *Postn*KO and *Spp1*KO mouse calluses as compared to WT calluses (Figure 8F).

## Discussion

In control experiments to confirm reduced target mRNA and protein expression in the *Postn*KO and *Spp1*KO mouse calluses (Figure 2), we observed that loss of *Postn* reduced *Spp1* expression and conversely, loss of *Spp1* reduced *Postn* expression. *Postn* and *Spp1* expression were also reduced in the *Postn*HET mouse fracture calluses; consistent with *Postn* affecting *Spp1* expression (Figure 2). Rios et al. also observed reduced POSTN levels in *Postn*HET newborn mouse protein extracts, indicating no compensatory expression from the remaining, normal *Postn* allele [21]. Whether the mutual effects of *Postn* on *Spp1* expression and *Spp1* on *Postn* expression relate to signaling functions associated with these matricellular proteins, a temporal change in fracture healing gene expression associated with impaired healing, or a combination of these effects will require additional investigation. Therefore, ascribing any fracture healing effect to *Postn* versus *Spp1* in this study is limited by the mutual gene expression effects of *Postn* and *Spp1* on each other.

Null mutations in *Postn* or *Spp1* produced similar, negative effects on mouse femur fracture healing. Notably, resolution of callus cartilage by endochondral ossification appeared delayed in the mutant mice as indicated by radiographic imaging, increased callus volume, and reduced callus bone area (Figures 1, 3). The underlying cellular effects of lost *Postn* or *Spp1* in the fracture callus also appeared similar. Callus chondrocyte hypertrophy was abnormal as evidenced by the reduced number and proportion of MMP-13 expressing chondrocytes and *Mmp13* expression in the *Postn*KO and *Spp1*KO mouse calluses (Figures 4, 8). Null mutations in *Postn* or *Spp1* also altered the morphology of the chondro-osseous junction and reduced the overall number of callus osteoclasts (Figures 3, 6, 7). The abnormal morphology of the chondro-osseous junction is particularly evident in the *Postn*HET callus shown in Figures 3C, D in which the transition zone was enlarged between the callus cartilage and the area of new bone formation. The paucity of TRAP^+^ osteoclasts at the chondro-osseous junction was also evident in the *Postn*KO and *Spp1*KO calluses (Figure 3). The apparent altered healing in the *Postn*HET, *Postn*KO, and *Spp1*KO calluses was accompanied by reduced callus vascularization (Figure 5). Thus, *Postn* and *Spp1* appear to have multiple roles during fracture healing including promoting chondrocyte hypertrophy and callus vascularization, which are both necessary for normal fracture healing [8, 10].

In this study, the stabilized femur fractures in the *Postn*KO mice showed delayed healing with apparent bony bridging of the callus by 28 dpf rather than by 21 dpf (Figure 1). The *Postn*KO mouse calluses also had significant reductions in BV/TV, bone area, chondrocyte number, Type X collagen stained area, number of chondrocytes expressing MMP-13, callus vascularity, and osteocalcin expression (Figures 1–5). Similarly, Duchamp de Lageneste et al. found that fracture callus cartilage and bone area were reduced in *Postn*KO mice [20]. However, and in contrast to the stabilized femur fractures used in the present study, the un-stabilized tibia fractures used by Duchamp de Lageneste et al. failed to heal after 28 days in the *Postn*KO mice [20].

Femur fractures in the *Spp1*KO mice also showed delayed healing with apparent bony bridging by 21 dpf as compared to 14 dpf in the wild-type C57BL/6 mice (Figure 1). The *Spp1*KO mouse fracture also had reduced bone area, chondrocytes, TRAP^+^ cells, Type X collagen staining area, MMP-13 expressing chondrocytes, and callus vascularity similar to the *Postn*KO mice (Figures 3–5). Using the same stabilized femur fracture model, Duvall et al. also found that fracture healing was delayed in *Spp1*KO mice and was characterized by reduced callus vascularization at 7 dpf, reduced callus size at 7 and 14 dpf, and reduced callus mechanical properties at 28 dpf, but with a larger callus at 56 dpf. The larger callus in the *Spp1*KO mice at 56 dpf was interpreted as impaired remodeling of the callus though no differences in expression of RANKL, TRAP, Cathepsin K, or OPG were detected in calluses at earlier time points. The current analysis found that callus vascularity was similarly reduced in the *Spp1*KO mice (Figure 5) but, in contrast, that callus osteoclast density was also reduced (Figure 6).

Hypertrophic chondrocytes express Type X Collagen (COL10A1) and MMP-13 [44]. Chondrocyte COL10A1 expression was evident in the fracture calluses of WT, *Postn*HET, *Postn*KO, and *Spp1*KO mice (Figure 4). Indeed, most rounded chondrocytes within the callus were surrounded by COL10A1 suggesting that once differentiated, fracture callus chondrocytes rapidly begin expressing *Col10a1*. In contrast, most, if not all, callus chondrocytes only expressed MMP-13 once the chondrocyte encountered or traversed the chondro-osseous junction. This observation suggests that a signaling event occurs with chondrocytes at the chondro-osseous junction to induce MMP-13 expression (Figure 4C). Furthermore, loss of POSTN or SPP1 appears to impair *Mmp13* induction as evident by the reduced proportion of MMP-13^+^ chondrocytes (Figure 4) and reduced callus *Mmp13* expression (Figure 8), likely contributing to impaired bone formation (Figures 1, 3).

In the WT, *Postn*HET, *Postn*KO, and *Spp1*KO mouse calluses, the chondro-osseous junction is lined with TRAP^+^ and CTSK^+^ osteoclasts (Figures 3, 6). The localization of osteoclasts at the chondro-osseous junction suggests that osteoclasts may mediate the hypertrophic transition from COL10A1^+^, MMP-13^−^ chondrocytes to COL10A1^+^, MMP-13^+^ chondrocytes as the chondro-osseous junction encounters and then traverses callus cartilage. Indeed, the osteoclast secretome has been shown to induce MMP-13 expression in cultured chondrocytes [45]. Culturing osteoclasts on a cartilage substratum or co-culturing osteoclasts with chondrocytes can alter osteoclast activity and promote osteoclast MMP-8 expression [46]. In the present study, loss of *Postn* or *Spp1* reduced callus osteoclast density (Figures 3, 6) as well as the proportion of osteoclasts scored positive for COX-2 expression via IHC from 91% in WT calluses to 62% in *Postn*KO and 51% in *Spp1*KO calluses (Figure 7). Loss of *Postn* or *Spp1* also reduced overall levels of callus COX-2 mRNA (Figure 8). This suggests that as callus chondrocytes encounter the chondro-osseous junction, prostaglandins produced via COX-2 in the chondro-osseous junction osteoclasts help promote *Mmp13* expression in the chondrocytes. Prostaglandins produced via COX-2 can regulate chondrocyte *Mmp13* expression [47]. For instance, prostaglandin E_2_ signaling via the EP2 receptor was reported to inhibit *Mmp13* expression [48], while prostaglandin E_2_ signaling via the EP4 receptor was reported to promote *Mmp13* expression [49]. COX-2 expression is also associated with increased MMP-13 expression in other systems [50, 51]. Determining whether COX-2 expressed in osteoclasts at the chondro-osseous junction is directly or indirectly regulating callus chondrocyte hypertrophy will require additional experimentation.

MMP-13 appears to be a focal point for both osteoclastogenesis and chondrocyte hypertrophy. Treating osteoclast precursors with exogenous MMP-13 promoted osteoclastogenesis *in vitro* independent of MMP-13 proteolytic activity [52]. Hypertrophic chondrocytes expressing *Mmp13* mRNA were found in close proximity to TRAP^+^ osteoclasts in mouse rib fracture calluses [53]. Genetic ablation of *Mmp13* delayed resolution of callus cartilage in mouse tibia fracture calluses and in adolescent mouse growth plates [54, 55]. The expanded hypertrophic zone in the *Mmp13* deficient mouse growth plates was characterized by an increased number of hypertrophic cell layers expressing *Spp1* at the chondro-osseous junction. Similar deficiencies in endochondral ossification between the *Mmp13* deficient mice and the *Postn*HET, *Postn*KO, and *Spp1*KO mice as described here suggests that loss of *Postn* or *Spp1* is affecting callus endochondral ossification, at least in part, through reduced chondrocyte *Mmp13* expression. In support of this conclusion, Xu et al. found that exogenous addition of SPP1, especially phosphorylated SPP1, can induce *Mmp13* expression in cultured human chondrocytes while Attur et al. found that mouse articular chondrocytes lacking *Postn* exhibited reduced *Mmp13* expression in an arthritis model [56, 57].

Neovascularization of the fracture callus is critical for healing and the number of blood vessels (CD31^+^ lumens) was significantly reduced in the *Postn*HET, *Postn*KO, and *Spp1*KO mouse calluses (Figure 5). A prior study also found callus vascularization was deficient in *Spp1* null mice [19]. Loss of *Postn* or *Spp1* reduced callus *Ptgs2* (COX-2) and *Mmp13* mRNA levels (Figure 8) and specifically reduced COX-2 expression in osteoclasts (Figure 7) and MMP-13 expression in chondrocytes (Figure 4). Loss of *Mmp13* impairs growth plate endochondral ossification and chondrocyte MMP-13 expression is associated with angiogenesis during endochondral ossification [7, 55, 58]. COX-2 is also associated with angiogenesis in several systems including tumor growth [59, 60]. COX-2 activity is a rate limiting step in the production of prostaglandins which directly stimulate angiogenesis [61, 62]. For example, prostaglandin E_2_ was found to induce *Mmp13* expression in mouse calvaria cell cultures and promote angiogenesis [63]. Thus, reduced expression of COX-2 in the *Postn*HET, *Postn*KO, and *Spp1*KO calluses could have directly impaired neovascularization of the fracture callus via reduced prostaglandin synthesis (Figure 5) and affected MMP-13 expression in callus chondrocytes (Figures 3, 7).

The decrease in callus osteoclasts in the *Postn* and *Spp1* deficient mice was also accompanied by a reduction in callus F4/80^+^ macrophages (Figure 6). Loss of SPP1 or POSTN could have affected inflammation during the early stages of fracture healing leading to reduced monocyte infiltration of the callus. Unfortunately, inflammation and the early stages of healing were not investigated in the *Postn*HET, *Postn*KO, or *Spp1*KO mice. The available IHC data, however, suggests that the F4/80^+^ macrophages are preferentially located in areas of newly formed bone and bone marrow, scarce at the chondro-osseous junction, and absent from callus cartilage (Figure 6). Thus, the available results indicate that the reduction in F4/80^+^ macrophages is a function of reduced bone and bone marrow formation in the external callus and likely is not causative for the reduction in callus osteoclasts. Still, other macrophage or monocyte populations that do not express F4/80 may have been affected by loss of POSTN or SPP1 leading to the reduction in callus osteoclasts [64].

The mechanisms through which POSTN and SPP1 affect osteoclast COX-2 expression, chondrocyte hypertrophy, and callus neovascularization will require further investigation. As matricellular proteins, POSTN and SPP1 localize in the extracellular matrix to provide signaling cues rather than a structural function within the extracellular matrix [26, 65]. POSTN was widely distributed throughout the fracture callus with apparent greater staining in areas rich with osteoblasts and around chondrocytes at the chondro-osseous junction (Figure 2A). SPP1 also localized at areas rich in osteoblasts and was evident in callus chondrocytes including chondrocytes at the chondro-osseous junction (Figure 2E), consistent with *Spp1* expression in hypertrophic chondrocytes [66]. In the *Postn*HET, and particularly the *Postn*KO fracture calluses, SPP1 expression was reduced but localized to chondrocytes near the chondro-osseous junction (Figures 2F, G).

Both POSTN and SPP1 are integrin ligands and both chondrocytes and osteoclast express integrins, suggesting that local levels of POSTN and SPP1 can alter chondrocyte and osteoclast functions via integrin mediated signaling [31, 32, 34, 67]. For instance, SPP1 signaling via integrin αvß3 can affect osteoclast activity [31, 68, 69]. SPP1 and POSTN also can induce chondrocyte MMP13 expression, though the signaling pathways employed are less clear [56, 70, 71]. In cancer models, SPP1 can induce COX-2 expression, promote matrix metallopeptidase expression, and promote angiogenesis [72, 73]. This suggests that *Spp1* or *Postn* expression in chondrocytes at the chondro-osseous junction may have a similar role by inducing COX-2 expression in osteoclasts, promoting *Mmp13* expression, and promoting angiogenesis. The precise mechanism through which SPP1, POSTN, or other matricellular proteins spatially coordinate events in the fracture callus or at the chondro-osseous junction to control chondrocyte hypertrophy, osteoclast gene expression, and vasculogenesis to enable endochondral bone formation will require a deeper understanding of the cell signaling events involved.

## Data Availability

The original contributions presented in the study are included in the article/Supplementary Material, further inquiries can be directed to the corresponding author.
