# Supplementary material for: Impaired fracture healing is associated with callus chondro-osseous junction abnormalities in periostin-null and osteopontin-null mice
Source: Exp Biol Med (Maywood). 2025 Jan 22;249:10066. doi: 10.3389/ebm.2024.10066 (PMC11794000; doi:10.3389/ebm.2024.10066)
Supplement: Supplementary file 1 [file DataSheet1.PDF]

Supplemental Information

Table of Contents

Figures

Figure S1. Serial X-ray Observations of Femur Fracture Healing in *Postn* and *Spp1* Deficient Mice 3

Figure S2. Calculation of Mouse Femur Fracture External Callus Bone (calcified tissue) Volume and Tissue Volume 4

Methods of S2 µCT Calculations of Murine Femur Fractures 5

Figure S3. Quantification Methods for MMP-13 and CD-31 Immunohistochemical Staining 8

Figure S4. Comparison between Counting Osteoclasts by TRAP Staining vs CTSK IHC 9

Statistical Analyses

Tables S1-A. µCT Analysis: Callus Volume (mm3)10 10

Tables S1-B. µCT Analysis Callus Bone Volume (mm^3) 16

Tables S1-C. µCT Analysis: BV/TV (%) 23

Tables S2-A Postn mRNA Levels (RTqPCR) 30

Tables S2-B Spp1 mRNA Levels (RTqPCR) 31

Tables S2-C B2M mRNA Levels (RTqPCR) 32

Tables S2-D Bglap mRNA Levels (RTqPCR) 34

Tables S3-A. Histomorphometry: Callus Area (mm^2) 37

Tables S3-B. Histomorphometry Callus Percent Bone: (Bone Area/Callus Area) 42

Tables S3-C. Histomorphometry: Callus Percent Cartilage Area: (Cartilage Area/Callus Area) 47

Tables S3-D. Histomorphometry: TRAP+ Cells per mm^2 of Callus (Cells/mm^2) 52

Tables S4-A. Cartilage IHC Callus Percent COL10A1: (COL10A1 area/Callus Area) 57

Tables S4-B. Callus Chondrocytes: (Cell Counts) 62

Tables S4-C. Cartilage IHC Percent $MMP13^+$ Chondrocytes: ($MMP13^+$ Cells/No. of Chondrocytes) 67

Tables S5-A. CD31 IHC Callus Lumen Density: (CD31+ Lumens per callus mm^2) 72

Tables S5-B. CD31 IHC Average Lumen Area: (μm^2) 77

Tables S6-A. F4-80+ Macrophage Density: (F4-80+ cells per callus mm^2) 82

Tables S6-B. CTSK+ Osteoclast Density: (CTSK+ cells per callus mm^2) 87

Tables S7. COX-2 IHC Percent COX-2+ Osteoclasts: (Percent Callus COX-2+ OCs/TRAP+ OCs) 92

Tables S8-A. Relative Aggrecan mRNA Levels (RTqPCR).. 97

Tables S8-B. Relative MMP-13 mRNA Levels (RTqPCR)..99

Tables S8-C. Relative CD31 (Pecam1) mRNA Levels (RTqPCR)..101

Tables S8-D. Relative *Ctsk* mRNA Levels (RTqPCR)..103

Tables S8-E. Relative TRAP (Acp5) mRNA Levels (RTqPCR) 105

Tables S8-F. Relative COX-2 (Ptgs2) mRNA Levels (RTqPCR) 107

Figure S1. Serial X-ray Observations of Femur Fracture Healing in *Postn* and *Spp1* Deficient Mice. Fracture healing was observed in C57BL/6 (WT), *Postn*$^{+/-}$ (*Postn*HET), *Postn* $^{-/-}$ (*Postn*KO), and *Spp1*$^{-/-}$ (*Spp1*KO) mice by X-ray radiography using an XPERT80 digital radiography cabinet (KUBTEC, Startford, CT). The XPERT80 was set on auto-exposure (65 kV, 90 µA, cumulative 8 second exposure). Shown are serial images of femur fracture healing in a WT (A-F), *Postn*HET (G-L), *Postn*KO (M-R), and *Spp1*KO (S-X) mouse at 0 (A, G, M, S), 7 (B, H, N, T), 10, (C, I, O, U), 14, (D, J. P. V), 21 (E, K, Q, W), and 28 (F, L, R, X) dpf.

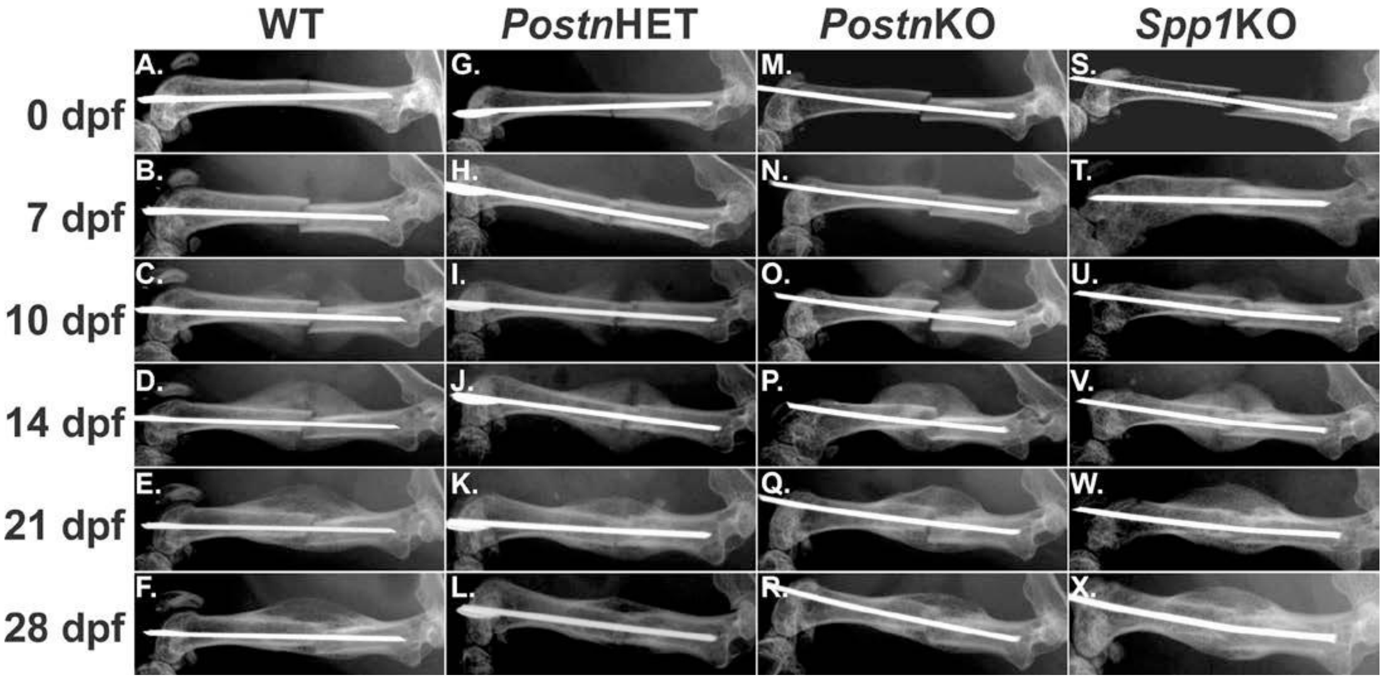

Figure S2. Calculation of Mouse Femur Fracture External Callus Bone (calcified tissue) Volume and Tissue Volume. (A) Representative longitudinal µCT image of 14 days-post-fracture (dpf) fractured femur from a C57BL/5 male mouse is shown in the dorsal-ventral plane with proximal end of femur on top. The proximal and distal callus margins represented by cross-sectional µCT images at the top and bottom of the callus (yellow arrows), were used to define the volume of interest (VOI) for further analysis. (B) Cross-sectional µCT image of the 14 dpf fractured femur taken at the approximate location of the red box shown in panel A. (C) Total bone area from the same µCT section shown in B after grayscale (minimum 75 to maximum 175) adaptive thresholding. (D) The cortical and intramedullary bone area in the same µCT section was identified by tracing the cortical edges. (E) The external callus bone area for the µCT section was defined by subtracting the cortical and intramedullary calcified bone area from the total bone area. (F) Total tissue area for the µCT section was determined by manually tracing the callus periphery. (G) The cortical and intramedullary bone area in the same µCT section was identified by tracing the cortical edges. (H) The external callus tissue area for the µCT section was determined by subtracting the cortical and intramedullary tissue area from the total tissue area. Volumes were determined by the multiplying the voxel dimension (12 µm) by the sum of all individual slices within the volume of interest for external callus bone area (BV) and external callus tissue area (TV). All µCT image analysis and calculations were performed using Bruker Skyscan CTan and Microsoft Excel software.

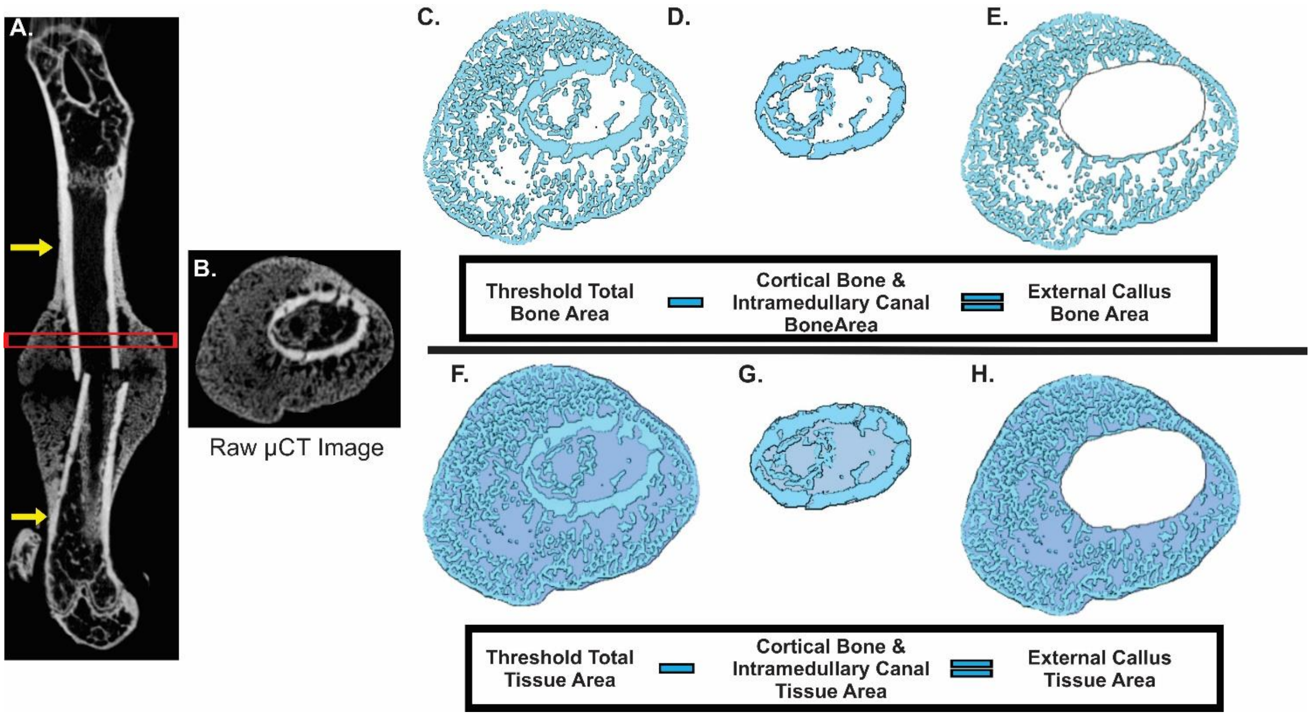

Detailed Methods Description for µCT Analysis of Mouse Femur Fractures

A. µCT Scanning Procedure
Fractured mouse femurs with intramedullary pins removed were scanned in a custom, 3D printed, cylindrical, multi-specimen holder immersed in 70% ethanol using a Bruker Skyscan 1275 system (Micro Photonics Inc., Allentown, PA). Scanning was performed at 73 kVp, with an intensity of 133 µA, a voxel size of 12 µm isotropic, and with a 0.5 mm aluminum filter. Scanner output was reconstructed using NRecon into transverse slices. The 3D reconstructed images of each bone were aligned and separated using DataViewer and then analyzed using CTan. All software was from Bruker (Kontich, Belgium).

B. Defining the Fracture Callus Volume of Interest (VOI)

B.1. Proximal and Distal Margins of the Fracture Callus
For femur fractures the VOI was defined by the proximal and distal margins of the fracture callus that were identified using longitudinal and cross-sectional µCT images (yellow arrows, Figure S2A) at the top and bottom of the callus. The selections were then set in CTan Regions of Interest Preview tab. Only image data between the proximal and distal ends were used for further analysis.

B.2. Circumferential Margins of the Fracture Callus
The outer margins of the fracture callus as viewed in cross-section (CTan Regions of Interest Preview tab) were hand-traced for every 5th cross-section between the proximal and distal margins of the callus. CTan was then interpolated the traced callus peripheral margins to the intervening cross-sections. When tracing was complete, the dataset was saved.

The callus VOI was then determined using one of two methods as detailed below.

B2.a. CTan Automated Analysis Method for Bridged Fractures
For fracture calluses that were well bridged with bone, the following CTan custom task list and parameters were used to define the circumferential margins of the callus. The custom task list defined the ROI for each µCT cross-section. The callus VOI was then calculated based on the voxel dimensions (12 µm), the number of µCT cross-sections within the VOI, and the ROI area for each cross-section. The VOI is equivalent to Callus Total Volume.

1) Thresholding Image: Adaptive (Mean-C), Pre-Threshold, Low (55), High (175), Radius 3 (used to identify and binarize calcified tissue)
2) Despeckle: Remove white speckles of area less than 12 pixels, apply to Image (removes artifacts outside callus perimeter)
3) Morphological Operations: Type Closing, 2D space, Radius (40 -or as small as possible to fill in the ROI view), Apply to Image ROI (fills voids within the callus ROI)
4) Shrink Wrap: Mode Shrink Wrap, 3D Space, Stretch over holes, diameter four voxels (defines the circumferential margins of each cross-sectional image)
5) Save Bitmaps: Apply to ROI, file format BMP, Custom Subfolder Total Bone TV ROI, Copy dataset log file. (This saves the Region of Interest image set)

B.2.b. CTan Automated Analysis Method for Unbridged Fractures
For fractures that were not well bridged with bone, the following CTan custom task list and parameters were used to define the circumferential margins of the callus. The custom task list defined the ROI for each µCT cross-section. The callus VOI was then calculated based on the voxel dimensions (12 µm), the number of µCT cross-sections within the VOI, and the ROI area for each cross-section. The VOI is equivalent to Callus Total Volume.

1) Thresholding Image Adaptive (Mean-C), Pre-Threshold, Low (10), High (20), Radius 1 (used to identify all tissue within with trace margins and binarize)
2) Morphological Operations: Type Closing, 2D space, Kernel Round, Radius (15), Apply to Image (fills voids within the callus ROI)
3) ROI Shrink Wrap: Mode Shrink Wrap, 3D Space, Stretch over holes, diameter four voxels (defines the circumferential margins of each cross-sectional image)
4) Save Bitmaps: Apply to ROI, file format BMP, Custom Subfolder Total Bone TV ROI, Copy dataset log file (this saves the Region of Interest image set)

C. Determining Fracture Callus Bone Volume

Once the ROI/VOI was established for the callus, the original dataset was reloaded and re-thresholded to identify calcified tissue (bone). The resulting images were analyzed within the VOI boundaries to find the Callus Total Bone Volume using the CTan custom task list detailed below.

1) Reload: Apply to Image
2) Thresholding: Adaptive (Mean-C), 2D space, Output to image, Pre-Threshold, Round Kernel, Dark Background, Low (75), High (170), Radius 2, constant zero (used to identify calcified tissue that is presumed to be bone)
3) 3D Analysis: Basic Values, both (text table to stdout, single text line to file) Total Bone Analysis
4) Save Bitmaps: Apply to Image inside ROI, file format BMP, Custom Subfolder Total Bone BV, Copy dataset log file. (this saves the binarized bone images)
5) Bitwise Operations: [Clipboard] = [Image] [XOR] [Region of Interest] (this creates an image set showing the Tissue Volume in white with the Bone Volume superimposed on top in black)
6) Save Bitmaps: Apply to Clipboard, file format BMP, Custom Subfolder Everything Inside Total Bone, Copy dataset log file (this saves images of the Tissue Volume in white with black bone superimposed on top)

D. Determining the Cortical Volume (Femur Cortical Bone and Intramedullary Space)

The peripheral margins of cortical bone were tightly traced every 10th cross-section and intramedullary bone areas were included in this manual delineation. Fragments significantly displaced from most of the cortical bone were considered part of the callus. The Traced images were then used to calculate the cortical bone and intramedullary VOI using a CTan custom task list as follows. The cortical bone VOI was then calculated based on the voxel dimensions (12 µm), the number of µCT cross-sections within the VOI, and the ROI area for each cross-section. The VOI is equivalent to the Cortical Total Volume.

1) Thresholding: Adaptive (Mean-C), Pre-Threshold, Round Kernel, Low (10), High (20), Radius 1 (thresholding set low to fill traced ROI)
2) Morphological Operations: Type Closing, Radius (5) (used to fill any voids within ROI missed after the above thresholding step)
3) ROI Shrink Wrap: Mode Shrink Wrap, 3D Space, Stretch over holes, diameter four voxels (defines circumferential margins of the cortical bone and intramedullary cavity)
4) Save Bitmaps: Apply to ROI, file format BMP, Custom Subfolder CIM TV ROI, Copy dataset log file (this saves the Region of Interest image set)

E. Determining the Cortical and Intramedullary Bone Volume

Once the ROI/VOI was established for the Cortical Total Volume, the original dataset was reloaded and re-thresholded to identify calcified tissue (bone). The resulting images were analyzed within the VOI boundaries to find the Cortical and Intramedullary Bone Volume using the CTan custom task list detailed below.

1) Reload: Apply to Image

2) Thresholding Image: Adaptive (Mean-C), Pre-Threshold, Low (75), High (170), Radius 2, constant zero (thresholding to identify calcified tissue that is presumably bone)
3) Save Bitmaps: Apply to Image, file format BMP, Custom Subfolder: BV CIM Bone, Copy dataset log file (this saves the binarized bone images)
4) Bitwise Operations: [Clipboard] = [Image] [XOR] [Region of Interest] (this creates an image set showing the Tissue Volume in white with the Bone Volume superimposed on top in black)
5) Save Bitmaps: Apply to Clipboard, file format BMP, Custom subfolder: binary bone in CIM VOI, Copy dataset log file. (this saves images of the Tissue Volume in white with black bone superimposed on top)
6) 3D Analysis: Basic Values, both (text table to stdout, single text line to file)

F. Calculations

The External Callus Volume (TV) was calculated by subtracting the Cortical Volume from the Callus Total Volume. The External Callus Bone Volume (BV) was calculated by subtracting the Cortical Bone Volume from the Callus Total Bone Volume.

Figure S3. Quantification Methods for MMP-13 and CD-31 Immunohistochemical Staining. Expression of MMP-13 in hypertrophic chondrocytes at the chondro-osseous junction of a 14 dpf mouse femur fracture callus is shown in **Panel A**. The black box in Panel A corresponds to the image shown in **Panel B**. Expression of MMP-13 around the hypertrophic chondrocytes is detected as brown staining. In Panel B, examples of callus chondrocytes are indicated by a red -. Examples of MMP-13 expressing cells are indicated by a green *. Callus chondrocytes were counted and compared to the number of MMP-13 expressing chondrocytes to determine the percentage of hypertrophic chondrocytes in the callus. Expression of CD31 (*Pecam1*) in a 10 dpf mouse femur fracture is shown in **Panel C**. **Panel D** provides a more detailed image of the presumptive vascular lumens formed by the CD31 expressing cells (stained brown). Lumen peripheries were marked (denoted with the black dotted lines) and used to calculate the number of lumens, individual lumen area, and the percentage of lumen area per callus area. Representative lumens are marked with a green *. Image analysis was performed using OsteoMeasure software (Osteometrics, Inc., Decatur, GA). Scale bars equal 100 μm for Panels A and C and 20 μm for Panels B and D.

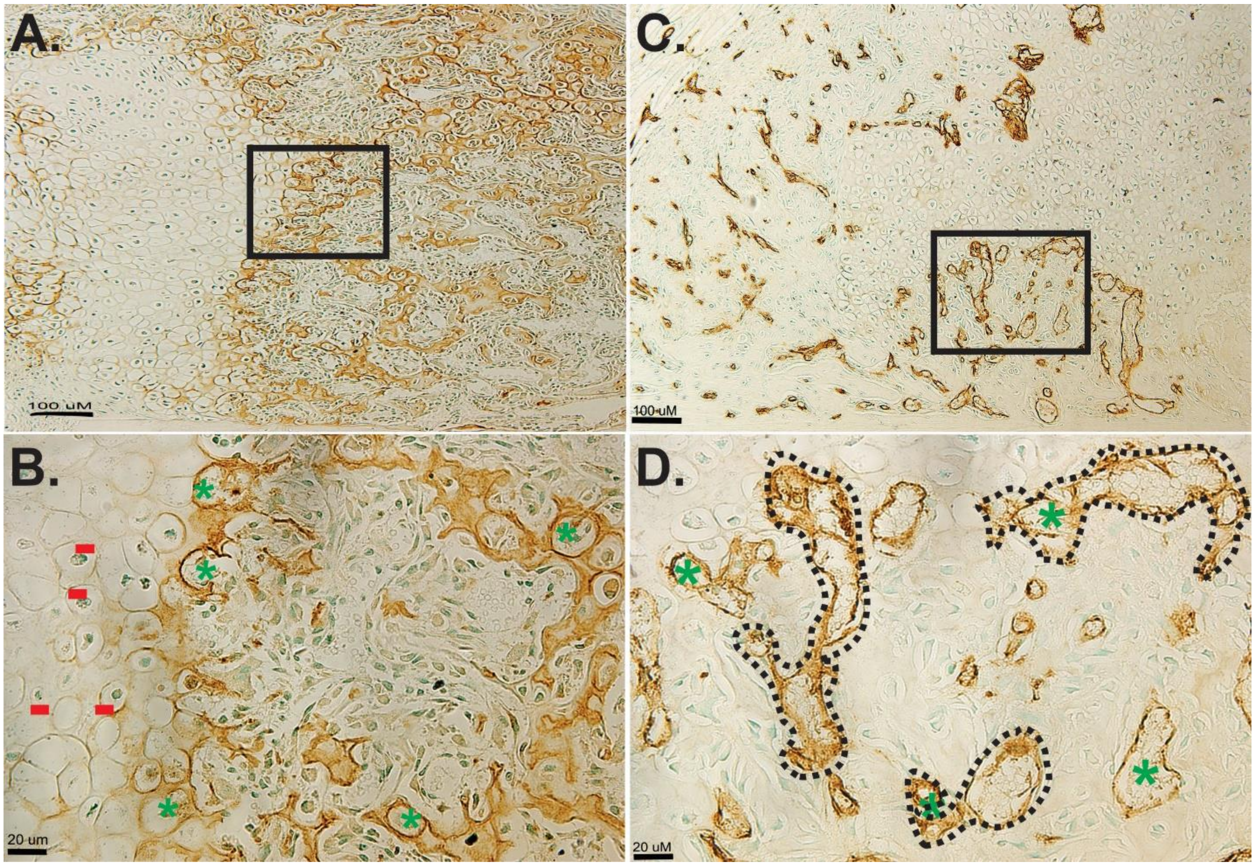

Figure S4. Comparison between Counting Osteoclasts by TRAP Staining vs CTSK IHC. Data were collected at 10, 14, 21 days after fracture (dpf). Only calluses for which osteoclasts were counted by TRAP staining and also by CTSK IHC were used. The data were compared as TRAP+ cells per mm2 of callus versus CTSK+ cells per mm2 of callus. The two methods of detecting osteoclasts were highly correlated (R2=0.91). However, the slope and X-intercept of the best fit-line indicates that TRAP staining detect approximately 15% more positive cells than CTSK IHC (over the range of 100 to 1,000 cells per callus). Based on our observations of the 2 detection methods, we believe that CTSK IHC is better for selectively detecting mature osteoclasts.

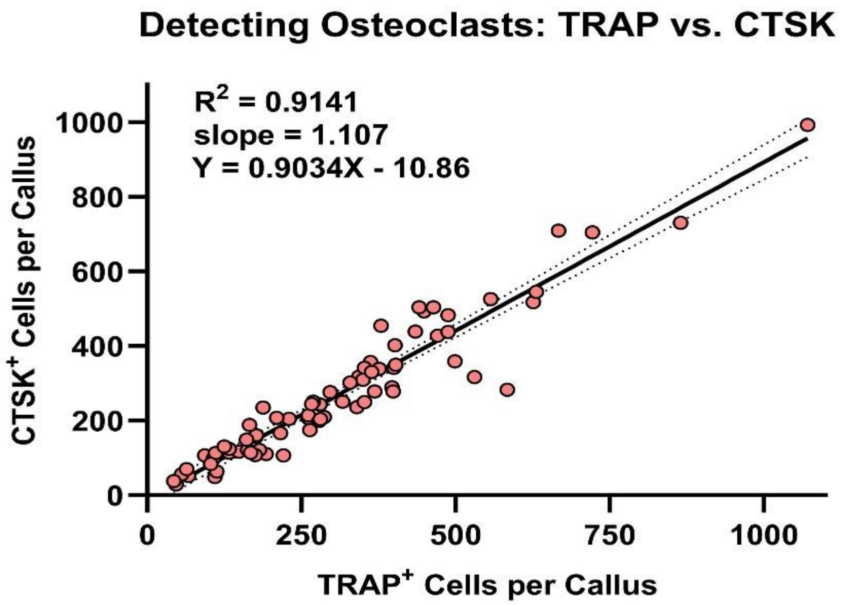

ANOVAThreeWay (10/21/2024 11:50:25)

Table S1-A. µCT Analysis Callus Volume
unit of measure= mm^3

Descriptive Statistics Genotype

	N	Mean	SD	SEM	Variance	Missing	NonMissing
WT	25	22.4044	9.60858	1.92172	92.32485	0	25
PostnHET	20	33.121	24.64051	5.50979	607.15477	0	20
PostnKO	26	25.05272	16.4936	3.23466	272.03876	0	26
Spp1KO	20	34.32706	22.66835	5.0688	513.85387	0	20

Timepoint

	N	Mean	SD	SEM	Variance	Missing	NonMissing
14	30	39.03946	20.23007	3.69349	409.25567	0	30
21	32	27.11465	18.70663	3.3069	349.93806	0	32
28	29	17.98584	10.92798	2.02928	119.42077	0	29

Gender

	N	Mean	SD	SEM	Variance	Missing	NonMissing
Male	45	37.13236	22.04985	3.287	486.19602	0	45
Female	46	19.33665	9.48868	1.39903	90.03503	0	46

*Genotype*Timepoint*

		N	Mean	SD	SEM	Variance	Missing	NonMissing
WT	14	9	30.88876	7.61297	2.53766	57.95727	0	9
	21	7	17.7803	3.86656	1.46142	14.95032	0	7
	28	9	17.51657	9.01307	3.00436	81.23535	0	9
PostnHET	14	7	46.75318	25.33518	9.5758	641.87143	0	7
	21	7	28.09278	26.92133	10.17531	724.75793	0	7
	28	6	23.08304	15.83519	6.46469	250.75315	0	6
PostnKO	14	8	34.49587	17.82829	6.30325	317.84796	0	8
	21	10	26.66862	16.49272	5.21546	272.00986	0	10
	28	8	13.58969	6.94098	2.45401	48.17721	0	8
Spp1KO	14	6	48.32431	26.95096	11.00268	726.35443	0	6
	21	8	34.98387	20.1698	7.1311	406.82097	0	8
	28	6	19.45406	12.46979	5.09077	155.49577	0	6

*Genotype*Gender*

		N	Mean	SD	SEM	Variance	Missing	NonMissing
WT	Male	11	25.08443	10.59729	3.1952	112.3025	0	11
	Female	14	20.29867	8.55609	2.28671	73.20663	0	14
PostnHET	Male	10	46.44687	26.27622	8.30927	690.43971	0	10
	Female	10	19.79513	14.0254	4.43522	196.71193	0	10
PostnKO	Male	13	31.64451	20.13093	5.58331	405.25419	0	13
	Female	13	18.46093	8.20657	2.27609	67.34776	0	13
Spp1KO	Male	11	47.1982	22.85228	6.89022	522.22657	0	11
	Female	9	18.59567	7.83693	2.61231	61.4175	0	9

*Timepoint*Gender*

		N	Mean	SD	SEM	Variance	Missing	NonMissing
14	Male	14	51.18281	23.23043	6.20859	539.6528	0	14
	Female	16	28.41404	8.09149	2.02287	65.47222	0	16
21	Male	16	37.89526	21.34748	5.33687	455.71508	0	16
	Female	16	16.33403	4.42162	1.1054	19.55072	0	16
28	Male	15	23.20485	11.5222	2.97502	132.76115	0	15
	Female	14	12.39404	7.00988	1.87347	49.13843	0	14

Descriptive Statistics
*Genotype*Timepoint*Gender*

			N	Mean	SD	SEM	Variance	Missing	NonMissing
WT	14	Male	4	36.02917	5.65338	2.82669	31.9607	0	4
		Female	5	26.77643	6.66192	2.9793	44.38114	0	5
	21	Male	3	17.45831	1.03312	0.59647	1.06733	0	3
		Female	4	18.02179	5.38588	2.69294	29.00767	0	4
	28	Male	4	19.85926	9.34838	4.67419	87.39212	0	4
		Female	5	15.64242	9.32994	4.17248	87.04785	0	5
PostnHET	14	Male	3	65.21378	27.6406	15.95831	764.00288	0	3
		Female	4	32.90772	13.34234	6.67117	178.01801	0	4
	21	Male	4	40.74561	30.72114	15.36057	943.78867	0	4
		Female	3	11.22233	3.38889	1.95658	11.48459	0	3
	28	Male	3	35.28162	12.12412	6.99986	146.99417	0	3
		Female	3	10.88446	5.78556	3.3403	33.47273	0	3
PostnKO	14	Male	4	40.93141	24.15337	12.07669	583.38547	0	4
		Female	4	28.06033	6.91496	3.45748	47.81665	0	4
	21	Male	4	42.69946	15.1257	7.56285	228.78679	0	4
		Female	6	15.9814	3.11374	1.27118	9.69537	0	6
	28	Male	5	15.37104	8.19407	3.6645	67.14279	0	5
		Female	3	10.62077	3.63041	2.09602	13.17989	0	3
Spp1KO	14	Male	3	71.0252	16.29079	9.40549	265.38987	0	3
		Female	3	25.62342	2.12254	1.22545	4.50516	0	3
	21	Male	5	44.0338	20.9143	9.35316	437.40784	0	5
		Female	3	19.90067	1.7465	1.00834	3.05025	0	3
	28	Male	3	28.6452	8.83086	5.0985	77.98418	0	3
		Female	3	10.26293	7.57127	4.37127	57.32411	0	3

ANOVA

Overall ANOVA

	DF	Sum of Squares	Mean Square	F Value	P Value
Genotype	3	1897.39383	632.46461	3.60954	0.01764
Timepoint	2	7429.52651	3714.76326	21.20051	<0.0001
Gender	1	8071.50214	8071.50214	46.06483	<0.0001
Genotype*Timepoint	6	1249.91303	208.31884	1.1889	0.32295
Genotype*Gender	3	2384.79026	794.93009	4.53674	0.0059
Timepoint*Gender	2	515.48543	257.74271	1.47096	0.23703
Genotype*Timepoint*Gender	6	842.29985	140.38331	0.80118	0.57247
Model	23	20908.19643	909.05202	5.18805	<0.0001
Error	67	11739.77385	175.22051		
Corrected Total	90	32647.97028			

At the 0.05 level, the population means of **Genotype** are **significantly** different. At the 0.05 level, the population means of **Timepoint** are **significantly** different. At the 0.05 level, the population means of **Gender** are **significantly** different.

At the 0.05 level, the population means of **Genotype*Timepoint** are **not significantly** different. At the 0.05 level, the population means of **Genotype*Gender** are **significantly** different.

At the 0.05 level, the population means of **Timepoint*Gender** are **not significantly** different.

At the 0.05 level, the population means of **Genotype*Timepoint*Gender** are **not significantly** different.

Means Comparisons Sidakholm Test

Genotype

	MeanDiff	SEM	t Value	Prob	Alpha	Sig
WT PostnHET	-10.41136	3.44754	-3.01994	0.00358	0.01021	1
WT PostnKO	-3.31284	3.24219	-1.02179	0.31056	0.02532	0
WT Spp1KO	-10.95064	3.46482	-3.16052	0.00236	0.00851	1
PostnHET PostnKO	7.09852	3.43017	2.06944	0.04236	0.01695	0
PostnHET Spp1KO	-0.53928	3.64132	-0.1481	0.88271	0.05	0
PostnKO Spp1KO	-7.6378	3.44754	-2.21544	0.03013	0.01274	0

Timepoint

	MeanDiff	SEM	t Value	Prob	Alpha	Sig
14 21	14.56301	2.94981	4.93694	<0.0001	0.02532	1
14 28	22.49997	3.01737	7.45682	<0.0001	0.01695	1
21 28	7.93696	2.9894	2.65503	0.0099	0.05	1

Gender

	MeanDiff	SEM	t Value	Prob	Alpha	Sig
Male Female	19.28243	2.43778	7.90985	<0.0001	0.05	1

Genotype 's G

	Mean	Groups	
Spp1KO	34.32706	A	
PostnHET	33.121	A	
PostnKO	25.05272	A	B
WT	22.4044		B

Means that do not share a letter are significantly different.

Timepoint 's G

	Mean	Groups		
14	39.03946	A		
21	27.11465		B	
28	17.98584			C

Means that do not share a letter are significantly different.

Gender 's Gro

	Mean	Groups	
Male	37.13236	A	
Female	19.33665		B

Sidakholm Test
Interactions ’s

Genotype	Timepoint	Gender	Mean	Groups			
Spp1KO	14	Male	71.0252	A			
PostnHET	14	Male	65.21378	A	B		
Spp1KO	21	Male	44.0338	A	B	C	
PostnKO	21	Male	42.69946	A	B	C	D
PostnKO	14	Male	40.93141	A	B	C	D
PostnHET	21	Male	40.74561	A	B	C	D
WT	14	Male	36.02917		B	C	D
PostnHET	28	Male	35.28162	A	B	C	D
PostnHET	14	Female	32.90772		B	C	D
Spp1KO	28	Male	28.6452			C	D
PostnKO	14	Female	28.06033			C	D
WT	14	Female	26.77643			C	D
Spp1KO	14	Female	25.62342			C	D
Spp1KO	21	Female	19.90067			C	D
WT	28	Male	19.85926			C	D
WT	21	Female	18.02179			C	D
WT	21	Male	17.45831			C	D
PostnKO	21	Female	15.9814				D
WT	28	Female	15.64242				D
PostnKO	28	Male	15.37104				D
PostnHET	21	Female	11.22233				D
PostnHET	28	Female	10.88446				D
PostnKO	28	Female	10.62077				D
Spp1KO	28	Female	10.26293				D

Means that do not share a letter are significantly different.

Sig equals 1 indicates that the difference of the means is significant at the 0.05 level. Sig equals 0 indicates that the difference of the means is not significant at the 0.05 level.

ANOVAThreeWay (10/21/2024 11:57:50)

Table S1-B. µCT Analysis Callus Bone Volume
unit of measure= mm^3

Descriptive Statistics Genotype

	N	Mean	SD	SEM	Variance	Missing	NonMissing
WT	25	8.59586	2.92022	0.58404	8.52771	0	25
PostnHET	20	9.39922	5.0489	1.12897	25.49143	0	20
PostnKO	26	7.82249	3.47155	0.68083	12.05166	0	26
Spp1KO	20	12.47654	6.88555	1.53966	47.41083	0	20

Timepoint

	N	Mean	SD	SEM	Variance	Missing	NonMissing
14	30	11.15259	3.4839	0.63607	12.13757	0	30
21	32	10.34887	6.08418	1.07554	37.01726	0	32
28	29	6.55362	3.30493	0.61371	10.92254	0	29

Gender

	N	Mean	SD	SEM	Variance	Missing	NonMissing
Male	45	11.46619	5.65538	0.84305	31.98329	0	45
Female	46	7.38735	2.8889	0.42594	8.34572	0	46

*Genotype*Timepoint*

		N	Mean	SD	SEM	Variance	Missing	NonMissing
WT	14	9	11.02094	2.48386	0.82795	6.16954	0	9
	21	7	7.59329	1.14086	0.4312	1.30155	0	7
	28	9	6.95056	2.81004	0.93668	7.89635	0	9
PostnHET	14	7	10.48097	3.06714	1.15927	9.40732	0	7
	21	7	9.8621	7.04726	2.66361	49.66383	0	7
	28	6	7.59716	4.48853	1.83244	20.14693	0	6
PostnKO	14	8	9.7807	3.53797	1.25086	12.51726	0	8
	21	10	8.61405	3.07982	0.97392	9.48527	0	10
	28	8	4.87483	1.76682	0.62466	3.12164	0	8
Spp1KO	14	6	13.96281	4.30846	1.75892	18.56285	0	6
	21	8	15.35445	8.20297	2.90019	67.28876	0	8
	28	6	7.15305	4.18139	1.70704	17.48399	0	6

*Genotype*Gender*

		N	Mean	SD	SEM	Variance	Missing	NonMissing
WT	Male	11	9.42587	3.17043	0.95592	10.05164	0	11
	Female	14	7.94371	2.64017	0.70561	6.97048	0	14
PostnHET	Male	10	12.00687	5.07386	1.6045	25.74408	0	10
	Female	10	6.79157	3.60006	1.13844	12.9604	0	10
PostnKO	Male	13	8.59009	4.23207	1.17376	17.91039	0	13
	Female	13	7.05489	2.43323	0.67486	5.92062	0	13
Spp1KO	Male	11	16.414	6.59232	1.98766	43.45875	0	11
	Female	9	7.66408	3.30229	1.10076	10.90515	0	9

*Timepoint*Gender*

		N	Mean	SD	SEM	Variance	Missing	NonMissing
14	Male	14	12.60029	4.13331	1.10467	17.08427	0	14
	Female	16	9.88585	2.23425	0.55856	4.99189	0	16
21	Male	16	13.62102	7.13498	1.78375	50.90801	0	16
	Female	16	7.07672	1.65916	0.41479	2.75283	0	16
28	Male	15	8.10921	3.38284	0.87345	11.4436	0	15
	Female	14	4.88691	2.32763	0.62209	5.41786	0	14

*Genotype*Timepoint*Gender*

			N	Mean	SD	SEM	Variance	Missing	NonMissing
WT	14	Male	4	12.33899	2.73175	1.36588	7.46248	0	4
		Female	5	9.96651	1.90137	0.85032	3.6152	0	5
	21	Male	3	7.45745	1.26936	0.73287	1.61128	0	3
		Female	4	7.69516	1.22337	0.61168	1.49663	0	4
	28	Male	4	7.98908	2.65078	1.32539	7.02665	0	4
		Female	5	6.11974	2.9294	1.31007	8.58136	0	5
PostnHET	14	Male	3	11.31831	1.94988	1.12576	3.80203	0	3
		Female	4	9.85296	3.87981	1.93991	15.05296	0	4
	21	Male	4	13.4475	7.63696	3.81848	58.32323	0	4
		Female	3	5.08157	1.23132	0.71091	1.51616	0	3
	28	Male	3	10.77459	4.17393	2.40982	17.42173	0	3
		Female	3	4.41972	1.63012	0.94115	2.65731	0	3
PostnKO	14	Male	4	10.01218	5.00232	2.50116	25.02325	0	4
		Female	4	9.54921	2.01017	1.00509	4.0408	0	4
	21	Male	4	11.26193	3.3451	1.67255	11.18971	0	4
		Female	6	6.84879	1.00564	0.41055	1.01132	0	6
	28	Male	5	5.31494	2.00054	0.89467	4.00217	0	5
		Female	3	4.14131	1.27675	0.73713	1.63008	0	3
Spp1KO	14	Male	3	17.68149	1.947	1.1241	3.79083	0	3
		Female	3	10.24413	1.06325	0.61386	1.13049	0	3
	21	Male	5	19.34524	7.93213	3.54736	62.91873	0	5
		Female	3	8.70313	1.87008	1.07969	3.49721	0	3
	28	Male	3	10.26111	2.6435	1.52623	6.98811	0	3
		Female	3	4.04499	2.78241	1.60642	7.7418	0	3

ANOVA

Overall ANOVA

	DF	Sum of Squares	Mean Square	F Value	P Value
Genotype	3	175.70305	58.56768	4.82099	0.00424
Timepoint	2	334.58308	167.29154	13.77058	<0.0001
Gender	1	385.00181	385.00181	31.69138	<0.0001
Genotype*Timepoint	6	90.88252	15.14709	1.24683	0.29401
Genotype*Gender	3	157.90887	52.63629	4.33275	0.0075
Timepoint*Gender	2	31.47462	15.73731	1.29541	0.28055
Genotype*Timepoint*Gender	6	56.11591	9.35265	0.76986	0.59628
Model	23	1347.32006	58.57913	4.82193	<0.0001
Error	67	813.94756	12.14847		
Corrected Total	90	2161.26762			

At the 0.05 level, the population means of **Genotype** are **significantly** different. At the 0.05 level, the population means of **Timepoint** are **significantly** different. At the 0.05 level, the population means of **Gender** are **significantly** different.

At the 0.05 level, the population means of **Genotype*Timepoint** are **not significantly** different. At the 0.05 level, the population means of **Genotype*Gender** are **significantly** different.

At the 0.05 level, the population means of **Timepoint*Gender** are **not significantly** different.

At the 0.05 level, the population means of **Genotype*Timepoint*Gender** are **not significantly** different.

Means Comparisons Sidakholm Test

Genotype

	MeanDiff	SEM	t Value	Prob	Alpha	Sig
WT PostnHET	-0.55462	0.90777	-0.61097	0.54329	0.05	0
WT PostnKO	0.73976	0.8537	0.86653	0.38929	0.02532	0
WT Spp1KO	-3.11886	0.91232	-3.41859	0.00108	0.01021	1
PostnHET PostnKO	1.29438	0.9032	1.43311	0.15648	0.01695	0
PostnHET Spp1KO	-2.56424	0.9588	-2.67443	0.0094	0.01274	1
PostnKO Spp1KO	-3.85862	0.90777	-4.25065	<0.0001	0.00851	1

Timepoint

	MeanDiff	SEM	t Value	Prob	Alpha	Sig
14 21	1.39038	0.77672	1.79007	0.07796	0.05	0
14 28	4.73729	0.7945	5.96257	<0.0001	0.01695	1
21 28	3.34691	0.78714	4.25198	<0.0001	0.02532	1

Gender

	MeanDiff	SEM	t Value	Prob	Alpha	Sig
Male Female	4.2113	0.64189	6.56076	<0.0001	0.05	1

Genotype 's G

	Mean	Groups	
Spp1KO	12.47654	A	
PostnHET	9.39922		B
WT	8.59586		B
PostnKO	7.82249		B

Means that do not share a letter are significantly different.

Timepoint 's G

	Mean	Groups	
14	11.15259	A	
21	10.34887	A	
28	6.55362		B

Means that do not share a letter are significantly different.

Gender 's Gro

	Mean	Groups	
Male	11.46619	A	
Female	7.38735		B

Means that do not share a letter are significantly different.

Interactions 's

Genotype	Timepoint	Gender	Mean	Groups			
Spp1KO	21	Male	19.34524	A			
Spp1KO	14	Male	17.68149	A	B		
PostnHET	21	Male	13.4475	A	B	C	
WT	14	Male	12.33899	A	B	C	D
PostnHET	14	Male	11.31831	A	B	C	D
PostnKO	21	Male	11.26193		B	C	D
PostnHET	28	Male	10.77459		B	C	D
Spp1KO	28	Male	10.26111		B	C	D
Spp1KO	14	Female	10.24413		B	C	D
PostnKO	14	Male	10.01218		B	C	D
WT	14	Female	9.96651		B	C	D
PostnHET	14	Female	9.85296		B	C	D
PostnKO	14	Female	9.54921		B	C	D
Spp1KO	21	Female	8.70313		B	C	D
WT	28	Male	7.98908			C	D
WT	21	Female	7.69516			C	D
WT	21	Male	7.45745			C	D
PostnKO	21	Female	6.84879			C	D
WT	28	Female	6.11974			C	D
PostnKO	28	Male	5.31494				D
PostnHET	21	Female	5.08157			C	D
PostnHET	28	Female	4.41972				D
PostnKO	28	Female	4.14131				D
Spp1KO	28	Female	4.04499				D

Means that do not share a letter are significantly different.

Sig equals 1 indicates that the difference of the means is significant at the 0.05 level. Sig equals 0 indicates that the difference of the means is not significant at the 0.05 level.

ANOVAThreeWay (10/21/2024 12:03:22)

Descriptive Statistics Genotype

Table S1-C. µCT Analysis: BV/TV
unit of measure= percent

	N	Mean	SD	SEM	Variance	Missing	NonMissing
WT	25	40.77895	8.07179	1.61436	65.1538	0	25
PostnHET	20	34.88055	11.5142	2.57465	132.57686	0	20
PostnKO	26	35.12768	8.68478	1.70323	75.42545	0	26
Spp1KO	20	39.17603	7.66332	1.71357	58.7265	0	20

Timepoint

	N	Mean	SD	SEM	Variance	Missing	NonMissing
14	30	31.99613	9.29403	1.69685	86.37893	0	30
21	32	40.9408	7.74813	1.36969	60.03359	0	32
28	29	39.44605	8.30046	1.54136	68.8976	0	29

Gender

	N	Mean	SD	SEM	Variance	Missing	NonMissing
Male	45	34.33491	10.11789	1.50829	102.37161	0	45
Female	46	40.62726	7.1299	1.05125	50.83553	0	46

*Genotype*Timepoint*

		N	Mean	SD	SEM	Variance	Missing	NonMissing
WT	14	9	36.23945	5.11659	1.70553	26.17946	0	9
	21	7	43.43217	6.45931	2.44139	41.72264	0	7
	28	9	43.25483	10.08718	3.36239	101.75117	0	9
PostnHET	14	7	27.70883	13.39461	5.06269	179.41566	0	7
	21	7	40.18095	7.22904	2.73232	52.25905	0	7
	28	6	37.06374	10.42708	4.25684	108.72392	0	6
PostnKO	14	8	30.26396	8.42465	2.97857	70.97481	0	8
	21	10	36.77873	9.3933	2.97042	88.23402	0	10
	28	8	37.92758	6.71336	2.37353	45.06925	0	8
Spp1KO	14	6	32.94257	8.96816	3.66124	80.42785	0	6
	21	8	44.6283	4.97918	1.76041	24.79223	0	8
	28	6	38.1398	3.54179	1.44593	12.54425	0	6

*Genotype*Gender*

		N	Mean	SD	SEM	Variance	Missing	NonMissing
WT	Male	11	39.90043	9.33668	2.81512	87.17365	0	11
	Female	14	41.46922	7.21534	1.92838	52.06111	0	14
PostnHET	Male	10	29.64447	10.16169	3.21341	103.26001	0	10
	Female	10	40.11662	10.75635	3.40146	115.699	0	10
PostnKO	Male	13	30.52437	8.88828	2.46517	79.00158	0	13
	Female	13	39.73098	5.67646	1.57437	32.22222	0	13
Spp1KO	Male	11	37.5368	9.44983	2.84923	89.29927	0	11
	Female	9	41.17953	4.4318	1.47727	19.64086	0	9

*Timepoint*Gender*

		N	Mean	SD	SEM	Variance	Missing	NonMissing
14	Male	14	27.09763	8.76228	2.34182	76.77757	0	14
	Female	16	36.28232	7.64637	1.91159	58.46692	0	16
21	Male	16	37.84454	8.77615	2.19404	77.02078	0	16
	Female	16	44.03706	5.1572	1.2893	26.59669	0	16
28	Male	15	37.34611	9.57879	2.47323	91.7533	0	15
	Female	14	41.69598	6.24851	1.66998	39.04387	0	14

*Genotype*Timepoint*Gender*

			N	Mean	SD	SEM	Variance	Missing	NonMissing
WT	14	Male	4	34.10016	3.97195	1.98597	15.77636	0	4
		Female	5	37.95089	5.68232	2.54121	32.28878	0	5
	21	Male	3	42.69864	6.84222	3.95036	46.81593	0	3
		Female	4	43.98232	7.16192	3.58096	51.29304	0	4
	28	Male	4	43.60204	13.13906	6.56953	172.63501	0	4
		Female	5	42.97706	8.59122	3.84211	73.80909	0	5
PostnHET	14	Male	3	20.31344	10.56162	6.09775	111.54784	0	3
		Female	4	33.25538	13.73883	6.86941	188.75532	0	4
	21	Male	4	35.87255	6.31854	3.15927	39.92397	0	4
		Female	3	45.92549	3.20422	1.84996	10.26701	0	3
	28	Male	3	30.67141	9.13642	5.27491	83.47414	0	3
		Female	3	43.45607	8.10863	4.68152	65.7499	0	3
PostnKO	14	Male	4	26.04918	9.66845	4.83423	93.47894	0	4
		Female	4	34.47873	4.97568	2.48784	24.75738	0	4
	21	Male	4	26.8664	2.97975	1.48987	8.87891	0	4
		Female	6	43.38696	4.74215	1.93598	22.48801	0	6
	28	Male	5	37.03091	8.59706	3.84472	73.90948	0	5
		Female	3	39.42203	2.13619	1.23333	4.5633	0	3
Spp1KO	14	Male	3	25.94306	7.23303	4.17599	52.31673	0	3
		Female	3	39.94207	1.3318	0.76892	1.77369	0	3
	21	Male	5	45.29217	4.17177	1.86567	17.40366	0	5
		Female	3	43.52185	7.00195	4.04258	49.02732	0	3
	28	Male	3	36.20492	2.17364	1.25495	4.72469	0	3
		Female	3	40.07468	3.92488	2.26603	15.40472	0	3

ANOVA

Overall ANOVA

	DF	Sum of Squares	Mean Square	F Value	P Value
Genotype	3	640.12984	213.37661	3.99346	0.01118
Timepoint	2	1475.71613	737.85806	13.80942	<0.0001
Gender	1	1056.85722	1056.85722	19.77966	<0.0001
Genotype*Timepoint	6	291.55109	48.59185	0.90942	0.49365
Genotype*Gender	3	342.37845	114.12615	2.13593	0.10387
Timepoint*Gender	2	98.57214	49.28607	0.92242	0.40255
Genotype*Timepoint*Gender	6	362.31339	60.38556	1.13015	0.35456
Model	23	4112.6836	178.81233	3.34657	<0.0001
Error	67	3579.91143	53.43151		
Corrected Total	90	7692.59503			

At the 0.05 level, the population means of **Genotype** are **significantly** different. At the 0.05 level, the population means of **Timepoint** are **significantly** different. At the 0.05 level, the population means of **Gender** are **significantly** different.

At the 0.05 level, the population means of **Genotype*Timepoint** are **not significantly** different. At the 0.05 level, the population means of **Genotype*Gender** are **not significantly** different.

At the 0.05 level, the population means of **Timepoint*Gender** are **not significantly** different.

At the 0.05 level, the population means of **Genotype*Timepoint*Gender** are **not significantly** different.

Means ComparisonsSidakholm Test

Genotype

	MeanDiff	SEM	t Value	Prob	Alpha	Sig
WT PostnHET	5.96946	1.90377	3.1356	0.00255	0.01021	1
WT PostnKO	6.34615	1.79038	3.54459	7.22318E-4	0.00851	1
WT Spp1KO	2.38873	1.91332	1.24847	0.2162	0.02532	0
PostnHET PostnKO	0.37669	1.89418	0.19886	0.84297	0.05	0
PostnHET Spp1KO	-3.58074	2.01078	-1.78077	0.07948	0.01695	0
PostnKO Spp1KO	-3.95742	1.90377	-2.07873	0.04147	0.01274	0

Timepoint

	MeanDiff	SEM	t Value	Prob	Alpha	Sig
14 21	-9.43918	1.62892	-5.79475	<0.0001	0.01695	1
14 28	-7.67578	1.66623	-4.60668	<0.0001	0.02532	1
21 28	1.76341	1.65079	1.06822	0.28925	0.05	0

Gender

	MeanDiff	SEM	t Value	Prob	Alpha	Sig
Male Female	-6.97739	1.34617	-5.18314	<0.0001	0.05	1

Genotype 's G

	Mean	Groups	
WT	40.77895	A	
Spp1KO	39.17603	A	B
PostnKO	35.12768		B
PostnHET	34.88055		B

Means that do not share a letter are significantly different.

Timepoint 's G

	Mean	Groups	
21	40.9408	A	
28	39.44605	A	
14	31.99613		B

Means that do not share a letter are significantly different.

Gender 's Gro

	Mean	Groups	
Female	40.62726	A	
Male	34.33491		B

Means that do not share a letter are significantly different.

Interactions 's

Genotype	Timepoint	Gender	Mean	Groups				
PostnHET	21	Female	45.92549	A	B			
Spp1KO	21	Male	45.29217	A				
WT	21	Female	43.98232	A	B	C		
WT	28	Male	43.60204	A	B	C		
Spp1KO	21	Female	43.52185	A	B	C	D	
PostnHET	28	Female	43.45607	A	B	C	D	
PostnKO	21	Female	43.38696	A	B			
WT	28	Female	42.97706	A	B	C		
WT	21	Male	42.69864	A	B	C	D	
Spp1KO	28	Female	40.07468	A	B	C	D	E
Spp1KO	14	Female	39.94207	A	B	C	D	E
PostnKO	28	Female	39.42203	A	B	C	D	E
WT	14	Female	37.95089	A	B	C	D	E
PostnKO	28	Male	37.03091	A	B	C	D	E
Spp1KO	28	Male	36.20492	A	B	C	D	E
PostnHET	21	Male	35.87255	A	B	C	D	E
PostnKO	14	Female	34.47873	A	B	C	D	E
WT	14	Male	34.10016	A	B	C	D	E
PostnHET	14	Female	33.25538	A	B	C	D	E
PostnHET	28	Male	30.67141	A	B	C	D	E
PostnKO	21	Male	26.8664			C	D	E
PostnKO	14	Male	26.04918				D	E
Spp1KO	14	Male	25.94306		B	C	D	E
PostnHET	14	Male	20.31344					E

Means that do not share a letter are significantly different.

Sig equals 1 indicates that the difference of the means is significant at the 0.05 level. Sig equals 0 indicates that the difference of the means is not significant at the 0.05 level.

ANOVAOneWay (11/21/2023 11:25:51)

Table S2-A Postn mRNA Levels (RtqPCR) 2^-(Ct-Postn - Ct-B2M)

Descriptive Statistics

	N Analysis	N Missing	Mean	Standard Deviation	SE of Mean
WT	6	0	0.08197	0.04944	0.02018
PostnKO	6	4	0.0074	0.00688	0.00281
Spp1KO	6	4	0.03333	0.02497	0.01019

ANOVA

Overall ANOVA

	DF	Sum of Squares	Mean Square	F Value	Prob>F
Model	2	0.0172	0.0086	8.28092	0.00378
Error	15	0.01557	0.00104		
Total	17	0.03277			

Null Hypothesis: The means of all levels are equal.
Alternative Hypothesis: The means of one or more levels are different.
At the 0.05 level, the population means are significantly different.

Fit Statistics

	R-Square	Coeff Var	Root MSE	Data Mean
	0.52474	0.78783	0.03222	0.0409

Means Comparisons

Tukey Test

	MeanDiff	SEM	q Value	Prob	Alpha	Sig	LCL	UCL
PostnKO WT	-0.07457	0.0186	5.66843	0.00308	0.05	1	-0.12289	-0.02624
Spp1KO WT	-0.04863	0.0186	3.69702	0.04844	0.05	1	-0.09696	-3.11247E-4
Spp1KO PostnKO	0.02593	0.0186	1.97141	0.36873	0.05	0	-0.02239	0.07426

Grouping Letters Table

Tukey Test

	Mean	Groups	
WT	0.08197	A	
Spp1KO	0.03333		B
PostnKO	0.0074		B

Means that do not share a letter are significantly different.

Sig equals 1 indicates that the difference of the means is significant at the 0.05 level.
Sig equals 0 indicates that the difference of the means is not significant at the 0.05 level.

ANOVAOneWay (11/21/2023 11:32:45)

Table S2-B Spp1 mRNA Levels (RtqPCR) 2^-(Ct-Spp1 - Ct-B2M)

Descriptive Statistics

	N Analysis	N Missing	Mean	Standard Deviation	SE of Mean
WT	6	0	9.12367	3.76666	1.53773
PostnKO	6	4	4.59158	2.17291	0.88709
Spp1KO	6	4	0.12128	0.11209	0.04576

ANOVA

Overall ANOVA

	DF	Sum of Squares	Mean Square	F Value	Prob>F
Model	2	243.13253	121.56627	19.27398	7.16328E-5
Error	15	94.6091	6.30727		
Total	17	337.74163			

Null Hypothesis: The means of all levels are equal.
Alternative Hypothesis: The means of one or more levels are different.
At the 0.05 level, the population means are significantly different.

Fit Statistics

	R-Square	Coeff Var	Root MSE	Data Mean
	0.71988	0.54452	2.51143	4.61218

Means Comparisons

Tukey Test

	MeanDiff	SEM	q Value	Prob	Alpha	Sig	LCL	UCL
PostnKO WT	-4.53208	1.44997	4.42031	0.01795	0.05	1	-8.29833	-0.76584
Spp1KO WT	-9.00238	1.44997	8.78036	4.69755E-5	0.05	1	-12.76863	-5.23614
Spp1KO PostnKO	-4.4703	1.44997	4.36005	0.01952	0.05	1	-8.23655	-0.70405

Grouping Letters Table

Tukey Test

	Mean	Groups		
WT	9.12367	A		
PostnKO	4.59158		B	
Spp1KO	0.12128			C

Means that do not share a letter are significantly different.

Sig equals 1 indicates that the difference of the means is significant at the 0.05 level.
Sig equals 0 indicates that the difference of the means is not significant at the 0.05 level.

ANOVATwoWay (10/27/2024 12:44:56

Descriptive Statistics Genotype

Table S2-C B2M mRNA Levels (RtqPCR) 2^-(Ct-B2M - Ct-B2M)

	N	Mean	SD	SEM	Variance	Missing	NonMissing
WT	6	1.00865	0.00769	0.00314	5.91415E-5	0	6
PostnKO	6	1.01247	0.01214	0.00496	1.47473E-4	0	6
Spp1KO	6	1.00624	0.01068	0.00436	1.1408E-4	0	6

Gender

	N	Mean	SD	SEM	Variance	Missing	NonMissing
Male	9	1.00925	0.01099	0.00366	1.20709E-4	0	9
Female	9	1.00899	0.00972	0.00324	9.44687E-5	0	9

Overall

	N	Mean	SD	SEM	Variance	Missing	NonMissing
	18	1.00912	0.01006	0.00237	1.01279E-4	0	18

Interaction

		N	Mean	SD	SEM	Variance	Missing	NonMissing
WT	Male	3	1.00758	0.00574	0.00331	3.2957E-5	0	3
	Female	3	1.00972	0.01056	0.0061	1.11461E-4	0	3
PostnKO	Male	3	1.01856	0.015	0.00866	2.25059E-4	0	3
	Female	3	1.00637	0.00567	0.00327	3.2096E-5	0	3
Spp1KO	Male	3	1.00161	0.00175	0.00101	3.06399E-6	0	3
	Female	3	1.01087	0.01476	0.00852	2.17926E-4	0	3

ANOVA : Overall ANOVA

	DF	Sum of Squares	Mean Square	F Value	P Value
Genotype	2	1.1827E-4	5.91349E-5	0.56992	0.58016
Gender	1	3.21122E-7	3.21122E-7	0.00309	0.95655
Interaction	2	3.58023E-4	1.79011E-4	1.72524	0.2195
Model	5	4.76614E-4	9.53228E-5	0.91868	0.50141
Error	12	0.00125	1.03761E-4		
Corrected Total	17	0.00172			

At the 0.05 level, the population means of **Genotype** are **not significantly** different. At the 0.05 level, the population means of **Gender** are **not significantly** different. At the 0.05 level, the interaction between **Genotype** and **Gender** is **not significant**.

Means Comparisons Tukey Test

Genotype

	MeanDiff	SEM	q Value	Prob	Alpha	Sig	LCL	UCL
PostnKO WT	0.00381	0.00588	0.91657	0.79691	0.05	0	-0.01188	0.0195
Spp1KO WT	-0.00242	0.00588	0.58079	0.91189	0.05	0	-0.01811	0.01327
Spp1KO PostnKO	-0.00623	0.00588	1.49736	0.55606	0.05	0	-0.02192	0.00946

Gender

	MeanDiff	SEM	q Value	Prob	Alpha	Sig	LCL	UCL
Female Male	-2.67134E-4	0.0048	0.07867	0.95655	0.05	0	-0.01073	0.0102

Genotype 's Grouping Letters Table

	Mean	Groups
PostnKO	1.01247	A
WT	1.00865	A
Spp1KO	1.00624	A

Means that do not share a letter are significantly different.

Gender 's Grouping Letters Table

	Mean	Groups
Male	1.00925	A
Female	1.00899	A

Means that do not share a letter are significantly different.

Interactions 's Grouping Letters Table

Genotype	Gender	Mean	Groups
PostnKO	Male	1.01856	A
Spp1KO	Female	1.01087	A
WT	Female	1.00972	A
WT	Male	1.00758	A
PostnKO	Female	1.00637	A
Spp1KO	Male	1.00161	A

Means that do not share a letter are significantly different.

Sig equals 1 indicates that the difference of the means is significant at the 0.05 level. Sig equals 0 indicates that the difference of the means is not significant at the 0.05 level.

ANOVATwoWay (10/27/2024 12:46:5

Table S2-D *Bglap* (OCN) mRNA Levels (RtqPCR) 2^-(Ct-OCN - Ct-B2M)

Descriptive StatisticsGenotype

	N	Mean	SD	SEM	Variance	Missing	NonMissing
WT	6	1.19119	0.58994	0.24084	0.34803	0	6
PostnKO	6	0.33839	0.37028	0.15116	0.1371	0	6
Spp1KO	6	1.3624	0.71065	0.29012	0.50503	0	6

Gender

	N	Mean	SD	SEM	Variance	Missing	NonMissing
Male	9	1.26605	0.79386	0.26462	0.63022	0	9
Female	9	0.66193	0.48438	0.16146	0.23463	0	9

Overall

	N	Mean	SD	SEM	Variance	Missing	NonMissing
	18	0.96399	0.70965	0.16727	0.5036	0	18

Interaction

		N	Mean	SD	SEM	Variance	Missing	NonMissing
WT	Male	3	1.63474	0.38357	0.22145	0.14712	0	3
	Female	3	0.74764	0.36435	0.21036	0.13275	0	3
PostnKO	Male	3	0.54586	0.45285	0.26145	0.20507	0	3
	Female	3	0.13093	0.09255	0.05343	0.00857	0	3
Spp1KO	Male	3	1.61757	1.00071	0.57776	1.00143	0	3
	Female	3	1.10723	0.25651	0.1481	0.0658	0	3

ANOVA: Overall ANOVA

	DF	Sum of Squares	Mean Square	F Value	P Value
Genotype	2	3.61033	1.80516	6.93968	0.00994
Gender	1	1.64235	1.64235	6.31376	0.02727
Interaction	2	0.187	0.0935	0.35944	0.70533
Model	5	5.43968	1.08794	4.1824	0.01967
Error	12	3.12147	0.26012		
Corrected Total	17	8.56115			

At the 0.05 level, the population means of **Genotype** are **significantly** different. At the 0.05 level, the population means of **Gender** are **significantly** different.
At the 0.05 level, the interaction between **Genotype** and **Gender** is **not significant**.

Means ComparisonsTukey Test

Genotype

	MeanDiff	SEM	q Value	Prob	Alpha	Sig	LCL	UCL
PostnKO WT	-0.8528	0.29446	4.09575	0.03337	0.05	1	-1.63838	-0.06722
Spp1KO WT	0.17121	0.29446	0.82226	0.83244	0.05	0	-0.61437	0.95679
Spp1KO PostnKO	1.02401	0.29446	4.91801	0.01176	0.05	1	0.23842	1.80959

Gender

	MeanDiff	SEM	q Value	Prob	Alpha	Sig	LCL	UCL
Female Male	-0.60412	0.24043	3.55352	0.02727	0.05	1	-1.12797	-0.08028

Interactions

Genotype	Gender	Genotype	Gender	MeanDiff	SEM	q Value	Prob	Alpha	Sig	LCL	UCL
WT	Female	WT	Male	-0.8871	0.41643	3.01263	0.33467	0.05	0	-2.28586	0.51166
PostnKO	Male	WT	Male	-1.08888	0.41643	3.69788	0.16681	0.05	0	-2.48764	0.30988
PostnKO	Male	WT	Female	-0.20178	0.41643	0.68526	0.99586	0.05	0	-1.60054	1.19698
PostnKO	Female	WT	Male	-1.50382	0.41643	5.107	0.03271	0.05	1	-2.90257	-0.10506
PostnKO	Female	WT	Female	-0.61671	0.41643	2.09438	0.68157	0.05	0	-2.01547	0.78205
PostnKO	Female	PostnKO	Male	-0.41493	0.41643	1.40912	0.91047	0.05	0	-1.81369	0.98383
Spp1KO	Male	WT	Male	-0.01717	0.41643	0.05832	1	0.05	0	-1.41593	1.38159
Spp1KO	Male	WT	Female	0.86993	0.41643	2.95431	0.35323	0.05	0	-0.52883	2.26869
Spp1KO	Male	PostnKO	Male	1.07171	0.41643	3.63956	0.17765	0.05	0	-0.32705	2.47047
Spp1KO	Male	PostnKO	Female	1.48664	0.41643	5.04868	0.03506	0.05	1	0.08788	2.8854
Spp1KO	Female	WT	Male	-0.52751	0.41643	1.79146	0.79671	0.05	0	-1.92627	0.87124
Spp1KO	Female	WT	Female	0.35959	0.41643	1.22117	0.94837	0.05	0	-1.03917	1.75835
Spp1KO	Female	PostnKO	Male	0.56137	0.41643	1.90643	0.7548	0.05	0	-0.83739	1.96013
Spp1KO	Female	PostnKO	Female	0.9763	0.41643	3.31555	0.24918	0.05	0	-0.42246	2.37506
Spp1KO	Female	Spp1KO	Male	-0.51034	0.41643	1.73314	0.81684	0.05	0	-1.9091	0.88842

Genotype 's Grouping Letters Table

	Mean	Groups	
Spp1KO	1.3624	A	
WT	1.19119	A	
PostnKO	0.33839		B

Means that do not share a letter are significantly different.

Gender 's Grouping Letters Table

	Mean	Groups	
Male	1.26605	A	
Female	0.66193		B

Means that do not share a letter are significantly different.

Interactions 's Grouping Letters Table

Genotype	Gender	Mean	Groups	
WT	Male	1.63474	A	
Spp1KO	Male	1.61757	A	
Spp1KO	Female	1.10723	A	B
WT	Female	0.74764	A	B
PostnKO	Male	0.54586	A	B
PostnKO	Female	0.13093		B

Means that do not share a letter are significantly different.

Sig equals 1 indicates that the difference of the means is significant at the 0.05 level. Sig equals 0 indicates that the difference of the means is not significant at the 0.05 level.

ANOVAThreeWay (7/21/2023 13:41:03)

Descriptive Statistics

Table S3-A. Histomorphometry: Callus Area
unit of measure = mm^2

Days Post-Fracture

	N	Mean	SD	SEM	Variance	Missing	NonMissing
7 dpf	29	7.37764	2.48058	0.46063	6.1533	0	29
10 dpf	38	10.80153	4.99064	0.83177	24.90651	2	36
14 dpf	69	9.97929	3.8367	0.58509	14.7203	26	43
21 dpf	58	7.06414	3.70725	0.60139	13.74367	20	38

Gender

	N	Mean	SD	SEM	Variance	Missing	NonMissing
Female	98	7.38773	3.23328	0.3661	10.45411	20	78
Male	96	10.64869	4.49717	0.54536	20.22455	28	68

Genotype

	N	Mean	SD	SEM	Variance	Missing	NonMissing
WT	50	8.87326	3.95538	0.61033	15.64504	8	42
PostnHet	44	9.66823	4.91992	0.84376	24.20557	10	34
PostnKO	50	8.74796	4.0817	0.67103	16.66024	13	37
Spp1KO	50	8.34188	3.85317	0.67075	14.84694	17	33

*Days Post-Fracture*Gender*

		N	Mean	SD	SEM	Variance	Missing	NonMissing
7 dpf	Female	15	6.66425	1.84468	0.47629	3.40284	0	15
	Male	14	8.14199	2.89349	0.77332	8.37229	0	14
10 dpf	Female	18	9.28332	4.8287	1.17113	23.31638	1	17
	Male	20	12.15994	4.85582	1.114	23.57894	1	19
14 dpf	Female	36	8.07034	2.88621	0.58915	8.33023	12	24
	Male	33	12.39059	3.56469	0.8178	12.707	14	19
21 dpf	Female	29	5.67157	1.50799	0.32151	2.27404	7	22
	Male	29	8.97892	4.89513	1.22378	23.96233	13	16

*Days Post-Fracture*Genotype*

		N	Mean	SD	SEM	Variance	Missing	NonMissing
7 dpf	WT	8	7.27881	2.50119	0.88431	6.25596	0	8
	PostnHet	6	7.82672	3.98719	1.62777	15.89772	0	6
	PostnKO	8	6.89175	1.73781	0.61441	3.02	0	8
	Spp1KO	7	7.66098	2.00554	0.75802	4.02221	0	7
10 dpf	WT	10	10.69991	5.88438	1.8608	34.62595	0	10
	PostnHet	9	11.28478	4.87616	1.62539	23.77695	0	9
	PostnKO	8	12.02511	4.25495	1.50435	18.10464	0	8
	Spp1KO	11	9.34359	5.12144	1.70715	26.22919	2	9
14 dpf	WT	18	9.72921	3.48682	0.96707	12.15789	5	13
	PostnHet	19	11.17795	3.5402	1.06741	12.53303	8	11
	PostnKO	17	9.49528	4.17794	1.32118	17.45521	7	10
	Spp1KO	15	9.41328	4.58376	1.52792	21.01088	6	9
21 dpf	WT	14	7.36071	2.27446	0.68578	5.17318	3	11
	PostnHet	10	7.15488	6.37266	2.25308	40.61085	2	8
	PostnKO	17	7.03518	3.78765	1.14202	14.34629	6	11
	Spp1KO	17	6.60544	1.93905	0.68556	3.75992	9	8

ANOVAThreeWay (7/21/2023 13:41:03)

Descriptive Statistics

*Gender*Genotype*

		N	Mean	SD	SEM	Variance	Missing	NonMissing
Female	WT	25	7.05856	1.52765	0.33336	2.3337	4	21
	PostnHet	24	8.3019	4.43086	0.99077	19.6325	4	20
	PostnKO	25	7.47777	3.39651	0.75948	11.53627	5	20
	Spp1KO	24	6.61294	2.92328	0.709	8.54555	7	17
Male	WT	25	10.68796	4.77734	1.0425	22.82302	4	21
	PostnHet	20	11.62013	5.07702	1.35689	25.77614	6	14
	PostnKO	25	10.24231	4.40418	1.06817	19.39682	8	17
	Spp1KO	26	10.17889	3.94599	0.9865	15.57084	10	16

*Days Post-Fracture*Gender*Genotype*

			N	Mean	SD	SEM	Variance	Missing	NonMissing
7 dpf	Female	WT	4	6.42359	1.41033	0.70517	1.98904	0	4
		PostnHet	3	6.08329	1.58746	0.91652	2.52003	0	3
		PostnKO	4	7.00626	2.30702	1.15351	5.32235	0	4
		Spp1KO	4	6.99864	2.48369	1.24184	6.16871	0	4
	Male	WT	4	8.13404	3.26463	1.63231	10.65778	0	4
		PostnHet	3	9.57015	5.30148	3.06081	28.10565	0	3
		PostnKO	4	6.77724	1.29975	0.64988	1.68935	0	4
		Spp1KO	3	8.54409	0.87542	0.50542	0.76635	0	3
10 dpf	Female	WT	4	8.31078	2.13807	1.06904	4.57136	0	4
		PostnHet	5	11.94388	6.78377	3.0338	46.0196	0	5
		PostnKO	3	10.66659	6.32467	3.65155	40.00143	0	3
		Spp1KO	6	6.57082	1.84132	0.82346	3.39047	1	5
	Male	WT	6	12.29266	7.20925	2.94316	51.9733	0	6
		PostnHet	4	10.4609	0.64551	0.32276	0.41668	0	4
		PostnKO	5	12.84022	3.07697	1.37606	9.46772	0	5
		Spp1KO	5	12.80954	6.04922	3.02461	36.59304	1	4
14 dpf	Female	WT	9	7.22182	0.86956	0.355	0.75613	3	6
		PostnHet	11	9.21905	1.85104	0.69963	3.42636	4	7
		PostnKO	9	8.38493	3.28278	1.34019	10.77661	3	6
		Spp1KO	7	7.10286	4.86166	2.1742	23.63574	2	5
	Male	WT	9	11.87839	3.46553	1.30985	12.00988	2	7
		PostnHet	8	14.60602	3.20959	1.6048	10.30147	4	4
		PostnKO	8	11.1608	5.31415	2.65708	28.24024	4	4
		Spp1KO	8	12.3013	2.12063	1.06032	4.49708	4	4
21 dpf	Female	WT	8	6.56591	1.49824	0.56628	2.24473	1	7
		PostnHet	5	4.70706	1.71403	0.76654	2.93788	0	5
		PostnKO	9	5.60299	1.1832	0.44721	1.39996	2	7
		Spp1KO	7	5.35232	1.3316	0.7688	1.77317	4	3
	Male	WT	6	8.7516	2.94975	1.47488	8.70104	2	4
		PostnHet	5	11.23458	9.81411	5.66618	96.31668	2	3
		PostnKO	8	9.54151	5.64441	2.82221	31.85938	4	4
		Spp1KO	10	7.35732	1.95164	0.8728	3.80889	5	5

ANOVAThreeWay (7/21/2023 13:41:03)

Descriptive Statistics

ANOVA

Overall ANOVA

	DF	Sum of Squares	Mean Square	F Value	P Value
Days Post-Fracture	3	324.40956	108.13652	7.79709	8.82894E-5
Gender	1	333.70893	333.70893	24.0618	3.12649E-6
Genotype	3	31.86436	10.62145	0.76585	0.51547
Days Post-Fracture*Gender	3	38.58272	12.86091	0.92732	0.43003
Days Post-Fracture*Genotype	9	37.17287	4.13032	0.29781	0.97405
Gender*Genotype	3	12.0512	4.01707	0.28965	0.8328
Days Post-Fracture*Gender*Genotype	9	107.39728	11.93303	0.86042	0.56269
Model	31	965.27941	31.13805	2.24518	0.00108
Error	114	1581.04613	13.86883		
Corrected Total	145	2546.32554			

At the 0.05 level, the population means of **Days Post-Fracture** are **significantly** different.
At the 0.05 level, the population means of **Gender** are **significantly** different.
At the 0.05 level, the population means of **Genotype** are **not significantly** different.
At the 0.05 level, the population means of **Days Post-Fracture*Gender** are **not significantly** different.
At the 0.05 level, the population means of **Days Post-Fracture*Genotype** are **not significantly** different.
At the 0.05 level, the population means of **Gender*Genotype** are **not significantly** different.
At the 0.05 level, the population means of **Days Post-Fracture*Gender*Genotype** are **not significantly** different.

ANOVAThreeWay (7/21/2023 13:41:03)

ANOVA

Means Comparisons

Tukey Test

Days Post-Fracture

	MeanDiff	SEM	q Value	Prob	Alpha	Sig	LCL	UCL
7 dpf 10 dpf	-3.29476	0.83291	-5.59424	7.58361E-4	0.05	1	-5.46644	-1.12309
7 dpf 14 dpf	-2.79224	0.80387	-4.91229	0.004	0.05	1	-4.88818	-0.69629
7 dpf 21 dpf	0.053	0.83315	0.08997	0.99991	0.05	0	-2.1193	2.2253
10 dpf 14 dpf	0.50253	0.7606	0.93436	0.91157	0.05	0	-1.48062	2.48567
10 dpf 21 dpf	3.34776	0.79149	5.98168	2.74521E-4	0.05	1	1.28408	5.41145
14 dpf 21 dpf	2.84524	0.76087	5.2884	0.00163	0.05	1	0.8614	4.82908

Gender

	MeanDiff	SEM	q Value	Prob	Alpha	Sig	LCL	UCL
Female Male	-3.13122	0.56406	-7.8506	2.23415E-7	0.05	1	-4.24862	-2.01382

Genotype

	MeanDiff	SEM	q Value	Prob	Alpha	Sig	LCL	UCL
WT PostnHet	-1.03077	0.78715	-1.8519	0.55883	0.05	0	-3.08314	1.0216
WT PostnKO	-0.30022	0.76536	-0.55474	0.97943	0.05	0	-2.29576	1.69532
WT Spp1KO	0.31774	0.78433	0.5729	0.97743	0.05	0	-1.72728	2.36276
PostnHet PostnKO	0.73055	0.81085	1.27416	0.80433	0.05	0	-1.38361	2.84471
PostnHet Spp1KO	1.34851	0.82879	2.30104	0.36762	0.05	0	-0.81242	3.50943
PostnKO Spp1KO	0.61796	0.80811	1.08143	0.87015	0.05	0	-1.48907	2.72498

Days Post-Fracture 's

	Mean	Groups	
10 dpf	10.80153	A	
14 dpf	9.97929	A	
7 dpf	7.37764		B
21 dpf	7.06414		B

Means that do not share a letter are significantly different.

Gender 's Grouping Le

	Mean	Groups	
Male	10.64869	A	
Female	7.38773		B

Means that do not share a letter are significantly different.

Genotype 's Grouping

	Mean	Groups
PostnHet	9.66823	A
WT	8.87326	A
PostnKO	8.74796	A
Spp1KO	8.34188	A

Means that do not share a letter are significantly different.

Sig equals 1 indicates that the difference of the means is significant at the 0.05 level.
Sig equals 0 indicates that the difference of the means is not significant at the 0.05 level.

ANOVAThreeWay (7/21/2023 13:41:03)

ANOVA

Means Comparisons

Tukey Test

Interactions 's Groupin

Days Post-Fracture	Gender	Genotype	Mean	Groups		
14 dpf	Male	PostnHet	14.60602	A		
10 dpf	Male	PostnKO	12.84022	A	B	
10 dpf	Male	Spp1KO	12.80954	A	B	C
14 dpf	Male	Spp1KO	12.3013	A	B	C
10 dpf	Male	WT	12.29266	A	B	C
10 dpf	Female	PostnHet	11.94388	A	B	C
14 dpf	Male	WT	11.87839	A	B	C
21 dpf	Male	PostnHet	11.23458	A	B	C
14 dpf	Male	PostnKO	11.1608	A	B	C
10 dpf	Female	PostnKO	10.66659	A	B	C
10 dpf	Male	PostnHet	10.4609	A	B	C
7 dpf	Male	PostnHet	9.57015	A	B	C
21 dpf	Male	PostnKO	9.54151	A	B	C
14 dpf	Female	PostnHet	9.21905	A	B	C
21 dpf	Male	WT	8.7516	A	B	C
7 dpf	Male	Spp1KO	8.54409	A	B	C
14 dpf	Female	PostnKO	8.38493	A	B	C
10 dpf	Female	WT	8.31078	A	B	C
7 dpf	Male	WT	8.13404	A	B	C
21 dpf	Male	Spp1KO	7.35732	A	B	C
14 dpf	Female	WT	7.22182	A	B	C
14 dpf	Female	Spp1KO	7.10286	A	B	C
7 dpf	Female	PostnKO	7.00626	A	B	C
7 dpf	Female	Spp1KO	6.99864	A	B	C
7 dpf	Male	PostnKO	6.77724	A	B	C
10 dpf	Female	Spp1KO	6.57082	A	B	C
21 dpf	Female	WT	6.56591		B	C
7 dpf	Female	WT	6.42359	A	B	C
7 dpf	Female	PostnHet	6.08329	A	B	C
21 dpf	Female	PostnKO	5.60299		B	C
21 dpf	Female	Spp1KO	5.35232	A	B	C
21 dpf	Female	PostnHet	4.70706			C

Means that do not share a letter are significantly different.

Sig equals 1 indicates that the difference of the means is significant at the 0.05 level.
Sig equals 0 indicates that the difference of the means is not significant at the 0.05 level.

ANOVAThreeWay (7/13/2023 11:42:48)
Descriptive Statistics

Table S3-B. Histomorphometry Callus Percent Bone
unit of measure = percent (bone area/callus area)

Days Post-Fracture

	N	Mean	SD	SEM	Variance	Missing	NonMissing
7 dpf	29	8.43207	3.89876	0.72398	15.20034	0	29
10 dpf	36	17.06029	10.10829	1.70861	102.17751	1	35
14 dpf	42	31.18452	9.74873	1.50426	95.03771	0	42
21 dpf	41	43.31974	8.49164	1.37753	72.10801	3	38

Gender

	N	Mean	SD	SEM	Variance	Missing	NonMissing
Female	78	29.03169	15.55402	1.77255	241.92762	1	77
Male	70	23.31493	15.15692	1.85171	229.73215	3	67

Genotype

	N	Mean	SD	SEM	Variance	Missing	NonMissing
WT	43	28.93786	17.12701	2.64275	293.33442	1	42
PostnHet	34	23.60576	13.66546	2.37885	186.74468	1	33
PostnKO	38	20.96083	14.51378	2.41896	210.64989	2	36
Spp1KO	33	31.77485	14.57519	2.53721	212.43616	0	33

*Days Post-Fracture*Gender*

		N	Mean	SD	SEM	Variance	Missing	NonMissing
7 dpf	Female	15	10.472	4.12494	1.06505	17.01513	0	15
	Male	14	6.24643	2.11405	0.565	4.46919	0	14
10 dpf	Female	17	18.71875	9.53542	2.38386	90.92427	1	16
	Male	19	15.66368	10.61734	2.43579	112.728	0	19
14 dpf	Female	24	32.04458	9.50451	1.9401	90.33573	0	24
	Male	18	30.03778	10.22512	2.41008	104.55301	0	18
21 dpf	Female	22	45.89955	8.04944	1.71615	64.79343	0	22
	Male	19	39.7725	7.99829	1.99957	63.97259	3	16

*Days Post-Fracture*Genotype*

		N	Mean	SD	SEM	Variance	Missing	NonMissing
7 dpf	WT	8	9.2375	3.03504	1.07305	9.21145	0	8
	PostnHet	6	8.16667	2.47444	1.01019	6.12287	0	6
	PostnKO	8	6.155	1.69885	0.60063	2.88609	0	8
	Spp1KO	7	10.34143	6.29418	2.37898	39.61668	0	7
10 dpf	WT	10	13.551	4.46298	1.41132	19.91821	0	10
	PostnHet	9	15.4925	6.64394	2.34899	44.14194	1	8
	PostnKO	8	8.97375	4.22027	1.49209	17.81066	0	8
	Spp1KO	9	29.54111	9.95749	3.31916	99.15169	0	9
14 dpf	WT	13	36.50615	7.34451	2.037	53.94186	0	13
	PostnHet	11	23.95273	6.58864	1.98655	43.41014	0	11
	PostnKO	9	25.03111	8.50156	2.83385	72.27649	0	9
	Spp1KO	9	38.49	7.6842	2.5614	59.047	0	9
21 dpf	WT	12	48.30909	8.89358	2.68152	79.09583	1	11
	PostnHet	8	42.82125	6.43786	2.27613	41.44604	0	8
	PostnKO	13	37.11636	8.52242	2.56961	72.63169	2	11
	Spp1KO	8	45.4875	4.20692	1.48737	17.69814	0	8

ANOVAThreeWay (7/13/2023 11:42:48)

Descriptive Statistics

*Gender*Genotype*

		N	Mean	SD	SEM	Variance	Missing	NonMissing
Female	WT	21	33.37667	17.6291	3.84699	310.78533	0	21
	PostnHet	20	25.82895	14.59989	3.34944	213.15667	1	19
	PostnKO	20	26.0445	15.11211	3.37917	228.37596	0	20
	Spp1KO	17	30.75824	14.08532	3.41619	198.39614	0	17
Male	WT	22	24.49905	15.78524	3.44462	249.17386	1	21
	PostnHet	14	20.58857	12.14544	3.24601	147.51178	0	14
	PostnKO	18	14.60625	11.16733	2.79183	124.70919	2	16
	Spp1KO	16	32.855	15.46476	3.86619	239.15877	0	16

*Days Post-Fracture*Gender*Genotype*

			N	Mean	SD	SEM	Variance	Missing	NonMissing
7 dpf	Female	WT	4	11.7575	0.94256	0.47128	0.88843	0	4
		PostnHet	3	8.52667	2.40612	1.38918	5.78943	0	3
		PostnKO	4	7.575	0.89519	0.4476	0.80137	0	4
		Spp1KO	4	13.5425	6.64124	3.32062	44.10609	0	4
	Male	WT	4	6.7175	1.91587	0.95794	3.67056	0	4
		PostnHet	3	7.80667	3.02141	1.74441	9.12893	0	3
		PostnKO	4	4.735	0.7455	0.37275	0.55577	0	4
		Spp1KO	3	6.07333	2.20731	1.27439	4.87223	0	3
10 dpf	Female	WT	4	15.39	3.27638	1.63819	10.73467	0	4
		PostnHet	5	15.0475	8.67848	4.33924	75.31596	1	4
		PostnKO	3	11.95	5.79697	3.34688	33.6049	0	3
		Spp1KO	5	28.38	9.1668	4.09952	84.03025	0	5
	Male	WT	6	12.325	4.99027	2.03727	24.90275	0	6
		PostnHet	4	15.9375	5.21093	2.60547	27.15382	0	4
		PostnKO	5	7.188	1.93301	0.86447	3.73652	0	5
		Spp1KO	4	30.9925	12.13707	6.06854	147.30849	0	4
14 dpf	Female	WT	6	40.185	7.96592	3.25207	63.45587	0	6
		PostnHet	7	25.35143	5.2714	1.9924	27.78765	0	7
		PostnKO	6	27.865	7.81598	3.19086	61.08959	0	6
		Spp1KO	5	36.662	9.5612	4.2759	91.41647	0	5
	Male	WT	7	33.35286	5.46529	2.06569	29.86942	0	7
		PostnHet	4	21.505	8.75053	4.37527	76.57183	0	4
		PostnKO	3	19.36333	8.00647	4.62254	64.10363	0	3
		Spp1KO	4	40.775	4.79991	2.39995	23.0391	0	4
21 dpf	Female	WT	7	50.17286	8.63152	3.26241	74.50322	0	7
		PostnHet	5	45.504	5.75255	2.57262	33.09188	0	5
		PostnKO	7	41.07857	8.18106	3.09215	66.92975	0	7
		Spp1KO	3	47.83667	6.21032	3.58553	38.56813	0	3
	Male	WT	5	45.0475	9.61027	4.80513	92.35723	1	4
		PostnHet	3	38.35	5.55845	3.20917	30.8964	0	3
		PostnKO	6	30.1825	2.74049	1.37025	7.51029	2	4
		Spp1KO	5	44.078	2.25064	1.00652	5.06537	0	5

ANOVAThreeWay (7/13/2023 11:42:48)

ANOVA

Overall ANOVA

	DF	Sum of Squares	Mean Square	F Value	P Value
Days Post-Fracture	3	22055.17222	7351.72407	174.62856	4.52027E-42
Gender	1	508.59009	508.59009	12.08075	7.2583E-4
Genotype	3	2823.42801	941.14267	22.35535	2.04117E-11
Days Post-Fracture*Gender	3	136.53094	45.51031	1.08103	0.3602
Days Post-Fracture*Genotype	9	1600.54071	177.83786	4.22426	9.98834E-5
Gender*Genotype	3	151.32906	50.44302	1.19819	0.31385
Days Post-Fracture*Gender*Genotype	9	219.17907	24.35323	0.57847	0.81236
Model	31	30004.56553	967.88921	22.99067	1.47367E-35
Error	112	4715.1114	42.09921		
Corrected Total	143	34719.67693			

At the 0.05 level, the population means of **Days Post-Fracture** are **significantly** different.
At the 0.05 level, the population means of **Gender** are **significantly** different.
At the 0.05 level, the population means of **Genotype** are **significantly** different.
At the 0.05 level, the population means of **Days Post-Fracture*Gender** are **not significantly** different.
At the 0.05 level, the population means of **Days Post-Fracture*Genotype** are **significantly** different.
At the 0.05 level, the population means of **Gender*Genotype** are **not significantly** different.
At the 0.05 level, the population means of **Days Post-Fracture*Gender*Genotype** are **not significantly** different.

ANOVAThreeWay (7/13/2023 11:42:48)

ANOVA

Means Comparisons

Tukey Test

Days Post-Fracture

	MeanDiff	SEM	q Value	Prob	Alpha	Sig	LCL	UCL
7 dpf 10 dpf	-8.80954	1.45713	-8.55007	1.13085E-7	0.05	1	-12.60978	-5.00931
7 dpf 14 dpf	-22.29068	1.41299	-22.30996	0	0.05	1	-25.9758	-18.60556
7 dpf 21 dpf	-34.43949	1.44875	-33.61855	0	0.05	1	-38.21786	-30.66112
10 dpf 14 dpf	-13.48114	1.34814	-14.14188	0	0.05	1	-16.99712	-9.96516
10 dpf 21 dpf	-25.62995	1.38557	-26.15981	0	0.05	1	-29.24355	-22.01635
14 dpf 21 dpf	-12.14881	1.33907	-12.83053	0	0.05	1	-15.64114	-8.65648

Gender

	MeanDiff	SEM	q Value	Prob	Alpha	Sig	LCL	UCL
Female Male	3.89969	0.98949	5.57358	1.41575E-4	0.05	1	1.93914	5.86023

Genotype

	MeanDiff	SEM	q Value	Prob	Alpha	Sig	LCL	UCL
WT PostnHet	4.61493	1.37808	4.73595	0.00601	0.05	1	1.02088	8.20899
WT PostnKO	8.12635	1.34678	8.53322	1.19971E-7	0.05	1	4.61391	11.63879
WT Spp1KO	-4.17397	1.36386	-4.32808	0.0145	0.05	1	-7.73095	-0.61699
PostnHet PostnKO	3.51142	1.43396	3.46307	0.07393	0.05	0	-0.22838	7.25122
PostnHet Spp1KO	-8.7889	1.45001	-8.57194	1.0468E-7	0.05	1	-12.57056	-5.00725
PostnKO Spp1KO	-12.30032	1.4203	-12.24761	0	0.05	1	-16.0045	-8.59615

Days Post-Fracture 's

	Mean	Groups			
21 dpf	43.31974	A			
14 dpf	31.18452		B		
10 dpf	17.06029			C	
7 dpf	8.43207				D

Means that do not share a letter are significantly different.

Gender 's Grouping Le

	Mean	Groups	
Female	29.03169	A	
Male	23.31493		B

Means that do not share a letter are significantly different.

Genotype 's Grouping

	Mean	Groups		
Spp1KO	31.77485	A		
WT	28.93786		B	
PostnHet	23.60576			C
PostnKO	20.96083			C

Means that do not share a letter are significantly different.

Sig equals 1 indicates that the difference of the means is significant at the 0.05 level.
Sig equals 0 indicates that the difference of the means is not significant at the 0.05 level.

ANOVAThreeWay (7/13/2023 11:42:48)

ANOVA

Means Comparisons

Tukey Test

Interactions 's Groupin

Days Post-Fracture	Gender	Genotype	Mean	Groups													
21 dpf	Female	WT	50.17286	A													
21 dpf	Female	Spp1KO	47.83667	A	B												
21 dpf	Female	PostnHet	45.504	A	B												
21 dpf	Male	WT	45.0475	A	B	C											
21 dpf	Male	Spp1KO	44.078	A	B	C											
21 dpf	Female	PostnKO	41.07857	A	B	C	D										
14 dpf	Male	Spp1KO	40.775	A	B	C	D	E									
14 dpf	Female	WT	40.185	A	B	C	D	E									
21 dpf	Male	PostnHet	38.35	A	B	C	D	E	F	G							
14 dpf	Female	Spp1KO	36.662		B	C	D	E	F								
14 dpf	Male	WT	33.35286		B	C	D	E	F	G	H						
10 dpf	Male	Spp1KO	30.9925		B	C	D	E	F	G	H	I					
21 dpf	Male	PostnKO	30.1825			C	D	E	F	G	H	I	J				
10 dpf	Female	Spp1KO	28.38				D	E	F	G	H	I	J	K			
14 dpf	Female	PostnKO	27.865					E	F	G	H	I	J	K			
14 dpf	Female	PostnHet	25.35143						F	G	H	I	J	K	L		
14 dpf	Male	PostnHet	21.505							G	H	I	J	K	L	M	
14 dpf	Male	PostnKO	19.36333								H	I	J	K	L	M	N
10 dpf	Male	PostnHet	15.9375									I	J	K	L	M	N
10 dpf	Female	WT	15.39									I	J	K	L	M	N
10 dpf	Female	PostnHet	15.0475										J	K	L	M	N
7 dpf	Female	Spp1KO	13.5425											K	L	M	N
10 dpf	Male	WT	12.325													M	N
10 dpf	Female	PostnKO	11.95												L	M	N
7 dpf	Female	WT	11.7575												L	M	N
7 dpf	Female	PostnHet	8.52667													M	N
7 dpf	Male	PostnHet	7.80667													M	N
7 dpf	Female	PostnKO	7.575													M	N
10 dpf	Male	PostnKO	7.188													M	N
7 dpf	Male	WT	6.7175													M	N
7 dpf	Male	Spp1KO	6.07333													M	N
7 dpf	Male	PostnKO	4.735														N

Means that do not share a letter are significantly different.

Sig equals 1 indicates that the difference of the means is significant at the 0.05 level.
Sig equals 0 indicates that the difference of the means is not significant at the 0.05 level.

ANOVAThreeWay (7/24/2023 14:41:56)
Descriptive Statistics

Table S3-C. Histomorphometry: Callus Percent Cartilage Area
unit of measure = percent (Cartilage Area/Callus Area)

Days Post-Fracture

	N	Mean	SD	SEM	Variance	Missing	NonMissing
7 dpf	29	18.87724	9.8254	1.82453	96.53841	0	29
10 dpf	38	30.55722	10.04396	1.67399	100.8812	2	36
14 dpf	69	20.04093	9.74469	1.48605	94.95904	26	43
21 dpf	58	2.50324	1.95797	0.33579	3.83366	24	34

Gender

	N	Mean	SD	SEM	Variance	Missing	NonMissing
Female	98	17.73467	14.02121	1.61903	196.59436	23	75
Male	96	18.8697	12.17791	1.48777	148.30159	29	67

Genotype

	N	Mean	SD	SEM	Variance	Missing	NonMissing
WT	50	17.8045	12.22756	1.93335	149.51333	10	40
PostnHet	44	20.575	14.05576	2.41054	197.56444	10	34
PostnKO	50	18.35257	14.38716	2.43187	206.9905	15	35
Spp1KO	50	16.37273	12.09961	2.10627	146.40051	17	33

*Days Post-Fracture*Gender*

		N	Mean	SD	SEM	Variance	Missing	NonMissing
7 dpf	Female	15	20.632	12.13016	3.13199	147.14067	0	15
	Male	14	16.99714	6.48923	1.73432	42.11011	0	14
10 dpf	Female	18	33.23118	10.0875	2.44658	101.75761	1	17
	Male	20	28.16474	9.63908	2.21136	92.91188	1	19
14 dpf	Female	36	17.43292	8.99252	1.83559	80.8654	12	24
	Male	33	23.33526	9.88527	2.26784	97.71856	14	19
21 dpf	Female	29	1.96316	1.87681	0.43057	3.52242	10	19
	Male	29	3.18733	1.9001	0.4906	3.61038	14	15

*Days Post-Fracture*Genotype*

		N	Mean	SD	SEM	Variance	Missing	NonMissing
7 dpf	WT	8	23.9675	7.84116	2.77227	61.48374	0	8
	PostnHet	6	18.07167	5.40887	2.20816	29.2559	0	6
	PostnKO	8	12.1475	4.33015	1.53094	18.75016	0	8
	Spp1KO	7	21.44143	15.14177	5.72305	229.27305	0	7
10 dpf	WT	10	31.375	8.09026	2.55836	65.45225	0	10
	PostnHet	9	34.58333	7.73683	2.57894	59.8585	0	9
	PostnKO	8	31.02375	14.78637	5.22777	218.63686	0	8
	Spp1KO	11	25.20778	8.06677	2.68892	65.07272	2	9
14 dpf	WT	18	14.10615	5.5093	1.528	30.35236	5	13
	PostnHet	19	24.12	10.60215	3.19667	112.40552	8	11
	PostnKO	17	26.872	10.21229	3.22941	104.2908	7	10
	Spp1KO	15	16.03778	6.22103	2.07368	38.70119	6	9
21 dpf	WT	14	2.59	2.25693	0.75231	5.09375	5	9
	PostnHet	10	1.81875	1.41978	0.50197	2.01578	2	8
	PostnKO	17	3.13889	2.11971	0.70657	4.49316	8	9
	Spp1KO	17	2.375	2.00432	0.70863	4.01729	9	8

ANOVAThreeWay (7/24/2023 14:41:56)

Descriptive Statistics

*Gender*Genotype*

		N	Mean	SD	SEM	Variance	Missing	NonMissing
Female	WT	25	15.7255	12.97483	2.90126	168.34631	5	20
	PostnHet	24	19.844	14.59055	3.26255	212.88428	4	20
	PostnKO	25	17.76333	15.15276	3.57154	229.60604	7	18
	Spp1KO	24	17.58647	14.21089	3.44665	201.94936	7	17
Male	WT	25	19.8835	11.37761	2.54411	129.45002	5	20
	PostnHet	20	21.61929	13.72496	3.66815	188.37465	6	14
	PostnKO	25	18.97647	13.96761	3.38764	195.09421	8	17
	Spp1KO	26	15.08313	9.66772	2.41693	93.4649	10	16

*Days Post-Fracture*Gender*Genotype*

			N	Mean	SD	SEM	Variance	Missing	NonMissing
7 dpf	Female	WT	4	26.41	10.53134	5.26567	110.90907	0	4
		PostnHet	3	17.50333	5.66369	3.26994	32.07743	0	3
		PostnKO	4	11.6625	2.30814	1.15407	5.32749	0	4
		Spp1KO	4	26.17	18.61641	9.30821	346.57087	0	4
	Male	WT	4	21.525	4.07973	2.03986	16.64417	0	4
		PostnHet	3	18.64	6.33193	3.65574	40.0933	0	3
		PostnKO	4	12.6325	6.14781	3.07391	37.79563	0	4
		Spp1KO	3	15.13667	7.97615	4.60503	63.61903	0	3
10 dpf	Female	WT	4	30.715	9.97542	4.98771	99.50897	0	4
		PostnHet	5	35.948	5.21685	2.33305	27.21557	0	5
		PostnKO	3	44.59333	3.40239	1.96437	11.57623	0	3
		Spp1KO	6	25.71	10.78441	4.82293	116.3034	1	5
	Male	WT	6	31.815	7.58471	3.09645	57.52787	0	6
		PostnHet	4	32.8775	10.78664	5.39332	116.35156	0	4
		PostnKO	5	22.882	12.48463	5.5833	155.86607	0	5
		Spp1KO	5	24.58	4.18452	2.09226	17.5102	1	4
14 dpf	Female	WT	9	11.43667	4.0078	1.63618	16.06247	3	6
		PostnHet	11	22.55429	11.46897	4.33486	131.5372	4	7
		PostnKO	9	21.30167	7.44962	3.04129	55.49678	3	6
		Spp1KO	7	12.816	5.41232	2.42046	29.29318	2	5
	Male	WT	9	16.39429	5.83824	2.20665	34.08503	2	7
		PostnHet	8	26.86	9.79186	4.89593	95.88047	4	4
		PostnKO	8	35.2275	8.0768	4.0384	65.23469	4	4
		Spp1KO	8	20.065	5.02207	2.51103	25.22117	4	4
21 dpf	Female	WT	8	2.89833	2.60987	1.06547	6.81142	2	6
		PostnHet	5	1.35	1.40277	0.62734	1.96775	0	5
		PostnKO	9	2.3	1.37515	0.61499	1.89105	4	5
		Spp1KO	7	0.55333	0.35642	0.20578	0.12703	4	3
	Male	WT	6	1.97333	1.57824	0.9112	2.49083	3	3
		PostnHet	5	2.6	1.28643	0.74272	1.6549	2	3
		PostnKO	8	4.1875	2.61177	1.30589	6.82136	4	4
		Spp1KO	10	3.468	1.72759	0.7726	2.98457	5	5

ANOVAThreeWay (7/24/2023 14:41:56)

ANOVA

Overall ANOVA

	DF	Sum of Squares	Mean Square	F Value	P Value
Days Post-Fracture	3	13954.0629	4651.3543	83.19608	3.59157E-28
Gender	1	1.21685	1.21685	0.02177	0.88298
Genotype	3	271.56303	90.52101	1.6191	0.18907
Days Post-Fracture*Gender	3	1018.36453	339.45484	6.07163	7.30437E-4
Days Post-Fracture*Genotype	9	2094.93153	232.77017	4.16343	1.21331E-4
Gender*Genotype	3	19.88948	6.62983	0.11858	0.94902
Days Post-Fracture*Gender*Genotype	9	982.7643	109.19603	1.95313	0.05168
Model	31	18231.56033	588.11485	10.51927	6.83923E-21
Error	110	6149.91676	55.90833		
Corrected Total	141	24381.47709			

At the 0.05 level, the population means of **Days Post-Fracture** are **significantly** different.
At the 0.05 level, the population means of **Gender** are **not significantly** different.
At the 0.05 level, the population means of **Genotype** are **not significantly** different.
At the 0.05 level, the population means of **Days Post-Fracture*Gender** are **significantly** different.
At the 0.05 level, the population means of **Days Post-Fracture*Genotype** are **significantly** different.
At the 0.05 level, the population means of **Gender*Genotype** are **not significantly** different.
At the 0.05 level, the population means of **Days Post-Fracture*Gender*Genotype** are **not significantly** different.

ANOVAThreeWay (7/24/2023 14:41:56)

ANOVA

Means Comparisons

Tukey Test

Days Post-Fracture

	MeanDiff	SEM	q Value	Prob	Alpha	Sig	LCL	UCL
7 dpf 10 dpf	-12.4301	1.66568	-10.55352	0	0.05	1	-16.77544	-8.08477
7 dpf 14 dpf	-2.12193	1.6076	-1.86667	0.5523	0.05	0	-6.31574	2.07189
7 dpf 21 dpf	16.29369	1.6992	13.56093	0	0.05	1	11.86091	20.72646
10 dpf 14 dpf	10.30818	1.52108	9.58394	0	0.05	1	6.34006	14.27629
10 dpf 21 dpf	28.72379	1.61759	25.11238	0	0.05	1	24.50391	32.94367
14 dpf 21 dpf	18.41561	1.55772	16.7191	5.96925E-8	0.05	1	14.35193	22.47929

Gender

	MeanDiff	SEM	q Value	Prob	Alpha	Sig	LCL	UCL
Female Male	0.19114	1.14028	0.23705	0.86719	0.05	0	-2.06864	2.45091

Genotype

	MeanDiff	SEM	q Value	Prob	Alpha	Sig	LCL	UCL
WT PostnHet	-1.89569	1.59704	-1.67867	0.63633	0.05	0	-6.06196	2.27058
WT PostnKO	-1.45242	1.56648	-1.31124	0.79035	0.05	0	-5.53897	2.63413
WT Spp1KO	1.83358	1.59148	1.62934	0.65814	0.05	0	-2.31819	5.98535
PostnHet PostnKO	0.44326	1.63345	0.38377	0.99297	0.05	0	-3.81799	4.70452
PostnHet Spp1KO	3.72926	1.65744	3.182	0.11642	0.05	0	-0.59457	8.0531
PostnKO Spp1KO	3.286	1.62802	2.85446	0.18751	0.05	0	-0.96107	7.53307

Days Post-Fracture 's

	Mean	Groups		
10 dpf	30.55722	A		
14 dpf	20.04093		B	
7 dpf	18.87724		B	
21 dpf	2.50324			C

Means that do not share a letter are significantly different.

Gender 's Grouping Le

	Mean	Groups
Male	18.8697	A
Female	17.73467	A

Means that do not share a letter are significantly different.

Sig equals 1 indicates that the difference of the means is significant at the 0.05 level.
Sig equals 0 indicates that the difference of the means is not significant at the 0.05 level.

ANOVAThreeWay (7/24/2023 14:41:56)

ANOVA

Means Comparisons

Tukey Test

Genotype 's Grouping

	Mean	Groups
PostnHet	20.575	A
PostnKO	18.35257	A
WT	17.8045	A
Spp1KO	16.37273	A

Means that do not share a letter are significantly different.

Interactions 's Groupin

Days Post-Fracture	Gender	Genotype	Mean	Groups								
10 dpf	Female	PostnKO	44.59333	A								
10 dpf	Female	PostnHet	35.948	A	B							
14 dpf	Male	PostnKO	35.2275	A	B							
10 dpf	Male	PostnHet	32.8775	A	B	C						
10 dpf	Male	WT	31.815	A	B	C						
10 dpf	Female	WT	30.715	A	B	C	D					
14 dpf	Male	PostnHet	26.86	A	B	C	D	E				
7 dpf	Female	WT	26.41	A	B	C	D	E				
7 dpf	Female	Spp1KO	26.17	A	B	C	D	E				
10 dpf	Female	Spp1KO	25.71		B	C	D	E				
10 dpf	Male	Spp1KO	24.58		B	C	D	E				
10 dpf	Male	PostnKO	22.882		B	C	D	E				
14 dpf	Female	PostnHet	22.55429		B	C	D	E				
7 dpf	Male	WT	21.525		B	C	D	E	F			
14 dpf	Female	PostnKO	21.30167		B	C	D	E				
14 dpf	Male	Spp1KO	20.065		B	C	D	E	F	G		
7 dpf	Male	PostnHet	18.64		B	C	D	E	F	G	H	I
7 dpf	Female	PostnHet	17.50333		B	C	D	E	F	G	H	I
14 dpf	Male	WT	16.39429				D	E	F	G	H	
7 dpf	Male	Spp1KO	15.13667			C	D	E	F	G	H	I
14 dpf	Female	Spp1KO	12.816					E	F	G	H	I
7 dpf	Male	PostnKO	12.6325					E	F	G	H	I
7 dpf	Female	PostnKO	11.6625					E	F	G	H	I
14 dpf	Female	WT	11.43667					E	F	G	H	I
21 dpf	Male	PostnKO	4.1875						F	G	H	I
21 dpf	Male	Spp1KO	3.468							G	H	I
21 dpf	Female	WT	2.89833								H	I
21 dpf	Male	PostnHet	2.6						F	G	H	I
21 dpf	Female	PostnKO	2.3								H	I
21 dpf	Male	WT	1.97333							G	H	I
21 dpf	Female	PostnHet	1.35									I
21 dpf	Female	Spp1KO	0.55333							G	H	I

Means that do not share a letter are significantly different.

Sig equals 1 indicates that the difference of the means is significant at the 0.05 level.
Sig equals 0 indicates that the difference of the means is not significant at the 0.05 level.

ANOVAThreeWay (7/13/2023 12:04:50)

Descriptive Statistics

Table S3-D. Histomorphometry: TRAP+ Cells per mm^2 of Callus Area

unit of measure = cells/mm^2

Days Post-Fracture

	N	Mean	SD	SEM	Variance	Missing	NonMissing
10 dpf	35	14.07646	7.17996	1.24987	51.55185	2	33
14 dpf	42	45.24808	10.17283	1.60846	103.48638	2	40
21 dpf	38	51.46	9.80705	1.61227	96.17819	1	37

Gender

	N	Mean	SD	SEM	Variance	Missing	NonMissing
Female	63	40.15351	17.3742	2.26193	301.86267	4	59
Male	52	35.47862	19.35664	2.71047	374.6796	1	51

Genotype

	N	Mean	SD	SEM	Variance	Missing	NonMissing
WT	35	47.8463	18.35612	3.14805	336.94721	1	34
PostnHet	28	34.33865	15.98804	3.13551	255.61756	2	26
PostnKO	27	31.87593	17.05916	3.34558	291.01503	1	26
Spp1KO	25	34.58804	17.63821	3.60038	311.10651	1	24

*Days Post-Fracture*Gender*

		N	Mean	SD	SEM	Variance	Missing	NonMissing
10 dpf	Female	17	14.55366	6.83957	1.76597	46.77977	2	15
	Male	18	13.67879	7.62535	1.79731	58.1459	0	18
14 dpf	Female	24	47.45955	8.52196	1.81689	72.62373	2	22
	Male	18	42.54517	11.56001	2.72472	133.63381	0	18
21 dpf	Female	22	50.3019	9.89862	2.11039	97.98267	0	22
	Male	16	53.15854	9.75411	2.5185	95.14266	1	15

*Days Post-Fracture*Genotype*

		N	Mean	SD	SEM	Variance	Missing	NonMissing
10 dpf	WT	10	21.57464	6.36762	2.01362	40.54658	0	10
	PostnHet	9	12.62193	4.21571	1.49048	17.7722	1	8
	PostnKO	8	8.88441	6.05659	2.14133	36.68233	0	8
	Spp1KO	8	10.96087	2.60764	0.9856	6.7998	1	7
14 dpf	WT	13	55.34101	6.47298	1.79528	41.89949	0	13
	PostnHet	11	43.66005	7.90031	2.4983	62.41495	1	10
	PostnKO	9	39.98841	7.82704	2.76728	61.26262	1	8
	Spp1KO	9	37.10914	6.85216	2.28405	46.95214	0	9
21 dpf	WT	12	62.87225	3.68446	1.11091	13.57527	1	11
	PostnHet	8	44.40361	6.00068	2.12156	36.00815	0	8
	PostnKO	10	43.77917	6.81104	2.15384	46.3902	0	10
	Spp1KO	8	52.42558	5.71598	2.02091	32.67247	0	8

*Gender*Genotype*

		N	Mean	SD	SEM	Variance	Missing	NonMissing
Female	WT	17	50.7335	16.78166	4.07015	281.62412	0	17
	PostnHet	17	37.59095	15.30403	3.95148	234.21324	2	15
	PostnKO	16	36.53543	15.93945	4.11555	254.06601	1	15
	Spp1KO	13	32.89098	17.41325	5.02677	303.22116	1	12
Male	WT	18	44.95911	19.89009	4.82406	395.61569	1	17
	PostnHet	11	29.90368	16.54217	4.98765	273.6433	0	11
	PostnKO	11	25.52208	17.17179	5.17749	294.87027	0	11
	Spp1KO	12	36.28511	18.46593	5.33065	340.99057	0	12

ANOVAThreeWay (7/13/2023 12:04:50)

Descriptive Statistics

*Days Post-Fracture*Gender*Genotype*

			N	Mean	SD	SEM	Variance	Missing	NonMissing
10 dpf	Female	WT	4	22.3336	8.2791	4.13955	68.54351	0	4
		PostnHet	5	14.12585	2.42839	1.2142	5.89709	1	4
		PostnKO	3	7.62853	1.77677	1.02582	3.15692	0	3
		Spp1KO	5	12.39538	2.57774	1.28887	6.64474	1	4
	Male	WT	6	21.06867	5.57582	2.27632	31.08972	0	6
		PostnHet	4	11.118	5.43507	2.71753	29.53993	0	4
		PostnKO	5	9.63794	7.79249	3.48491	60.72295	0	5
		Spp1KO	3	9.0482	0.91062	0.52575	0.82923	0	3
14 dpf	Female	WT	6	57.62917	1.2035	0.49133	1.4484	0	6
		PostnHet	7	47.07155	3.35204	1.36847	11.23619	1	6
		PostnKO	6	45.2762	3.23733	1.44778	10.48033	1	5
		Spp1KO	5	37.90496	8.91681	3.98772	79.50951	0	5
	Male	WT	7	53.37973	8.53631	3.22642	72.86859	0	7
		PostnHet	4	38.5428	10.50363	5.25181	110.32621	0	4
		PostnKO	3	31.17543	2.65591	1.53339	7.05388	0	3
		Spp1KO	4	36.11438	4.10098	2.05049	16.81806	0	4
21 dpf	Female	WT	7	61.05144	2.46222	0.93063	6.06253	0	7
		PostnHet	5	44.98632	6.8906	3.08157	47.48034	0	5
		PostnKO	7	42.68069	7.69749	2.90938	59.25142	0	7
		Spp1KO	3	51.8618	7.4768	4.31673	55.90259	0	3
	Male	WT	5	66.05868	3.44272	1.72136	11.85233	1	4
		PostnHet	3	43.43243	5.36695	3.09861	28.80419	0	3
		PostnKO	3	46.3423	4.11386	2.37514	16.92387	0	3
		Spp1KO	5	52.76384	5.37067	2.40184	28.84412	0	5

ANOVAThreeWay (7/13/2023 12:04:50)

ANOVA

Overall ANOVA

	DF	Sum of Squares	Mean Square	F Value	P Value
Days Post-Fracture	2	25633.67054	12816.83527	376.15238	3.0002E-43
Gender	1	121.46541	121.46541	3.5648	0.06239
Genotype	3	4826.12973	1608.70991	47.21291	3.93307E-18
Days Post-Fracture*Gender	2	380.8735	190.43675	5.589	0.00522
Days Post-Fracture*Genotype	6	558.74763	93.12461	2.73305	0.0177
Gender*Genotype	3	67.38298	22.46099	0.65919	0.57939
Days Post-Fracture*Gender*Genotype	6	200.28214	33.38036	0.97966	0.44408
Model	23	33909.51435	1474.32671	43.26899	9.91321E-38
Error	86	2930.32267	34.07352		
Corrected Total	109	36839.83702			

At the 0.05 level, the population means of **Days Post-Fracture** are **significantly** different.
At the 0.05 level, the population means of **Gender** are **not significantly** different.
At the 0.05 level, the population means of **Genotype** are **significantly** different.
At the 0.05 level, the population means of **Days Post-Fracture*Gender** are **significantly** different.
At the 0.05 level, the population means of **Days Post-Fracture*Genotype** are **significantly** different.
At the 0.05 level, the population means of **Gender*Genotype** are **not significantly** different.
At the 0.05 level, the population means of **Days Post-Fracture*Gender*Genotype** are **not significantly** different.

ANOVAThreeWay (7/13/2023 12:04:50)

ANOVA

Means Comparisons

Tukey Test

Days Post-Fracture

	MeanDiff	SEM	q Value	Prob	Alpha	Sig	LCL	UCL
10 dpf 14 dpf	-29.96726	1.24817	-33.95384	0	0.05	1	-32.94409	-26.99042
10 dpf 21 dpf	-37.72767	1.28533	-41.51073	0	0.05	1	-40.79313	-34.66221
14 dpf 21 dpf	-7.76041	1.23178	-8.90975	6.73231E-9	0.05	1	-10.69817	-4.82266

Gender

	MeanDiff	SEM	q Value	Prob	Alpha	Sig	LCL	UCL
Female Male	2.18859	1.02494	3.01981	0.03558	0.05	1	0.15107	4.22611

Genotype

	MeanDiff	SEM	q Value	Prob	Alpha	Sig	LCL	UCL
WT PostnHet	13.70739	1.37878	14.05962	0	0.05	1	10.095	17.31978
WT PostnKO	16.46336	1.40348	16.58927	1.18606E-7	0.05	1	12.78627	20.14046
WT Spp1KO	13.57212	1.40974	13.61516	0	0.05	1	9.87862	17.26563
PostnHet PostnKO	2.75598	1.48817	2.61901	0.25659	0.05	0	-1.14301	6.65496
PostnHet Spp1KO	-0.13527	1.49408	-0.12804	0.99973	0.05	0	-4.04973	3.7792
PostnKO Spp1KO	-2.89124	1.5169	-2.69552	0.23318	0.05	0	-6.8655	1.08301

Days Post-Fracture 's

	Mean	Groups		
21 dpf	51.46	A		
14 dpf	45.24808		B	
10 dpf	14.07646			C

Means that do not share a letter are significantly different.

Gender 's Grouping Le

	Mean	Groups	
Female	40.15351	A	
Male	35.47862		B

Means that do not share a letter are significantly different.

Genotype 's Grouping

	Mean	Groups	
WT	47.8463	A	
Spp1KO	34.58804		B
PostnHet	34.33865		B
PostnKO	31.87593		B

Means that do not share a letter are significantly different.

Sig equals 1 indicates that the difference of the means is significant at the 0.05 level.
Sig equals 0 indicates that the difference of the means is not significant at the 0.05 level.

ANOVAThreeWay (7/13/2023 12:04:50)

ANOVA

Means Comparisons

Tukey Test

Interactions 's Groupin

Days Post-Fracture	Gender	Genotype	Mean	Groups									
21 dpf	Male	WT	66.05868	A									
21 dpf	Female	WT	61.05144	A	B								
14 dpf	Female	WT	57.62917	A	B	C							
14 dpf	Male	WT	53.37973		B	C	D						
21 dpf	Male	Spp1KO	52.76384		B	C	D	E					
21 dpf	Female	Spp1KO	51.8618	A	B	C	D	E	F				
14 dpf	Female	PostnHet	47.07155			C	D	E	F	G			
21 dpf	Male	PostnKO	46.3423			C	D	E	F	G	H		
14 dpf	Female	PostnKO	45.2762				D	E	F	G	H		
21 dpf	Female	PostnHet	44.98632				D	E	F	G	H		
21 dpf	Male	PostnHet	43.43243				D	E	F	G	H		
21 dpf	Female	PostnKO	42.68069					E	F	G	H		
14 dpf	Male	PostnHet	38.5428						F	G	H		
14 dpf	Female	Spp1KO	37.90496						F	G	H		
14 dpf	Male	Spp1KO	36.11438							G	H		
14 dpf	Male	PostnKO	31.17543								H	I	
10 dpf	Female	WT	22.3336									I	J
10 dpf	Male	WT	21.06867									I	J
10 dpf	Female	PostnHet	14.12585										J
10 dpf	Female	Spp1KO	12.39538										J
10 dpf	Male	PostnHet	11.118										J
10 dpf	Male	PostnKO	9.63794										J
10 dpf	Male	Spp1KO	9.0482										J
10 dpf	Female	PostnKO	7.62853										J

Means that do not share a letter are significantly different.

Sig equals 1 indicates that the difference of the means is significant at the 0.05 level.
Sig equals 0 indicates that the difference of the means is not significant at the 0.05 level.

ANOVAThreeWay (7/26/2023 13:51:39)

Descriptive Statistics

Table S4-A. Cartilage IHC Callus Percent COL10A1 Area unit of measure = percent (COL10A1 area/Callus Area)

Days Post-Fracture

	N	Mean	SD	SEM	Variance	Missing	NonMissing
7 dpf	29	16.72808	9.84403	1.93057	96.90494	3	26
10 dpf	38	29.395	9.36074	1.91075	87.62341	14	24
14 dpf	66	26.72667	9.17076	1.76491	84.10285	39	27

Gender

	N	Mean	SD	SEM	Variance	Missing	NonMissing
Female	69	24.28925	10.24674	1.62015	104.99573	29	40
Male	64	24.06649	11.54758	1.89841	133.34658	27	37

Genotype

	N	Mean	SD	SEM	Variance	Missing	NonMissing
WT	33	28.94632	9.2243	2.1162	85.08767	14	19
PostnHet	34	25.96789	10.3854	2.38257	107.85655	15	19
PostnKO	33	21.12421	11.37843	2.61039	129.46876	14	19
Spp1KO	33	20.865	10.76851	2.40791	115.96078	13	20

*Days Post-Fracture*Gender*

		N	Mean	SD	SEM	Variance	Missing	NonMissing
7 dpf	Female	15	18.88231	11.15328	3.09336	124.39559	2	13
	Male	14	14.57385	8.21187	2.27756	67.43484	1	13
10 dpf	Female	18	30.105	9.77763	2.82256	95.60199	6	12
	Male	20	28.685	9.30111	2.685	86.51074	8	12
14 dpf	Female	36	24.32267	7.38975	1.90802	54.60836	21	15
	Male	30	29.73167	10.56198	3.04898	111.55532	18	12

*Days Post-Fracture*Genotype*

		N	Mean	SD	SEM	Variance	Missing	NonMissing
7 dpf	WT	8	25.55429	10.57247	3.99602	111.77716	1	7
	PostnHet	6	16.11167	7.93055	3.23764	62.8937	0	6
	PostnKO	8	14.05429	9.01395	3.40695	81.2513	1	7
	Spp1KO	7	10.16667	4.33668	1.77044	18.80683	1	6
10 dpf	WT	10	30.17333	8.85852	3.61648	78.47343	4	6
	PostnHet	9	32.41167	8.79111	3.58896	77.2837	3	6
	PostnKO	8	29.60333	7.66313	3.12846	58.72359	2	6
	Spp1KO	11	25.39167	12.5561	5.12601	157.65558	5	6
14 dpf	WT	15	31.67667	8.17612	3.33789	66.84891	9	6
	PostnHet	19	28.89286	7.62276	2.88113	58.10642	12	7
	PostnKO	17	20.89333	12.39139	5.05876	153.54643	11	6
	Spp1KO	15	25.49375	6.96799	2.46356	48.55286	7	8

ANOVAThreeWay (7/26/2023 13:51:39)

Descriptive Statistics

*Gender*Genotype*

		N	Mean	SD	SEM	Variance	Missing	NonMissing
Female	WT	17	32.19778	8.65331	2.88444	74.87974	8	9
	PostnHet	19	28.686	8.04444	2.54388	64.713	9	10
	PostnKO	16	17.619	10.53712	3.33213	111.03092	6	10
	Spp1KO	17	19.88545	7.06847	2.13122	49.96329	6	11
Male	WT	16	26.02	9.13941	2.89013	83.52876	6	10
	PostnHet	15	22.94778	12.2629	4.08763	150.37874	6	9
	PostnKO	17	25.01889	11.57464	3.85821	133.97221	8	9
	Spp1KO	16	22.06222	14.4921	4.8307	210.02092	7	9

*Days Post-Fracture*Gender*Genotype*

			N	Mean	SD	SEM	Variance	Missing	NonMissing
7 dpf	Female	WT	4	34.05333	10.93522	6.31345	119.57903	1	3
		PostnHet	3	21.58333	6.20378	3.58176	38.48693	0	3
		PostnKO	4	9.6	1.30448	0.65224	1.70167	0	4
		Spp1KO	4	13.38667	3.45632	1.99551	11.94613	1	3
	Male	WT	4	19.18	4.17442	2.08721	17.4258	0	4
		PostnHet	3	10.64	5.37865	3.10537	28.9299	0	3
		PostnKO	4	19.99333	12.19064	7.03827	148.61163	1	3
		Spp1KO	3	6.94667	1.99141	1.14974	3.96573	0	3
10 dpf	Female	WT	4	33.56333	6.59267	3.80628	43.46323	1	3
		PostnHet	5	36.47333	8.28629	4.78409	68.66253	2	3
		PostnKO	3	29.77667	10.16347	5.86788	103.29613	0	3
		Spp1KO	6	20.60667	9.8044	5.66057	96.12623	3	3
	Male	WT	6	26.78333	10.87401	6.27811	118.24403	3	3
		PostnHet	4	28.35	8.66345	5.00184	75.0553	1	3
		PostnKO	5	29.43	6.58959	3.8045	43.4227	2	3
		Spp1KO	5	30.17667	15.14345	8.74307	229.32403	2	3
14 dpf	Female	WT	9	28.97667	10.62787	6.136	112.95163	6	3
		PostnHet	11	28.1725	3.35619	1.6781	11.26402	7	4
		PostnKO	9	16.15333	6.34399	3.66271	40.24623	6	3
		Spp1KO	7	23.352	4.87699	2.18106	23.78507	2	5
	Male	WT	6	34.37667	5.68336	3.28129	32.30063	3	3
		PostnHet	8	29.85333	12.44997	7.18799	155.00163	5	3
		PostnKO	8	25.63333	16.61978	9.59543	276.21703	5	3
		Spp1KO	8	29.06333	9.58041	5.53125	91.78423	5	3

ANOVAThreeWay (7/26/2023 13:51:39)

Descriptive Statistics

ANOVA

Overall ANOVA

	DF	Sum of Squares	Mean Square	F Value	P Value
Days Post-Fracture	2	2199.23392	1099.61696	15.09233	6.48881E-6
Gender	1	3.64795	3.64795	0.05007	0.8238
Genotype	3	936.26566	312.08855	4.28344	0.00884
Days Post-Fracture*Gender	2	403.07776	201.53888	2.76614	0.072
Days Post-Fracture*Genotype	6	546.96851	91.16142	1.2512	0.29575
Gender*Genotype	3	533.05969	177.68656	2.43876	0.07459
Days Post-Fracture*Gender*Genotype	6	410.16485	68.36081	0.93826	0.47568
Model	23	5034.71944	218.90085	3.00443	4.84751E-4
Error	53	3861.54469	72.85933		
Corrected Total	76	8896.26412			

At the 0.05 level, the population means of **Days Post-Fracture** are **significantly** different.
At the 0.05 level, the population means of **Gender** are **not significantly** different.
At the 0.05 level, the population means of **Genotype** are **significantly** different.
At the 0.05 level, the population means of **Days Post-Fracture*Gender** are **not significantly** different.
At the 0.05 level, the population means of **Days Post-Fracture*Genotype** are **not significantly** different.
At the 0.05 level, the population means of **Gender*Genotype** are **not significantly** different.
At the 0.05 level, the population means of **Days Post-Fracture*Gender*Genotype** are **not significantly** different.

ANOVAThreeWay (7/26/2023 13:51:39)

Descriptive Statistics

ANOVA

Means Comparisons

Tukey Test

Days Post-Fracture

	MeanDiff	SEM	q Value	Prob	Alpha	Sig	LCL	UCL
7 dpf 10 dpf	-12.47208	2.0121	-8.76604	2.32303E-7	0.05	1	-17.32372	-7.62045
7 dpf 14 dpf	-10.02473	1.96946	-7.19846	1.42501E-5	0.05	1	-14.77355	-5.27591
10 dpf 14 dpf	2.44735	2.00234	1.72851	0.44549	0.05	0	-2.38075	7.27546

Gender

	MeanDiff	SEM	q Value	Prob	Alpha	Sig	LCL	UCL
Female Male	0.43926	1.62868	0.38142	0.78843	0.05	0	-2.82746	3.70599

Genotype

	MeanDiff	SEM	q Value	Prob	Alpha	Sig	LCL	UCL
WT PostnHet	3.64347	2.31085	2.22976	0.40046	0.05	0	-2.48596	9.77291
WT PostnKO	7.72444	2.31085	4.72727	0.00808	0.05	1	1.59501	13.85388
WT Spp1KO	8.90022	2.29573	5.4827	0.00163	0.05	1	2.81089	14.98955
PostnHet PostnKO	4.08097	2.31085	2.49751	0.30094	0.05	0	-2.04846	10.21041
PostnHet Spp1KO	5.25675	2.29573	3.23826	0.11337	0.05	0	-0.83258	11.34608
PostnKO Spp1KO	1.17578	2.29573	0.7243	0.95584	0.05	0	-4.91355	7.26511

Days Post-Fracture 's

	Mean	Groups	
10 dpf	29.395	A	
14 dpf	26.72667	A	
7 dpf	16.72808		B

Means that do not share a letter are significantly different.

Gender 's Grouping Le

	Mean	Groups
Female	24.28925	A
Male	24.06649	A

Means that do not share a letter are significantly different.

Genotype 's Grouping

	Mean	Groups	
WT	28.94632	A	
PostnHet	25.96789	A	B
PostnKO	21.12421		B
Spp1KO	20.865		B

Means that do not share a letter are significantly different.

Sig equals 1 indicates that the difference of the means is significant at the 0.05 level.
Sig equals 0 indicates that the difference of the means is not significant at the 0.05 level.

ANOVAThreeWay (7/26/2023 13:51:39)

Descriptive Statistics

ANOVA

Means Comparisons

Tukey Test

Interactions 's Groupin

Days Post-Fracture	Gender	Genotype	Mean	Groups			
10 dpf	Female	PostnHet	36.47333	A			
14 dpf	Male	WT	34.37667	A	B		
7 dpf	Female	WT	34.05333	A	B		
10 dpf	Female	WT	33.56333	A	B		
10 dpf	Male	Spp1KO	30.17667	A	B	C	
14 dpf	Male	PostnHet	29.85333	A	B	C	
10 dpf	Female	PostnKO	29.77667	A	B	C	
10 dpf	Male	PostnKO	29.43	A	B	C	
14 dpf	Male	Spp1KO	29.06333	A	B	C	D
14 dpf	Female	WT	28.97667	A	B	C	D
10 dpf	Male	PostnHet	28.35	A	B	C	D
14 dpf	Female	PostnHet	28.1725	A	B	C	
10 dpf	Male	WT	26.78333	A	B	C	D
14 dpf	Male	PostnKO	25.63333	A	B	C	D
14 dpf	Female	Spp1KO	23.352	A	B	C	D
7 dpf	Female	PostnHet	21.58333	A	B	C	D
10 dpf	Female	Spp1KO	20.60667	A	B	C	D
7 dpf	Male	PostnKO	19.99333	A	B	C	D
7 dpf	Male	WT	19.18	A	B	C	D
14 dpf	Female	PostnKO	16.15333	A	B	C	D
7 dpf	Female	Spp1KO	13.38667		B	C	D
7 dpf	Male	PostnHet	10.64			C	D
7 dpf	Female	PostnKO	9.6			C	D
7 dpf	Male	Spp1KO	6.94667				D

Means that do not share a letter are significantly different.

Sig equals 1 indicates that the difference of the means is significant at the 0.05 level.
Sig equals 0 indicates that the difference of the means is not significant at the 0.05 level.

ANOVAThreeWay (7/26/2023 15:13:10)

Descriptive Statistics

Table S4-B. Callus Chondrocytes

unit of measure = cell counts

Days Post-Fracture

	N	Mean	SD	SEM	Variance	Missing	NonMissing
10 dpf	32	3469.39286	1242.35056	234.78219	1543434.91402	4	28
14 dpf	36	2389.53125	1005.71672	177.78728	1011466.12802	4	32

Gender

	N	Mean	SD	SEM	Variance	Missing	NonMissing
Female	40	2520.78788	1219.23072	212.24083	1486523.54735	7	33
Male	28	3348.96296	1121.2628	215.78713	1257230.26781	1	27

Genotype

	N	Mean	SD	SEM	Variance	Missing	NonMissing
WT	18	3554.27778	1223.73891	288.43803	1497536.9183	0	18
PostnHet	19	2819.30769	926.48866	256.96172	858381.23077	6	13
PostnKO	16	2671.6	963.54248	248.7856	928414.11429	1	15
Spp1KO	15	2350.42857	1490.66463	398.39688	2222081.03297	1	14

*Days Post-Fracture*Gender*

		N	Mean	SD	SEM	Variance	Missing	NonMissing
10 dpf	Female	16	3188.69231	1124.27268	311.81714	1263989.0641	3	13
	Male	16	3712.66667	1325.3796	342.21154	1756631.09524	1	15
14 dpf	Female	24	2086.65	1096.21642	245.12144	1201690.45	4	20
	Male	12	2894.33333	574.51075	165.84697	330062.60606	0	12

*Days Post-Fracture*Genotype*

		N	Mean	SD	SEM	Variance	Missing	NonMissing
10 dpf	WT	9	4251.55556	1293.56949	431.18983	1673322.02778	0	9
	PostnHet	9	3448.16667	654.12825	267.04674	427883.76667	3	6
	PostnKO	7	3041	651.96881	246.42105	425063.33333	0	7
	Spp1KO	7	2817.16667	1688.23108	689.21745	2850124.16667	1	6
14 dpf	WT	9	2857	644.20668	214.73556	415002.25	0	9
	PostnHet	10	2280.28571	791.05009	298.98883	625760.2381	3	7
	PostnKO	9	2348.375	1112.03982	393.16545	1236632.55357	1	8
	Spp1KO	8	2000.375	1328.21394	469.59454	1764152.26786	0	8

ANOVAThreeWay (7/26/2023 15:13:10)

Descriptive Statistics

*Gender*Genotype*

		N	Mean	SD	SEM	Variance	Missing	NonMissing
Female	WT	10	3231.7	962.30996	304.30913	926040.45556	0	10
	PostnHet	12	2486.85714	1078.27106	407.54815	1162668.47619	5	7
	PostnKO	9	2363.125	1141.29368	403.50825	1302551.26786	1	8
	Spp1KO	9	1819.5	1411.44313	499.0205	1992171.71429	1	8
Male	WT	8	3957.5	1453.19815	513.78313	2111784.85714	0	8
	PostnHet	7	3207.16667	574.15901	234.39943	329658.56667	1	6
	PostnKO	7	3024.14286	612.19262	231.38706	374779.80952	0	7
	Spp1KO	6	3058.33333	1391.40184	568.03742	1935999.06667	0	6

*Days Post-Fracture*Gender*Genotype*

			N	Mean	SD	SEM	Variance	Missing	NonMissing
10 dpf	Female	WT	4	3985.5	1038.00755	519.00377	1077459.66667	0	4
		PostnHet	5	3574	581.29081	335.6084	337899	2	3
		PostnKO	3	2585	470.94267	271.89888	221787	0	3
		Spp1KO	4	2344.66667	1516.68234	875.65696	2300325.33333	1	3
	Male	WT	5	4464.4	1552.78308	694.42571	2411135.3	0	5
		PostnHet	4	3322.33333	827.22931	477.60107	684308.33333	1	3
		PostnKO	4	3383	581.70497	290.85248	338380.66667	0	4
		Spp1KO	3	3289.66667	2038.43478	1176.89087	4155216.33333	0	3
14 dpf	Female	WT	6	2729.16667	512.61854	209.27564	262777.76667	0	6
		PostnHet	7	1671.5	178.27413	89.13707	31781.66667	3	4
		PostnKO	6	2230	1452.41075	649.53784	2109497	1	5
		Spp1KO	5	1504.4	1416.04301	633.27368	2005177.8	0	5
	Male	WT	3	3112.66667	925.19854	534.16362	855992.33333	0	3
		PostnHet	3	3092	316.30365	182.618	100048	0	3
		PostnKO	3	2545.66667	125.70733	72.57716	15802.33333	0	3
		Spp1KO	3	2827	724.04144	418.02552	524236	0	3

ANOVAThreeWay (7/26/2023 15:13:10)

Descriptive Statistics

ANOVA

Overall ANOVA

	DF	Sum of Squares	Mean Square	F Value	P Value
Days Post-Fracture	1	1.15931E7	1.15931E7	10.17115	0.00263
Gender	1	6486012.45387	6486012.45387	5.69048	0.02143
Genotype	3	1.03539E7	3451299.97716	3.02799	0.03931
Days Post-Fracture*Gender	1	479752.50209	479752.50209	0.42091	0.51986
Days Post-Fracture*Genotype	3	1305525.55913	435175.18638	0.3818	0.7666
Gender*Genotype	3	1025111.94703	341703.98234	0.29979	0.82535
Days Post-Fracture*Gender*Genotype	3	2223380.86874	741126.95625	0.65023	0.58704
Model	15	4.02907E7	2686049.62444	2.3566	0.01393
Error	44	5.01512E7	1139800.55833		
Corrected Total	59	9.0442E7			

At the 0.05 level, the population means of **Days Post-Fracture** are **significantly** different.
At the 0.05 level, the population means of **Gender** are **significantly** different.
At the 0.05 level, the population means of **Genotype** are **significantly** different.
At the 0.05 level, the population means of **Days Post-Fracture*Gender** are **not significantly** different.
At the 0.05 level, the population means of **Days Post-Fracture*Genotype** are **not significantly** different.
At the 0.05 level, the population means of **Gender*Genotype** are **not significantly** different.
At the 0.05 level, the population means of **Days Post-Fracture*Gender*Genotype** are **not significantly** different.

ANOVA
Means Comparisons
Tukey Test
Days Post-Fracture

	MeanDiff	SEM	q Value	Prob	Alpha	Sig	LCL	UCL
10 dpf 14 dpf	904.52083	242.87576	5.26683	5.54525E-4	0.05	1	415.03606	1394.0056

Gender

	MeanDiff	SEM	q Value	Prob	Alpha	Sig	LCL	UCL
Female Male	-676.5625	242.87576	-3.93948	0.00785	0.05	1	-1166.04727	-187.07773

Genotype

	MeanDiff	SEM	q Value	Prob	Alpha	Sig	LCL	UCL
WT PostnHet	657.975	339.01311	2.74478	0.22622	0.05	0	-247.192	1563.142
WT PostnKO	887.01667	328.57943	3.81774	0.04663	0.05	1	9.70766	1764.32567
WT Spp1KO	1081.5	335.13855	4.5637	0.0122	0.05	1	186.67811	1976.32189
PostnHet PostnKO	229.04167	351.62011	0.9212	0.9145	0.05	0	-709.78608	1167.86941
PostnHet Spp1KO	423.525	357.75705	1.67419	0.64012	0.05	0	-531.68841	1378.73841
PostnKO Spp1KO	194.48333	347.88598	0.79061	0.94358	0.05	0	-734.37428	1123.34095

Days Post-Fracture 's

	Mean	Groups	
10 dpf	3469.39286	A	
14 dpf	2389.53125		B

Means that do not share a letter are significantly different.

Gender 's Grouping L

	Mean	Groups	
Male	3348.96296	A	
Female	2520.78788		B

Means that do not share a letter are significantly different.

Genotype 's Grouping

	Mean	Groups	
WT	3554.27778	A	
PostnHet	2819.30769	A	B
PostnKO	2671.6		B
Spp1KO	2350.42857		B

Means that do not share a letter are significantly different.

Sig equals 1 indicates that the difference of the means is significant at the 0.05 level.
Sig equals 0 indicates that the difference of the means is not significant at the 0.05 level.

ANOVAThreeWay (7/26/2023 15:13:10)

Descriptive Statistics

ANOVA

Means Comparisons

Tukey Test

Interactions 's Groupi

Days Post-Fracture	Gender	Genotype	Mean	Groups		
10 dpf	Male	WT	4464.4	A		
10 dpf	Female	WT	3985.5	A	B	
10 dpf	Female	PostnHet	3574	A	B	C
10 dpf	Male	PostnKO	3383	A	B	C
10 dpf	Male	PostnHet	3322.33333	A	B	C
10 dpf	Male	Spp1KO	3289.66667	A	B	C
14 dpf	Male	WT	3112.66667	A	B	C
14 dpf	Male	PostnHet	3092	A	B	C
14 dpf	Male	Spp1KO	2827	A	B	C
14 dpf	Female	WT	2729.16667	A	B	C
10 dpf	Female	PostnKO	2585	A	B	C
14 dpf	Male	PostnKO	2545.66667	A	B	C
10 dpf	Female	Spp1KO	2344.66667	A	B	C
14 dpf	Female	PostnKO	2230		B	C
14 dpf	Female	PostnHet	1671.5		B	C
14 dpf	Female	Spp1KO	1504.4			C

Means that do not share a letter are significantly different.

Sig equals 1 indicates that the difference of the means is significant at the 0.05 level.
Sig equals 0 indicates that the difference of the means is not significant at the 0.05 level.

ANOVAThreeWay (7/13/2023 15:09:07)
Descriptive Statistics

Table S4-C. Cartilage IHC Percent MMP13+ Chondrocytes
unit of measure = percent (MMP13+ Cells/No. of Chondrocytes)

Days Post-Fracture

	N	Mean	SD	SEM	Variance	Missing	NonMissing
10 dpf	32	24.69929	7.89707	1.49241	62.36365	4	28
14 dpf	36	42.54875	9.71215	1.71688	94.32588	4	32

Gender

	N	Mean	SD	SEM	Variance	Missing	NonMissing
Female	40	36.05727	11.89122	2.07	141.40116	7	33
Male	28	31.97222	13.29124	2.5579	176.65702	1	27

Genotype

	N	Mean	SD	SEM	Variance	Missing	NonMissing
WT	18	43.69333	12.09664	2.85121	146.32879	0	18
PostnHet	19	29.38231	10.31481	2.86081	106.39522	6	13
PostnKO	16	29.88867	11.25448	2.90589	126.66323	1	15
Spp1KO	15	31.16857	10.72995	2.8677	115.13192	1	14

*Days Post-Fracture*Gender*

		N	Mean	SD	SEM	Variance	Missing	NonMissing
10 dpf	Female	16	25.46231	8.03648	2.22892	64.58502	3	13
	Male	16	24.038	7.99406	2.06406	63.90502	1	15
14 dpf	Female	24	42.944	8.40421	1.87924	70.63074	4	20
	Male	12	41.89	11.96125	3.45292	143.07147	0	12

*Days Post-Fracture*Genotype*

		N	Mean	SD	SEM	Variance	Missing	NonMissing
10 dpf	WT	9	32.83889	6.08347	2.02782	37.00859	0	9
	PostnHet	9	20.28333	6.67383	2.72458	44.53995	3	6
	PostnKO	7	22.08714	5.59665	2.11533	31.32246	0	7
	Spp1KO	7	19.95333	4.02308	1.64242	16.18519	1	6
14 dpf	WT	9	54.54778	2.97446	0.99149	8.84742	0	9
	PostnHet	10	37.18143	4.68172	1.76952	21.91848	3	7
	PostnKO	9	36.715	10.59996	3.74765	112.35906	1	8
	Spp1KO	8	39.58	3.68554	1.30304	13.58323	0	8

ANOVAThreeWay (7/13/2023 15:09:07)

Descriptive Statistics

*Gender*Genotype*

		N	Mean	SD	SEM	Variance	Missing	NonMissing
Female	WT	10	45.453	12.3584	3.90807	152.73007	0	10
	PostnHet	12	30.33571	7.38475	2.79117	54.53446	5	7
	PostnKO	9	32.245	10.00847	3.53853	100.16946	1	8
	Spp1KO	9	33.13125	10.85727	3.83863	117.88041	1	8
Male	WT	8	41.49375	12.2086	4.31639	149.04988	0	8
	PostnHet	7	28.27	13.68027	5.58495	187.14992	1	6
	PostnKO	7	27.19571	12.76007	4.82285	162.81943	0	7
	Spp1KO	6	28.55167	10.95122	4.47082	119.92922	0	6

*Days Post-Fracture*Gender*Genotype*

			N	Mean	SD	SEM	Variance	Missing	NonMissing
10 dpf	Female	WT	4	32.6325	8.75074	4.37537	76.57543	0	4
		PostnHet	5	23.77667	2.89144	1.66937	8.36043	2	3
		PostnKO	3	22.24	9.11135	5.26044	83.0167	0	3
		Spp1KO	4	20.81	5.11308	2.95204	26.1436	1	3
	Male	WT	5	33.004	4.06312	1.81708	16.50893	0	5
		PostnHet	4	16.79	8.14735	4.70387	66.3793	1	3
		PostnKO	4	21.9725	2.69436	1.34718	7.25956	0	4
		Spp1KO	3	19.09667	3.48105	2.00979	12.11773	0	3
14 dpf	Female	WT	6	54	3.13293	1.27901	9.81524	0	6
		PostnHet	7	35.255	5.31115	2.65558	28.20837	3	4
		PostnKO	6	38.248	3.69705	1.65337	13.66817	1	5
		Spp1KO	5	40.524	3.32311	1.48614	11.04308	0	5
	Male	WT	3	55.64333	2.855	1.64834	8.15103	0	3
		PostnHet	3	39.75	2.47473	1.42879	6.1243	0	3
		PostnKO	3	34.16	18.71505	10.80514	350.2531	0	3
		Spp1KO	3	38.00667	4.41749	2.55044	19.51423	0	3

ANOVAThreeWay (7/13/2023 15:09:07)

ANOVA

Overall ANOVA

	DF	Sum of Squares	Mean Square	F Value	P Value
Days Post-Fracture	1	4671.99042	4671.99042	120.20035	3.62925E-14
Gender	1	18.18553	18.18553	0.46787	0.49755
Genotype	3	2537.70452	845.90151	21.76324	8.56125E-9
Days Post-Fracture*Gender	1	14.6304	14.6304	0.37641	0.54269
Days Post-Fracture*Genotype	3	128.14198	42.71399	1.09894	0.35969
Gender*Genotype	3	26.27077	8.75692	0.2253	0.8783
Days Post-Fracture*Gender*Genotype	3	109.22157	36.40719	0.93668	0.43105
Model	15	7655.5233	510.36822	13.13069	1.2662E-11
Error	44	1710.20784	38.86836		
Corrected Total	59	9365.73114			

At the 0.05 level, the population means of **Days Post-Fracture** are **significantly** different.
At the 0.05 level, the population means of **Gender** are **not significantly** different.
At the 0.05 level, the population means of **Genotype** are **significantly** different.
At the 0.05 level, the population means of **Days Post-Fracture*Gender** are **not significantly** different.
At the 0.05 level, the population means of **Days Post-Fracture*Genotype** are **not significantly** different.
At the 0.05 level, the population means of **Gender*Genotype** are **not significantly** different.
At the 0.05 level, the population means of **Days Post-Fracture*Gender*Genotype** are **not significantly** different.

ANOVAThreeWay (7/13/2023 15:09:07)

ANOVA

Means Comparisons

Tukey Test

Days Post-Fracture

	MeanDiff	SEM	q Value	Prob	Alpha	Sig	LCL	UCL
10 dpf 14 dpf	-18.15808	1.4183	-18.10578	1.0461E-7	0.05	1	-21.01648	-15.29968

Gender

	MeanDiff	SEM	q Value	Prob	Alpha	Sig	LCL	UCL
Female Male	1.13287	1.4183	1.12961	0.42872	0.05	0	-1.72552	3.99127

Genotype

	MeanDiff	SEM	q Value	Prob	Alpha	Sig	LCL	UCL
WT PostnHet	14.92704	1.9797	10.66322	0	0.05	1	9.64122	20.21286
WT PostnKO	14.66483	1.91877	10.80857	0	0.05	1	9.54169	19.78797
WT Spp1KO	14.21063	1.95708	10.26881	0	0.05	1	8.98522	19.43603
PostnHet PostnKO	-0.26221	2.05332	-0.18059	0.99924	0.05	0	-5.74459	5.22018
PostnHet Spp1KO	-0.71642	2.08916	-0.48496	0.98594	0.05	0	-6.29449	4.86165
PostnKO Spp1KO	-0.45421	2.03152	-0.31619	0.996	0.05	0	-5.87837	4.96996

Days Post-Fracture 's

	Mean	Groups	
14 dpf	42.54875	A	
10 dpf	24.69929		B

Means that do not share a letter are significantly different.

Gender 's Grouping Le

	Mean	Groups
Female	36.05727	A
Male	31.97222	A

Means that do not share a letter are significantly different.

Genotype 's Grouping

	Mean	Groups	
WT	43.69333	A	
Spp1KO	31.16857		B
PostnKO	29.88867		B
PostnHet	29.38231		B

Means that do not share a letter are significantly different.

Sig equals 1 indicates that the difference of the means is significant at the 0.05 level.
Sig equals 0 indicates that the difference of the means is not significant at the 0.05 level.

ANOVAThreeWay (7/13/2023 15:09:07)

ANOVA

Means Comparisons

Tukey Test

Interactions 's Groupin

Days Post-Fracture	Gender	Genotype	Mean	Groups					
14 dpf	Male	WT	55.64333	A					
14 dpf	Female	WT	54	A					
14 dpf	Female	Spp1KO	40.524		B				
14 dpf	Male	PostnHet	39.75		B				
14 dpf	Female	PostnKO	38.248		B				
14 dpf	Male	Spp1KO	38.00667		B	C			
14 dpf	Female	PostnHet	35.255		B	C	D		
14 dpf	Male	PostnKO	34.16		B	C	D	E	
10 dpf	Male	WT	33.004		B	C	D	E	
10 dpf	Female	WT	32.6325		B	C	D	E	
10 dpf	Female	PostnHet	23.77667			C	D	E	F
10 dpf	Female	PostnKO	22.24			C	D	E	F
10 dpf	Male	PostnKO	21.9725				D	E	F
10 dpf	Female	Spp1KO	20.81				D	E	F
10 dpf	Male	Spp1KO	19.09667					E	F
10 dpf	Male	PostnHet	16.79						F

Means that do not share a letter are significantly different.

Sig equals 1 indicates that the difference of the means is significant at the 0.05 level.
Sig equals 0 indicates that the difference of the means is not significant at the 0.05 level.

ANOVAThreeWay (7/26/2023 15:50:24)

Descriptive Statistics

Table S5-A. CD31 IHC Callus Lumen Density
unit of measure = CD31⁺ lumens per callus mm²

Days Post-Fracture

	N	Mean	SD	SEM	Variance	Missing	NonMissing
7 dpf	27	130.19972	19.22134	3.76961	369.45976	1	26
10 dpf	27	151.18242	35.8548	7.31883	1285.56658	3	24
14 dpf	31	212.24319	57.62229	11.52446	3320.32825	6	25

Gender

	N	Mean	SD	SEM	Variance	Missing	NonMissing
Female	45	175.35521	62.93304	10.07735	3960.56733	6	39
Male	40	152.24437	37.72804	6.28801	1423.40476	4	36

Genotype

	N	Mean	SD	SEM	Variance	Missing	NonMissing
WT	21	197.91288	72.8342	17.16719	5304.82035	3	18
PostnHet	23	169.26557	49.21022	11.59896	2421.64549	5	18
PostnKO	19	143.97595	39.06402	8.9619	1525.99759	0	19
Spp1KO	22	148.74477	30.80775	6.88882	949.11767	2	20

*Days Post-Fracture*Gender*

		N	Mean	SD	SEM	Variance	Missing	NonMissing
7 dpf	Female	14	133.38424	18.4612	4.93396	340.81586	0	14
	Male	13	126.48445	20.22211	5.83762	408.93385	1	12
10 dpf	Female	14	163.2976	39.51833	11.40796	1561.69853	2	12
	Male	13	139.06723	28.39123	8.19584	806.06214	1	12
14 dpf	Female	17	231.6848	71.43556	19.81266	5103.03938	4	13
	Male	14	191.18144	27.32722	7.88869	746.77681	2	12

*Days Post-Fracture*Genotype*

		N	Mean	SD	SEM	Variance	Missing	NonMissing
7 dpf	WT	7	138.19563	12.26493	5.00714	150.4286	1	6
	PostnHet	6	136.07928	29.43284	12.0159	866.29181	0	6
	PostnKO	7	124.03854	18.75342	7.08812	351.6906	0	7
	Spp1KO	7	124.46764	12.62278	4.77096	159.33454	0	7
10 dpf	WT	6	190.60303	22.60385	9.22798	510.93419	0	6
	PostnHet	8	141.8965	9.8871	4.03639	97.75471	2	6
	PostnKO	6	136.53393	51.13624	20.87628	2614.91534	0	6
	Spp1KO	7	135.6962	13.28401	5.42317	176.46495	1	6
14 dpf	WT	8	264.93998	87.40919	35.68465	7640.36722	2	6
	PostnHet	9	229.82092	25.47025	10.39818	648.7334	3	6
	PostnKO	6	174.67826	26.50525	10.82072	702.52846	0	6
	Spp1KO	8	184.20638	19.3433	7.31108	374.1632	1	7

ANOVAThreeWay (7/26/2023 15:50:24)

Descriptive Statistics

*Gender*Genotype*

		N	Mean	SD	SEM	Variance	Missing	NonMissing
Female	WT	9	229.9528	89.4429	29.8143	8000.03325	0	9
	PostnHet	13	174.2392	54.78444	18.26148	3001.33517	4	9
	PostnKO	10	153.9674	36.44821	11.52594	1328.47209	0	10
	Spp1KO	13	151.041	35.52141	10.71011	1261.77085	2	11
Male	WT	12	165.87297	31.03157	10.34386	962.9584	3	9
	PostnHet	10	164.29193	45.70561	15.2352	2089.00318	1	9
	PostnKO	9	132.87433	40.93318	13.64439	1675.52539	0	9
	Spp1KO	9	145.93826	25.70661	8.56887	660.82995	0	9

*Days Post-Fracture*Gender*Genotype*

			N	Mean	SD	SEM	Variance	Missing	NonMissing
7 dpf	Female	WT	3	142.77	10.85537	6.26735	117.83913	0	3
		PostnHet	3	143.38773	29.10068	16.80128	846.84935	0	3
		PostnKO	4	129.10755	17.08501	8.5425	291.89748	0	4
		Spp1KO	4	123.119	13.74031	6.87016	188.79625	0	4
	Male	WT	4	133.62127	13.98062	8.07172	195.45787	1	3
		PostnHet	3	128.77083	34.0388	19.65231	1158.63984	0	3
		PostnKO	3	117.27987	22.30059	12.87525	497.31634	0	3
		Spp1KO	3	126.26583	13.64996	7.88081	186.32134	0	3
10 dpf	Female	WT	3	208.57563	16.27294	9.39519	264.80871	0	3
		PostnHet	4	137.89773	2.79236	1.61217	7.79729	1	3
		PostnKO	3	170.108	54.1571	31.26761	2932.99096	0	3
		Spp1KO	4	136.60903	14.24335	8.2234	202.87296	1	3
	Male	WT	3	172.63043	6.59422	3.80717	43.4837	0	3
		PostnHet	4	145.89527	13.73387	7.92925	188.61909	1	3
		PostnKO	3	102.95987	14.92124	8.61478	222.64354	0	3
		Spp1KO	3	134.78337	15.35544	8.86547	235.78961	0	3
14 dpf	Female	WT	3	338.51277	43.04474	24.85189	1852.84982	0	3
		PostnHet	6	241.43213	31.13817	17.97763	969.58584	3	3
		PostnKO	3	170.97327	23.3813	13.4992	546.68521	0	3
		Spp1KO	5	189.78698	25.14739	12.5737	632.39138	1	4
	Male	WT	5	191.3672	31.76799	18.34125	1009.20488	2	3
		PostnHet	3	218.2097	15.74124	9.08821	247.78661	0	3
		PostnKO	3	178.38325	34.18267	19.73537	1168.45502	0	3
		Spp1KO	3	176.76559	5.34494	3.0859	28.56842	0	3

ANOVAThreeWay (7/26/2023 15:50:24)

ANOVA

Overall ANOVA

	DF	Sum of Squares	Mean Square	F Value	P Value
Days Post-Fracture	2	92481.97691	46240.98845	80.7955	1.55112E-16
Gender	1	12030.58111	12030.58111	21.02068	2.97339E-5
Genotype	3	33209.79972	11069.93324	19.34217	1.65228E-8
Days Post-Fracture*Gender	2	4062.29047	2031.14524	3.54896	0.03605
Days Post-Fracture*Genotype	6	13552.0059	2258.66765	3.9465	0.00257
Gender*Genotype	3	10083.85867	3361.28622	5.87308	0.00159
Days Post-Fracture*Gender*Genotype	6	17712.94061	2952.15677	5.15822	3.28983E-4
Model	23	181130.88843	7875.25602	13.7602	1.18987E-14
Error	51	29188.38644	572.3213		
Corrected Total	74	210319.27488			

At the 0.05 level, the population means of **Days Post-Fracture** are **significantly** different.
At the 0.05 level, the population means of **Gender** are **significantly** different.
At the 0.05 level, the population means of **Genotype** are **significantly** different.
At the 0.05 level, the population means of **Days Post-Fracture*Gender** are **significantly** different.
At the 0.05 level, the population means of **Days Post-Fracture*Genotype** are **significantly** different.
At the 0.05 level, the population means of **Gender*Genotype** are **significantly** different.
At the 0.05 level, the population means of **Days Post-Fracture*Gender*Genotype** are **significantly** different.

ANOVAThreeWay (7/26/2023 15:50:24)

ANOVA

Means Comparisons

Tukey Test

Days Post-Fracture

	MeanDiff	SEM	q Value	Prob	Alpha	Sig	LCL	UCL
7 dpf 10 dpf	-20.64216	5.60518	-5.20811	0.00159	0.05	1	-34.17273	-7.11158
7 dpf 14 dpf	-82.6386	5.55979	-21.02032	0	0.05	1	-96.05961	-69.21759
10 dpf 14 dpf	-61.99644	5.6502	-15.51736	0	0.05	1	-75.6357	-48.35719

Gender

	MeanDiff	SEM	q Value	Prob	Alpha	Sig	LCL	UCL
Female Male	25.44561	4.57661	7.86292	1.04391E-6	0.05	1	16.25767	34.63355

Genotype

	MeanDiff	SEM	q Value	Prob	Alpha	Sig	LCL	UCL
WT PostnHet	28.64732	6.57587	6.16092	3.63308E-4	0.05	1	11.18307	46.11156
WT PostnKO	53.11092	6.50701	11.54296	0	0.05	1	35.82955	70.39229
WT Spp1KO	50.02458	6.43741	10.98973	0	0.05	1	32.92805	67.12112
PostnHet PostnKO	24.4636	6.50701	5.31684	0.00241	0.05	1	7.18223	41.74497
PostnHet Spp1KO	21.37727	6.43741	4.6963	0.00876	0.05	1	4.28073	38.4738
PostnKO Spp1KO	-3.08633	6.36706	-0.68552	0.96215	0.05	0	-19.99602	13.82335

Days Post-Fracture 's

	Mean	Groups		
14 dpf	212.24319	A		
10 dpf	151.18242		B	
7 dpf	130.19972			C

Means that do not share a letter are significantly different.

Gender 's Grouping L

	Mean	Groups	
Female	175.35521	A	
Male	152.24437		B

Means that do not share a letter are significantly different.

Genotype 's Grouping

	Mean	Groups		
WT	197.91288	A		
PostnHet	169.26557		B	
Spp1KO	148.74477			C
PostnKO	143.97595			C

Means that do not share a letter are significantly different.

Sig equals 1 indicates that the difference of the means is significant at the 0.05 level.
Sig equals 0 indicates that the difference of the means is not significant at the 0.05 level.

ANOVAThreeWay (7/26/2023 15:50:24)

ANOVA

Means Comparisons

Tukey Test

Interactions 's Groupi

Days Post-Fracture	Gender	Genotype	Mean	Groups					
14 dpf	Female	WT	338.51277	A					
14 dpf	Female	PostnHet	241.43213		B				
14 dpf	Male	PostnHet	218.2097		B	C			
10 dpf	Female	WT	208.57563		B	C			
14 dpf	Male	WT	191.3672		B	C	D		
14 dpf	Female	Spp1KO	189.78698		B	C	D		
14 dpf	Male	PostnKO	178.38325			C	D	E	
14 dpf	Male	Spp1KO	176.76559			C	D	E	
10 dpf	Male	WT	172.63043			C	D	E	
14 dpf	Female	PostnKO	170.97327			C	D	E	
10 dpf	Female	PostnKO	170.108			C	D	E	
10 dpf	Male	PostnHet	145.89527				D	E	F
7 dpf	Female	PostnHet	143.38773				D	E	F
7 dpf	Female	WT	142.77				D	E	F
10 dpf	Female	PostnHet	137.89773				D	E	F
10 dpf	Female	Spp1KO	136.60903				D	E	F
10 dpf	Male	Spp1KO	134.78337				D	E	F
7 dpf	Male	WT	133.62127				D	E	F
7 dpf	Female	PostnKO	129.10755					E	F
7 dpf	Male	PostnHet	128.77083					E	F
7 dpf	Male	Spp1KO	126.26583					E	F
7 dpf	Female	Spp1KO	123.119					E	F
7 dpf	Male	PostnKO	117.27987					E	F
10 dpf	Male	PostnKO	102.95987						F

Means that do not share a letter are significantly different.

Sig equals 1 indicates that the difference of the means is significant at the 0.05 level.
Sig equals 0 indicates that the difference of the means is not significant at the 0.05 level.

ANOVAThreeWay (7/27/2023 15:35:24)

Descriptive Statistics

Table S5-B. CD31 IHC Average Lumen Area

unit of measure = µm²

Days Post-Fracture

	N	Mean	SD	SEM	Variance	Missing	NonMissing
7 dpf	27	731.49683	152.5693	29.9213	23277.39053	1	26
10 dpf	27	807.89417	160.77048	32.81714	25847.14836	3	24
14 dpf	31	991.94196	194.43064	38.88613	37803.27314	6	25

Gender

	N	Mean	SD	SEM	Variance	Missing	NonMissing
Female	45	814.8592	187.78143	30.06909	35261.86462	6	39
Male	40	872.98383	213.00068	35.50011	45369.28918	4	36

Genotype

	N	Mean	SD	SEM	Variance	Missing	NonMissing
WT	21	821.64566	194.14442	45.76028	37692.05466	3	18
PostnHet	23	849.76763	195.84744	46.16169	38356.22108	5	18
PostnKO	19	804.47847	247.2753	56.72885	61145.07573	0	19
Spp1KO	22	891.81982	164.28457	36.73515	26989.41907	2	20

*Days Post-Fracture*Gender*

		N	Mean	SD	SEM	Variance	Missing	NonMissing
7 dpf	Female	14	726.37441	180.57766	48.26141	32608.29215	0	14
	Male	13	737.47299	119.55639	34.51296	14293.73123	1	12
10 dpf	Female	14	819.17127	149.15502	43.05735	22247.21967	2	12
	Male	13	796.61708	177.5369	51.25049	31519.34968	1	12
14 dpf	Female	17	906.17015	194.31434	53.8931	37758.062	4	13
	Male	14	1084.86142	152.23638	43.94686	23175.91469	2	12

*Days Post-Fracture*Genotype*

		N	Mean	SD	SEM	Variance	Missing	NonMissing
7 dpf	WT	7	887.41245	83.64084	34.14623	6995.79035	1	6
	PostnHet	6	650.10004	87.43561	35.69544	7644.98647	0	6
	PostnKO	7	577.04779	111.41147	42.10958	12412.51598	0	7
	Spp1KO	7	822.07258	63.55544	24.0217	4039.29415	0	7
10 dpf	WT	6	705.64782	134.69052	54.98717	18141.5352	0	6
	PostnHet	8	823.11588	36.6418	14.95895	1342.62171	2	6
	PostnKO	6	901.27803	244.2417	99.71126	59654.00975	0	6
	Spp1KO	7	801.53496	128.14957	52.31684	16422.31357	1	6
14 dpf	WT	8	871.87671	280.44776	114.49232	78650.94615	2	6
	PostnHet	9	1076.08695	106.14174	43.33218	11266.06918	3	6
	PostnKO	6	973.01471	166.82056	68.10421	27829.10087	0	6
	Spp1KO	8	1038.95409	169.28836	63.98498	28658.54793	1	7

ANOVAThreeWay (7/27/2023 15:35:24)

Descriptive Statistics

*Gender*Genotype*

		N	Mean	SD	SEM	Variance	Missing	NonMissing
Female	WT	9	788.0904	177.72651	59.24217	31586.71135	0	9
	PostnHet	13	839.34947	201.10723	67.03574	40444.11851	4	9
	PostnKO	10	754.99151	252.04824	79.70465	63528.31699	0	10
	Spp1KO	13	871.14864	104.59798	31.53748	10940.73748	2	11
Male	WT	12	855.20092	214.41899	71.473	45975.5045	3	9
	PostnHet	10	860.18578	202.03624	67.34541	40818.64072	1	9
	PostnKO	9	859.46399	244.2223	81.40743	59644.53184	0	9
	Spp1KO	9	917.08461	221.62652	73.87551	49118.31574	0	9

*Days Post-Fracture*Gender*Genotype*

			N	Mean	SD	SEM	Variance	Missing	NonMissing
7 dpf	Female	WT	3	935.49987	91.47209	52.81144	8367.14348	0	3
		PostnHet	3	625.43857	123.3219	71.19993	15208.29068	0	3
		PostnKO	4	541.76256	84.69679	42.3484	7173.54649	0	4
		Spp1KO	4	829.84405	69.0936	34.5468	4773.92575	0	4
	Male	WT	4	839.32503	46.7454	26.98847	2185.13258	1	3
		PostnHet	3	674.76152	45.60275	26.32876	2079.61108	0	3
		PostnKO	3	624.09477	143.76024	83.00001	20667.00596	0	3
		Spp1KO	3	811.71063	68.37505	39.47635	4675.14743	0	3
10 dpf	Female	WT	3	784.86688	160.47909	92.65265	25753.53928	0	3
		PostnHet	4	847.11364	27.54061	15.90058	758.48516	1	3
		PostnKO	3	804.75335	262.2891	151.43268	68795.57358	0	3
		Spp1KO	4	839.9512	152.17173	87.85639	23156.23588	1	3
	Male	WT	3	626.42876	27.80865	16.05533	773.32128	0	3
		PostnHet	4	799.11812	29.5024	17.03322	870.3919	1	3
		PostnKO	3	997.80271	228.88514	132.1469	52388.4075	0	3
		Spp1KO	3	763.11871	116.06948	67.01274	13472.12343	0	3
14 dpf	Female	WT	3	643.90445	168.61751	97.35136	28431.86431	0	3
		PostnHet	6	1045.49619	115.48923	66.67774	13337.76338	3	3
		PostnKO	3	989.53494	170.75734	98.5868	29158.06878	0	3
		Spp1KO	5	935.8513	86.18444	43.09222	7427.75729	1	4
	Male	WT	5	1099.84898	110.82165	63.98291	12281.43701	2	3
		PostnHet	3	1106.67771	109.63588	63.2983	12020.02526	0	3
		PostnKO	3	956.49448	198.98726	114.88535	39595.92929	0	3
		Spp1KO	3	1176.42449	158.82844	91.69964	25226.4722	0	3

ANOVAThreeWay (7/27/2023 15:35:24)

Descriptive Statistics

ANOVA

Overall ANOVA

	DF	Sum of Squares	Mean Square	F Value	P Value
Days Post-Fracture	2	893165.01694	446582.50847	26.59068	1.22855E-8
Gender	1	54816.61795	54816.61795	3.26392	0.07672
Genotype	3	67563.94088	22521.31363	1.34098	0.2714
Days Post-Fracture*Gender	2	151097.63999	75548.82	4.49837	0.01588
Days Post-Fracture*Genotype	6	588600.04838	98100.00806	5.84113	1.08694E-4
Gender*Genotype	3	9232.90631	3077.63544	0.18325	0.9073
Days Post-Fracture*Gender*Genotype	6	332249.55343	55374.92557	3.29717	0.00809
Model	23	2134591.34273	92808.31925	5.52605	1.92759E-7
Error	51	856529.62744	16794.69858		
Corrected Total	74	2991120.97017			

At the 0.05 level, the population means of **Days Post-Fracture** are **significantly** different.
At the 0.05 level, the population means of **Gender** are **not significantly** different.
At the 0.05 level, the population means of **Genotype** are **not significantly** different.
At the 0.05 level, the population means of **Days Post-Fracture*Gender** are **significantly** different.
At the 0.05 level, the population means of **Days Post-Fracture*Genotype** are **significantly** different.
At the 0.05 level, the population means of **Gender*Genotype** are **not significantly** different.
At the 0.05 level, the population means of **Days Post-Fracture*Gender*Genotype** are **significantly** different.

ANOVAThreeWay (7/27/2023 15:35:24)

Descriptive Statistics

ANOVA

Means Comparisons

Tukey Test

Days Post-Fracture

	MeanDiff	SEM	q Value	Prob	Alpha	Sig	LCL	UCL
7 dpf 10 dpf	-72.58955	30.36378	-3.38091	0.05277	0.05	0	-145.88591	0.70681
7 dpf 14 dpf	-258.97444	30.11791	-12.16038	0	0.05	1	-331.6773	-186.27158
10 dpf 14 dpf	-186.38489	30.60767	-8.61183	4.11683E-7	0.05	1	-260.26999	-112.4998

Gender

	MeanDiff	SEM	q Value	Prob	Alpha	Sig	LCL	UCL
Female Male	-54.31574	24.79192	-3.09835	0.03306	0.05	1	-104.08765	-4.54383

Genotype

	MeanDiff	SEM	q Value	Prob	Alpha	Sig	LCL	UCL
WT PostnHet	-28.12196	35.62208	-1.11646	0.8589	0.05	0	-122.72738	66.48345
WT PostnKO	2.57186	35.24907	0.10318	0.99986	0.05	0	-91.04289	96.18661
WT Spp1KO	-71.17107	34.87206	-2.8863	0.18674	0.05	0	-163.78457	21.44243
PostnHet PostnKO	30.69382	35.24907	1.23145	0.81987	0.05	0	-62.92093	124.30858
PostnHet Spp1KO	-43.0491	34.87206	-1.74583	0.60815	0.05	0	-135.6626	49.56439
PostnKO Spp1KO	-73.74293	34.49093	-3.02364	0.15499	0.05	0	-165.34423	17.85837

Days Post-Fracture 's

	Mean	Groups	
14 dpf	991.94196	A	
10 dpf	807.89417		B
7 dpf	731.49683		B

Means that do not share a letter are significantly different.

Gender 's Grouping Le

	Mean	Groups	
Male	872.98383	A	
Female	814.8592		B

Means that do not share a letter are significantly different.

Genotype 's Grouping

	Mean	Groups
Spp1KO	891.81982	A
PostnHet	849.76763	A
WT	821.64566	A
PostnKO	804.47847	A

Means that do not share a letter are significantly different.

Sig equals 1 indicates that the difference of the means is significant at the 0.05 level.
Sig equals 0 indicates that the difference of the means is not significant at the 0.05 level.

Descriptive Statistics

ANOVA

Means Comparisons

Tukey Test

Interactions 's Groupin

Days Post-Fracture	Gender	Genotype	Mean	Groups					
14 dpf	Male	Spp1KO	1176.42449	A					
14 dpf	Male	PostnHet	1106.67771	A	B				
14 dpf	Male	WT	1099.84898	A	B				
14 dpf	Female	PostnHet	1045.49619	A	B	C			
10 dpf	Male	PostnKO	997.80271	A	B	C	D		
14 dpf	Female	PostnKO	989.53494	A	B	C	D		
14 dpf	Male	PostnKO	956.49448	A	B	C	D	E	
14 dpf	Female	Spp1KO	935.8513	A	B	C	D	E	
7 dpf	Female	WT	935.49987	A	B	C	D	E	
10 dpf	Female	PostnHet	847.11364	A	B	C	D	E	F
10 dpf	Female	Spp1KO	839.9512		B	C	D	E	F
7 dpf	Male	WT	839.32503		B	C	D	E	F
7 dpf	Female	Spp1KO	829.84405		B	C	D	E	F
7 dpf	Male	Spp1KO	811.71063		B	C	D	E	F
10 dpf	Female	PostnKO	804.75335		B	C	D	E	F
10 dpf	Male	PostnHet	799.11812		B	C	D	E	F
10 dpf	Female	WT	784.86688		B	C	D	E	F
10 dpf	Male	Spp1KO	763.11871			C	D	E	F
7 dpf	Male	PostnHet	674.76152				D	E	F
14 dpf	Female	WT	643.90445					E	F
10 dpf	Male	WT	626.42876					E	F
7 dpf	Female	PostnHet	625.43857					E	F
7 dpf	Male	PostnKO	624.09477					E	F
7 dpf	Female	PostnKO	541.76256						F

Means that do not share a letter are significantly different.

Sig equals 1 indicates that the difference of the means is significant at the 0.05 level.
Sig equals 0 indicates that the difference of the means is not significant at the 0.05 level.

ANOVAThreeWay (7/13/2023 15:51:00)

Descriptive Statistics

Table S6-A. F4-80$^+$ Macrophage Density

unit of measure = F4-80$^+$ cells per mm^2 of callus

Days Post-Fracture

	N	Mean	SD	SEM	Variance	Missing	NonMissing
7 dpf	27	131.18255	28.0877	5.50845	788.91906	1	26
10 dpf	26	150.15403	32.94495	6.72486	1085.36946	2	24
14 dpf	33	182.53702	22.2582	4.45164	495.42746	8	25

Gender

	N	Mean	SD	SEM	Variance	Missing	NonMissing
Female	43	153.67509	36.13957	5.78696	1306.06882	4	39
Male	43	155.12611	34.28741	5.71457	1175.62635	7	36

Genotype

	N	Mean	SD	SEM	Variance	Missing	NonMissing
WT	23	185.58452	20.41972	4.81297	416.96483	5	18
PostnHet	19	159.74614	25.42228	5.99209	646.29243	1	18
PostnKO	21	133.93221	27.95579	6.4135	781.52616	2	19
Spp1KO	23	140.86023	39.08761	8.74025	1527.84095	3	20

*Days Post-Fracture*Gender*

		N	Mean	SD	SEM	Variance	Missing	NonMissing
7 dpf	Female	14	130.62327	28.56579	7.63453	816.00456	0	14
	Male	13	131.83503	28.77093	8.30545	827.76631	1	12
10 dpf	Female	12	149.12283	35.68576	10.30159	1273.47369	0	12
	Male	14	151.18524	31.52166	9.09952	993.61505	2	12
14 dpf	Female	17	182.70221	23.04489	6.3915	531.06689	4	13
	Male	16	182.35807	22.39464	6.46477	501.51976	4	12

*Days Post-Fracture*Genotype*

		N	Mean	SD	SEM	Variance	Missing	NonMissing
7 dpf	WT	6	163.44408	12.83464	5.23972	164.72795	0	6
	PostnHet	6	141.45213	15.76132	6.43453	248.41924	0	6
	PostnKO	8	119.93291	16.70176	6.31267	278.94893	1	7
	Spp1KO	7	105.97693	25.07581	9.47777	628.79638	0	7
10 dpf	WT	6	189.5085	4.92987	2.01261	24.3036	0	6
	PostnHet	6	157.47763	19.15642	7.82058	366.96842	0	6
	PostnKO	7	117.46465	13.03713	5.32239	169.96688	1	6
	Spp1KO	7	136.16535	31.48706	12.85454	991.43476	1	6
14 dpf	WT	11	203.80098	14.95746	6.10636	223.72555	5	6
	PostnHet	7	180.30867	25.82335	10.54234	666.84542	1	6
	PostnKO	6	166.73228	20.4447	8.34651	417.98578	0	6
	Spp1KO	9	179.7677	13.24338	5.00553	175.38716	2	7

ANOVAThreeWay (7/13/2023 15:51:00)

Descriptive Statistics

*Gender*Genotype*

		N	Mean	SD	SEM	Variance	Missing	NonMissing
Female	WT	12	187.71659	20.69524	6.89841	428.293	3	9
	PostnHet	9	158.68006	26.581	8.86033	706.54981	0	9
	PostnKO	10	134.23551	31.45407	9.94665	989.35858	0	10
	Spp1KO	12	139.40032	38.69412	11.66672	1497.23524	1	11
Male	WT	11	183.45246	21.15489	7.05163	447.52942	2	9
	PostnHet	10	160.81223	25.77333	8.59111	664.26437	1	9
	PostnKO	11	133.59521	25.40005	8.46668	645.16271	2	9
	Spp1KO	11	142.64456	41.83976	13.94659	1750.5658	2	9

*Days Post-Fracture*Gender*Genotype*

			N	Mean	SD	SEM	Variance	Missing	NonMissing
7 dpf	Female	WT	3	166.96477	7.13601	4.11998	50.92262	0	3
		PostnHet	3	138.09943	13.32104	7.6909	177.45004	0	3
		PostnKO	4	121.93405	10.60028	5.30014	112.36597	0	4
		Spp1KO	4	106.44925	31.85844	15.92922	1014.96025	0	4
	Male	WT	3	159.9234	17.99199	10.38768	323.71162	0	3
		PostnHet	3	144.80483	20.2454	11.68869	409.87626	0	3
		PostnKO	4	117.26473	25.48745	14.71518	649.60996	1	3
		Spp1KO	3	105.34717	19.05014	10.9986	362.90768	0	3
10 dpf	Female	WT	3	191.67027	5.53497	3.19562	30.63591	0	3
		PostnHet	3	167.3227	21.20625	12.24343	449.70495	0	3
		PostnKO	3	107.55627	6.29204	3.63271	39.58974	0	3
		Spp1KO	3	129.94207	11.33022	6.54151	128.37393	0	3
	Male	WT	3	187.34673	4.0129	2.31685	16.10339	0	3
		PostnHet	3	147.63257	13.30188	7.67985	176.94009	0	3
		PostnKO	4	127.37303	9.52887	5.50149	90.79927	1	3
		Spp1KO	4	142.38863	47.26548	27.28874	2234.0252	1	3
14 dpf	Female	WT	6	204.51473	23.21513	13.40326	538.94242	3	3
		PostnHet	3	170.61803	35.18359	20.31326	1237.88532	0	3
		PostnKO	3	177.3167	9.46925	5.46707	89.66665	0	3
		Spp1KO	5	179.44508	14.66204	7.33102	214.97549	1	4
	Male	WT	5	203.08723	4.34087	2.5062	18.84313	2	3
		PostnHet	4	189.9993	12.14509	7.01197	147.50312	1	3
		PostnKO	3	156.14787	24.88389	14.36672	619.20818	0	3
		Spp1KO	4	180.19787	14.25526	8.23028	203.21249	1	3

ANOVAThreeWay (7/13/2023 15:51:00)

ANOVA

Overall ANOVA

	DF	Sum of Squares	Mean Square	F Value	P Value
Days Post-Fracture	2	32372.32275	16186.16137	41.2361	2.21602E-11
Gender	1	0.01321	0.01321	3.36561E-5	0.99539
Genotype	3	29104.54352	9701.51451	24.71572	5.16064E-10
Days Post-Fracture*Gender	2	42.70951	21.35476	0.0544	0.9471
Days Post-Fracture*Genotype	6	4790.94216	798.49036	2.03425	0.0779
Gender*Genotype	3	200.98888	66.99629	0.17068	0.91571
Days Post-Fracture*Gender*Genotype	6	2615.76034	435.96006	1.11066	0.36924
Model	23	70798.2227	3078.1836	7.84202	5.85568E-10
Error	51	20018.72911	392.5241		
Corrected Total	74	90816.95181			

At the 0.05 level, the population means of **Days Post-Fracture** are **significantly** different.
At the 0.05 level, the population means of **Gender** are **not significantly** different.
At the 0.05 level, the population means of **Genotype** are **significantly** different.
At the 0.05 level, the population means of **Days Post-Fracture*Gender** are **not significantly** different.
At the 0.05 level, the population means of **Days Post-Fracture*Genotype** are **not significantly** different.
At the 0.05 level, the population means of **Gender*Genotype** are **not significantly** different.
At the 0.05 level, the population means of **Days Post-Fracture*Gender*Genotype** are **not significantly** different.

ANOVAThreeWay (7/13/2023 15:51:00)

ANOVA

Means Comparisons

Tukey Test

Days Post-Fracture

	MeanDiff	SEM	q Value	Prob	Alpha	Sig	LCL	UCL
7 dpf 10 dpf	-17.55558	4.64198	-5.34844	0.00117	0.05	1	-28.76103	-6.35012
7 dpf 14 dpf	-50.0674	4.60439	-15.37794	0	0.05	1	-61.18212	-38.95268
10 dpf 14 dpf	-32.51182	4.67926	-9.82605	0	0.05	1	-43.80728	-21.21636

Gender

	MeanDiff	SEM	q Value	Prob	Alpha	Sig	LCL	UCL
Female Male	0.02666	3.79016	0.00995	0.99441	0.05	0	-7.5824	7.63573

Genotype

	MeanDiff	SEM	q Value	Prob	Alpha	Sig	LCL	UCL
WT PostnHet	25.83838	5.44586	6.70987	9.94941E-5	0.05	1	11.37522	40.30153
WT PostnKO	50.98575	5.38883	13.3804	3.48492E-9	0.05	1	36.67404	65.29745
WT Spp1KO	44.95618	5.3312	11.92559	0	0.05	1	30.79755	59.11481
PostnHet PostnKO	25.14737	5.38883	6.59953	1.29518E-4	0.05	1	10.83567	39.45907
PostnHet Spp1KO	19.1178	5.3312	5.0714	0.00406	0.05	1	4.95917	33.27643
PostnKO Spp1KO	-6.02957	5.27293	-1.61715	0.66463	0.05	0	-20.03346	7.97432

Days Post-Fracture 's

	Mean	Groups		
14 dpf	182.53702	A		
10 dpf	150.15403		B	
7 dpf	131.18255			C

Means that do not share a letter are significantly different.

Gender 's Grouping Le

	Mean	Groups
Male	155.12611	A
Female	153.67509	A

Means that do not share a letter are significantly different.

Genotype 's Grouping

	Mean	Groups		
WT	185.58452	A		
PostnHet	159.74614		B	
Spp1KO	140.86023			C
PostnKO	133.93221			C

Means that do not share a letter are significantly different.

Sig equals 1 indicates that the difference of the means is significant at the 0.05 level.
Sig equals 0 indicates that the difference of the means is not significant at the 0.05 level.

ANOVAThreeWay (7/13/2023 15:51:00)

ANOVA

Means Comparisons

Tukey Test

Interactions 's Groupin

Days Post-Fracture	Gender	Genotype	Mean	Groups							
14 dpf	Female	WT	204.51473	A							
14 dpf	Male	WT	203.08723	A							
10 dpf	Female	WT	191.67027	A	B						
14 dpf	Male	PostnHet	189.9993	A	B						
10 dpf	Male	WT	187.34673	A	B	C					
14 dpf	Male	Spp1KO	180.19787	A	B	C	D				
14 dpf	Female	Spp1KO	179.44508	A	B	C					
14 dpf	Female	PostnKO	177.3167	A	B	C	D	E			
14 dpf	Female	PostnHet	170.61803	A	B	C	D	E			
10 dpf	Female	PostnHet	167.3227	A	B	C	D	E	F		
7 dpf	Female	WT	166.96477	A	B	C	D	E	F		
7 dpf	Male	WT	159.9234	A	B	C	D	E	F		
14 dpf	Male	PostnKO	156.14787	A	B	C	D	E	F	G	
10 dpf	Male	PostnHet	147.63257		B	C	D	E	F	G	H
7 dpf	Male	PostnHet	144.80483		B	C	D	E	F	G	H
10 dpf	Male	Spp1KO	142.38863		B	C	D	E	F	G	H
7 dpf	Female	PostnHet	138.09943			C	D	E	F	G	H
10 dpf	Female	Spp1KO	129.94207				D	E	F	G	H
10 dpf	Male	PostnKO	127.37303					E	F	G	H
7 dpf	Female	PostnKO	121.93405						F	G	H
7 dpf	Male	PostnKO	117.26473						F	G	H
10 dpf	Female	PostnKO	107.55627							G	H
7 dpf	Female	Spp1KO	106.44925								H
7 dpf	Male	Spp1KO	105.34717							G	H

Means that do not share a letter are significantly different.

Sig equals 1 indicates that the difference of the means is significant at the 0.05 level.
Sig equals 0 indicates that the difference of the means is not significant at the 0.05 level.

ANOVAThreeWay (7/13/2023 12:59:31)

Descriptive Statistics

Table S6-B. CTSK+ Osteoclast Density

unit of measure = CTSK+ cells per mm² of callus

Days Post-Fracture

	N	Mean	SD	SEM	Variance	Missing	NonMissing
10 dpf	31	11.3586	7.88916	1.57783	62.23877	6	25
14 dpf	28	38.48762	11.89574	2.37915	141.50859	3	25
21 dpf	33	43.04332	9.02907	1.73764	81.52406	6	27

Gender

	N	Mean	SD	SEM	Variance	Missing	NonMissing
Female	45	32.63151	16.86609	2.63404	284.46495	4	41
Male	47	29.73425	17.25978	2.87663	297.90005	11	36

Genotype

	N	Mean	SD	SEM	Variance	Missing	NonMissing
WT	26	42.80354	17.49514	4.01366	306.08002	7	19
PostnHet	20	29.88027	16.55741	3.79853	274.14779	1	19
PostnKO	21	23.65171	13.17941	3.02356	173.69679	2	19
Spp1KO	25	28.89751	15.58666	3.48528	242.94408	5	20

*Days Post-Fracture*Gender*

		N	Mean	SD	SEM	Variance	Missing	NonMissing
10 dpf	Female	14	11.49452	5.54467	1.53781	30.74332	1	13
	Male	17	11.21135	10.1099	2.91848	102.21003	5	12
14 dpf	Female	14	43.10417	11.14113	3.08999	124.12476	1	13
	Male	14	33.48635	10.99377	3.17363	120.86307	2	12
21 dpf	Female	17	41.87394	8.47267	2.18763	71.78606	2	15
	Male	16	44.50504	9.85564	2.84508	97.13359	4	12

*Days Post-Fracture*Genotype*

		N	Mean	SD	SEM	Variance	Missing	NonMissing
10 dpf	WT	9	21.21533	10.80285	4.41024	116.70155	3	6
	PostnHet	6	8.59807	2.90957	1.18783	8.46561	0	6
	PostnKO	8	6.5099	2.90918	1.18767	8.46332	2	6
	Spp1KO	8	9.43216	2.23182	0.84355	4.981	1	7
14 dpf	WT	7	52.12268	9.12287	3.7244	83.22676	1	6
	PostnHet	6	37.61133	12.50885	5.10672	156.47127	0	6
	PostnKO	6	28.79508	7.13312	2.91209	50.88146	0	6
	Spp1KO	9	35.8594	5.93278	2.24238	35.19789	2	7
21 dpf	WT	10	53.31989	8.34927	3.15573	69.71026	3	7
	PostnHet	8	41.49553	3.85934	1.45869	14.8945	1	7
	PostnKO	7	33.93607	5.28624	1.99801	27.94434	0	7
	Spp1KO	8	43.48487	4.61613	1.88453	21.30863	2	6

ANOVAThreeWay (7/13/2023 12:59:31)

Descriptive Statistics

*Gender*Genotype*

		N	Mean	SD	SEM	Variance	Missing	NonMissing
Female	WT	11	42.55251	18.32285	5.7942	335.72701	1	10
	PostnHet	11	33.32437	17.05696	5.39388	290.93973	1	10
	PostnKO	10	25.63422	13.59516	4.29917	184.82848	0	10
	Spp1KO	13	29.34373	15.74024	4.74586	247.75529	2	11
Male	WT	15	43.08247	17.63011	5.8767	310.82087	6	9
	PostnHet	9	26.05349	16.06933	5.35644	258.22325	0	9
	PostnKO	11	21.44891	13.13446	4.37815	172.51394	2	9
	Spp1KO	12	28.35212	16.33064	5.44355	266.68967	3	9

*Days Post-Fracture*Gender*Genotype*

			N	Mean	SD	SEM	Variance	Missing	NonMissing
10 dpf	Female	WT	3	18.4878	7.40638	4.27608	54.85447	0	3
		PostnHet	3	10.16607	1.02053	0.58921	1.04149	0	3
		PostnKO	3	7.05433	3.99164	2.30457	15.93316	0	3
		Spp1KO	5	10.57603	1.89112	0.94556	3.57634	1	4
	Male	WT	6	23.94287	14.64859	8.45737	214.58108	3	3
		PostnHet	3	7.03007	3.57025	2.06128	12.74666	0	3
		PostnKO	5	5.96547	2.08229	1.20221	4.33593	2	3
		Spp1KO	3	7.907	1.86346	1.07587	3.47247	0	3
14 dpf	Female	WT	3	57.10103	4.73492	2.73371	22.41949	0	3
		PostnHet	3	45.82513	11.59666	6.69534	134.48259	0	3
		PostnKO	3	34.2609	3.0767	1.77633	9.46606	0	3
		Spp1KO	5	37.19825	7.58691	3.79345	57.56119	1	4
	Male	WT	4	47.14433	10.54967	6.09085	111.29552	1	3
		PostnHet	3	29.39753	7.36859	4.25425	54.29605	0	3
		PostnKO	3	23.32927	5.30209	3.06116	28.11214	0	3
		Spp1KO	4	34.07427	3.29952	1.90498	10.8868	1	3
21 dpf	Female	WT	5	49.68965	9.84768	4.92384	96.97689	1	4
		PostnHet	5	41.31753	2.16941	1.08471	4.70635	1	4
		PostnKO	4	33.09913	6.60463	3.30231	43.62109	0	4
		Spp1KO	3	43.89463	1.27926	0.73858	1.6365	0	3
	Male	WT	5	58.1602	1.47148	0.84956	2.16526	2	3
		PostnHet	3	41.73287	6.12177	3.53441	37.47613	0	3
		PostnKO	3	35.052	3.89005	2.24592	15.13247	0	3
		Spp1KO	5	43.0751	7.15062	4.12841	51.13134	2	3

ANOVAThreeWay (7/13/2023 12:59:31)

Descriptive Statistics

ANOVA

Overall ANOVA

	DF	Sum of Squares	Mean Square	F Value	P Value
Days Post-Fracture	2	14901.9654	7450.9827	180.29944	2.25921E-24
Gender	1	133.84988	133.84988	3.23891	0.0776
Genotype	3	3718.88449	1239.62816	29.99662	1.78605E-11
Days Post-Fracture*Gender	2	552.85801	276.429	6.68905	0.00257
Days Post-Fracture*Genotype	6	177.34735	29.55789	0.71524	0.63892
Gender*Genotype	3	142.86052	47.62017	1.15232	0.33665
Days Post-Fracture*Gender*Genotype	6	156.97697	26.16283	0.63309	0.70311
Model	23	19775.74926	859.81519	20.80587	5.62734E-19
Error	53	2190.25679	41.3256		
Corrected Total	76	21966.00605			

At the 0.05 level, the population means of **Days Post-Fracture** are **significantly** different.
At the 0.05 level, the population means of **Gender** are **not significantly** different.
At the 0.05 level, the population means of **Genotype** are **significantly** different.
At the 0.05 level, the population means of **Days Post-Fracture*Gender** are **significantly** different.
At the 0.05 level, the population means of **Days Post-Fracture*Genotype** are **not significantly** different.
At the 0.05 level, the population means of **Gender*Genotype** are **not significantly** different.
At the 0.05 level, the population means of **Days Post-Fracture*Gender*Genotype** are **not significantly** different.

ANOVA
Means Comparisons
Tukey Test
Days Post-Fracture

	MeanDiff	SEM	q Value	Prob	Alpha	Sig	LCL	UCL
10 dpf 14 dpf	-27.15014	1.51537	-25.33784	0	0.05	1	-30.80402	-23.49625
10 dpf 21 dpf	-31.86143	1.49072	-30.22617	0	0.05	1	-35.4559	-28.26696
14 dpf 21 dpf	-4.7113	1.49072	-4.46949	0.00723	0.05	1	-8.30577	-1.11683

Gender

	MeanDiff	SEM	q Value	Prob	Alpha	Sig	LCL	UCL
Female Male	2.65496	1.22391	3.06776	0.03457	0.05	1	0.2001	5.10982

Genotype

	MeanDiff	SEM	q Value	Prob	Alpha	Sig	LCL	UCL
WT PostnHet	13.17612	1.74036	10.70689	0	0.05	1	8.55989	17.79235
WT PostnKO	19.29413	1.74036	15.67837	3.8972E-7	0.05	1	14.6779	23.91036
WT Spp1KO	12.96677	1.72134	10.6532	0	0.05	1	8.40099	17.53255
PostnHet PostnKO	6.11802	1.74036	4.97149	0.00489	0.05	1	1.50179	10.73425
PostnHet Spp1KO	-0.20935	1.72134	-0.17199	0.99935	0.05	0	-4.77512	4.35643
PostnKO Spp1KO	-6.32736	1.72134	-5.19842	0.00302	0.05	1	-10.89314	-1.76159

Days Post-Fracture 's

	Mean	Groups		
21 dpf	43.04332	A		
14 dpf	38.48762		B	
10 dpf	11.3586			C

Means that do not share a letter are significantly different.

Gender 's Grouping Le

	Mean	Groups	
Female	32.63151	A	
Male	29.73425		B

Means that do not share a letter are significantly different.

Genotype 's Grouping

	Mean	Groups		
WT	42.80354	A		
PostnHet	29.88027		B	
Spp1KO	28.89751		B	
PostnKO	23.65171			C

Means that do not share a letter are significantly different.

Sig equals 1 indicates that the difference of the means is significant at the 0.05 level.
Sig equals 0 indicates that the difference of the means is not significant at the 0.05 level.

ANOVAThreeWay (7/13/2023 12:59:31)

Descriptive Statistics

ANOVA

Means Comparisons

Tukey Test

Interactions 's Groupin

Days Post-Fracture	Gender	Genotype	Mean	Groups								
21 dpf	Male	WT	58.1602	A								
14 dpf	Female	WT	57.10103	A								
21 dpf	Female	WT	49.68965	A	B							
14 dpf	Male	WT	47.14433	A	B	C						
14 dpf	Female	PostnHet	45.82513	A	B	C	D					
21 dpf	Female	Spp1KO	43.89463	A	B	C	D					
21 dpf	Male	Spp1KO	43.0751	A	B	C	D					
21 dpf	Male	PostnHet	41.73287	A	B	C	D					
21 dpf	Female	PostnHet	41.31753		B	C	D					
14 dpf	Female	Spp1KO	37.19825		B	C	D	E				
21 dpf	Male	PostnKO	35.052		B	C	D	E	F			
14 dpf	Female	PostnKO	34.2609		B	C	D	E	F			
14 dpf	Male	Spp1KO	34.07427		B	C	D	E	F			
21 dpf	Female	PostnKO	33.09913			C	D	E	F			
14 dpf	Male	PostnHet	29.39753				D	E	F			
10 dpf	Male	WT	23.94287					E	F	G		
14 dpf	Male	PostnKO	23.32927					E	F	G	H	
10 dpf	Female	WT	18.4878						F	G	H	I
10 dpf	Female	Spp1KO	10.57603							G	H	I
10 dpf	Female	PostnHet	10.16607							G	H	I
10 dpf	Male	Spp1KO	7.907							G	H	I
10 dpf	Female	PostnKO	7.05433								H	I
10 dpf	Male	PostnHet	7.03007								H	I
10 dpf	Male	PostnKO	5.96547									I

Means that do not share a letter are significantly different.

Sig equals 1 indicates that the difference of the means is significant at the 0.05 level.
Sig equals 0 indicates that the difference of the means is not significant at the 0.05 level.

ANOVAThreeWay (7/13/2023 12:40:27) **Table S7. COX-2 IHC Percent COX-2+ Osteoclasts unit of measure = percent callus COX-2+ OCs/TRAP+ OCs**

Descriptive Statistics

Days Post-Fracture

	N	Mean	SD	SEM	Variance	Missing	NonMissing
10 dpf	30	65.5563	27.52965	5.29808	757.88154	3	27
14 dpf	41	66.73882	24.56609	4.21305	603.49258	7	34
21 dpf	33	69.17643	20.6866	3.9094	427.93536	5	28

Gender

	N	Mean	SD	SEM	Variance	Missing	NonMissing
Female	57	69.28833	23.3578	3.37141	545.58664	9	48
Male	47	64.64	25.1214	3.9233	631.08449	6	41

Genotype

	N	Mean	SD	SEM	Variance	Missing	NonMissing
WT	31	91.20783	8.85613	1.84663	78.43105	8	23
PostnHet	25	62.58476	17.48518	3.81558	305.73165	4	21
PostnKO	26	61.5704	23.14835	4.62967	535.84612	1	25
Spp1KO	22	51.238	24.1395	5.39776	582.71539	2	20

*Days Post-Fracture*Gender*

		N	Mean	SD	SEM	Variance	Missing	NonMissing
10 dpf	Female	15	62.51846	32.37427	8.97901	1048.09311	2	13
	Male	15	68.37714	23.03247	6.15569	530.49481	1	14
14 dpf	Female	23	69.52053	23.89963	5.48295	571.19247	4	19
	Male	18	63.21533	25.7745	6.65495	664.32474	3	15
21 dpf	Female	19	74.51313	10.80242	2.70061	116.69238	3	16
	Male	14	62.06083	28.18866	8.13736	794.6003	2	12

*Days Post-Fracture*Genotype*

		N	Mean	SD	SEM	Variance	Missing	NonMissing
10 dpf	WT	8	94.99429	5.40333	2.04227	29.19603	1	7
	PostnHet	7	60.65667	13.21578	5.39532	174.65691	1	6
	PostnKO	8	62.435	27.49798	9.72201	756.13911	0	8
	Spp1KO	7	40.27333	25.45573	10.39226	647.99431	1	6
14 dpf	WT	12	94.49889	5.3147	1.77157	28.24606	3	9
	PostnHet	11	59.45889	20.01247	6.67082	400.49911	2	9
	PostnKO	9	50.28375	24.20914	8.55922	586.08228	1	8
	Spp1KO	9	60.15375	18.73533	6.62394	351.01266	1	8
21 dpf	WT	11	83.19	10.61837	4.01337	112.7498	4	7
	PostnHet	7	69.20167	18.24505	7.44851	332.88198	1	6
	PostnKO	9	70.83444	14.43635	4.81212	208.4083	0	9
	Spp1KO	6	50.315	28.3272	11.56453	802.43047	0	6

ANOVAThreeWay (7/13/2023 12:40:27)

Descriptive Statistics

*Gender*Genotype*

		N	Mean	SD	SEM	Variance	Missing	NonMissing
Female	WT	15	90.65385	10.87588	3.01643	118.28481	2	13
	PostnHet	15	59.77455	13.26537	3.99966	175.97017	4	11
	PostnKO	15	65.77571	23.98448	6.41012	575.25532	1	14
	Spp1KO	12	56.896	26.55551	8.39759	705.195	2	10
Male	WT	16	91.928	5.74349	1.81625	32.98768	6	10
	PostnHet	10	65.676	21.53164	6.8089	463.61147	0	10
	PostnKO	11	56.21818	21.95289	6.61905	481.92938	0	11
	Spp1KO	10	45.58	21.30357	6.73678	453.842	0	10

*Days Post-Fracture*Gender*Genotype*

			N	Mean	SD	SEM	Variance	Missing	NonMissing
10 dpf	Female	WT	4	96.32	4.93354	2.46677	24.3398	0	4
		PostnHet	4	60.33	12.43312	7.17827	154.5825	1	3
		PostnKO	3	58.34	33.5868	19.39135	1128.0733	0	3
		Spp1KO	4	23.81667	20.10444	11.6073	404.18843	1	3
	Male	WT	4	93.22667	6.54803	3.7805	42.87663	1	3
		PostnHet	3	60.98333	16.7851	9.69088	281.73963	0	3
		PostnKO	5	64.892	27.1861	12.15799	739.08397	0	5
		Spp1KO	3	56.73	20.08312	11.59499	403.3317	0	3
14 dpf	Female	WT	5	97.782	1.34819	0.60293	1.81762	0	5
		PostnHet	7	53.888	6.99331	3.1275	48.90637	2	5
		PostnKO	6	56.632	28.44854	12.72258	809.31967	1	5
		Spp1KO	5	69.845	16.27743	8.13872	264.95477	1	4
	Male	WT	7	90.395	5.69901	2.84951	32.47877	3	4
		PostnHet	4	66.4225	29.77262	14.88631	886.40863	0	4
		PostnKO	3	39.70333	12.80547	7.39324	163.98013	0	3
		Spp1KO	4	50.4625	17.42471	8.71236	303.62056	0	4
21 dpf	Female	WT	6	76.0775	5.96585	2.98293	35.59142	2	4
		PostnHet	4	69.03	20.29456	11.71707	411.8691	1	3
		PostnKO	6	77.11333	11.22263	4.58162	125.94743	0	6
		Spp1KO	3	72.71	4.46278	2.57659	19.9164	0	3
	Male	WT	5	92.67333	6.98474	4.03264	48.78653	2	3
		PostnHet	3	69.37333	20.49994	11.83564	420.24743	0	3
		PostnKO	3	58.27667	12.80409	7.39244	163.94463	0	3
		Spp1KO	3	27.92	21.94429	12.66954	481.5517	0	3

ANOVAThreeWay (7/13/2023 12:40:27)

ANOVA

Overall ANOVA

	DF	Sum of Squares	Mean Square	F Value	P Value
Days Post-Fracture	2	172.08075	86.04037	0.28077	0.75611
Gender	1	245.1101	245.1101	0.79986	0.37443
Genotype	3	20079.50728	6693.16909	21.84169	6.75524E-10
Days Post-Fracture*Gender	2	1655.33219	827.6661	2.70091	0.07468
Days Post-Fracture*Genotype	6	3552.26036	592.04339	1.932	0.08877
Gender*Genotype	3	953.08301	317.69434	1.03673	0.38229
Days Post-Fracture*Gender*Genotype	6	4763.70682	793.95114	2.59089	0.02591
Model	23	31445.12814	1367.17948	4.46149	1.03031E-6
Error	65	19918.60574	306.44009		
Corrected Total	88	51363.73388			

At the 0.05 level, the population means of **Days Post-Fracture** are **not significantly** different.
At the 0.05 level, the population means of **Gender** are **not significantly** different.
At the 0.05 level, the population means of **Genotype** are **significantly** different.
At the 0.05 level, the population means of **Days Post-Fracture*Gender** are **not significantly** different.
At the 0.05 level, the population means of **Days Post-Fracture*Genotype** are **not significantly** different.
At the 0.05 level, the population means of **Gender*Genotype** are **not significantly** different.
At the 0.05 level, the population means of **Days Post-Fracture*Gender*Genotype** are **significantly** different.

ANOVAThreeWay (7/13/2023 12:40:27)

ANOVA

Means Comparisons

Tukey Test

Days Post-Fracture

	MeanDiff	SEM	q Value	Prob	Alpha	Sig	LCL	UCL
10 dpf 14 dpf	-1.31146	3.91513	-0.47372	0.94008	0.05	0	-10.70194	8.07902
10 dpf 21 dpf	-3.56694	4.12535	-1.22279	0.66436	0.05	0	-13.46161	6.32774
14 dpf 21 dpf	-2.25548	3.90022	-0.81783	0.83216	0.05	0	-11.61019	7.09923

Gender

	MeanDiff	SEM	q Value	Prob	Alpha	Sig	LCL	UCL
Female Male	3.40215	3.25093	1.48	0.2992	0.05	0	-3.09041	9.89471

Genotype

	MeanDiff	SEM	q Value	Prob	Alpha	Sig	LCL	UCL
WT PostnHet	27.74122	4.59751	8.53332	4.88415E-7	0.05	1	15.61869	39.86375
WT PostnKO	31.91953	4.44861	10.14722	0	0.05	1	20.18961	43.64944
WT Spp1KO	40.83172	4.63119	12.46866	0	0.05	1	28.62038	53.04306
PostnHet PostnKO	4.17831	4.56358	1.29482	0.79662	0.05	0	-7.85476	16.21137
PostnHet Spp1KO	13.0905	4.74174	3.90422	0.03669	0.05	1	0.58768	25.59332
PostnKO Spp1KO	8.91219	4.59751	2.74143	0.22235	0.05	0	-3.21034	21.03473

Days Post-Fracture 's

	Mean	Groups
21 dpf	69.17643	A
14 dpf	66.73882	A
10 dpf	65.5563	A

Means that do not share a letter are significantly different.

Gender 's Grouping Le

	Mean	Groups
Female	69.28833	A
Male	64.64	A

Means that do not share a letter are significantly different.

Sig equals 1 indicates that the difference of the means is significant at the 0.05 level.
Sig equals 0 indicates that the difference of the means is not significant at the 0.05 level.

ANOVAThreeWay (7/13/2023 12:40:27)

ANOVA

Means Comparisons

Tukey Test

Genotype 's Grouping

	Mean	Groups		
WT	91.20783	A		
PostnHet	62.58476		B	
PostnKO	61.5704		B	C
Spp1KO	51.238			C

Means that do not share a letter are significantly different.

Interactions 's Groupin

Days Post-Fracture	Gender	Genotype	Mean	Groups				
14 dpf	Female	WT	97.782	A				
10 dpf	Female	WT	96.32	A				
10 dpf	Male	WT	93.22667	A	B			
21 dpf	Male	WT	92.67333	A	B			
14 dpf	Male	WT	90.395	A	B			
21 dpf	Female	PostnKO	77.11333	A	B	C		
21 dpf	Female	WT	76.0775	A	B	C		
21 dpf	Female	Spp1KO	72.71	A	B	C	D	
14 dpf	Female	Spp1KO	69.845	A	B	C	D	
21 dpf	Male	PostnHet	69.37333	A	B	C	D	E
21 dpf	Female	PostnHet	69.03	A	B	C	D	E
14 dpf	Male	PostnHet	66.4225	A	B	C	D	E
10 dpf	Male	PostnKO	64.892	A	B	C	D	E
10 dpf	Male	PostnHet	60.98333	A	B	C	D	E
10 dpf	Female	PostnHet	60.33	A	B	C	D	E
10 dpf	Female	PostnKO	58.34	A	B	C	D	E
21 dpf	Male	PostnKO	58.27667	A	B	C	D	E
10 dpf	Male	Spp1KO	56.73	A	B	C	D	E
14 dpf	Female	PostnKO	56.632		B	C	D	E
14 dpf	Female	PostnHet	53.888		B	C	D	E
14 dpf	Male	Spp1KO	50.4625		B	C	D	E
14 dpf	Male	PostnKO	39.70333			C	D	E
21 dpf	Male	Spp1KO	27.92				D	E
10 dpf	Female	Spp1KO	23.81667					E

Means that do not share a letter are significantly different.

Sig equals 1 indicates that the difference of the means is significant at the 0.05 level.
Sig equals 0 indicates that the difference of the means is not significant at the 0.05 level.

ANOVATwoWay (7/31/2023 14:24:01)

Table S8-A. Relative Aggrecan mRNA Levels

Descriptive Statistics

Gender

	N	Mean	SD	SEM	Variance	Missing	NonMissing
Female	10	1.2156	1.10843	0.35052	1.22861	0	10
Male	9	1.87117	0.87199	0.29066	0.76037	0	9

Genotype

	N	Mean	SD	SEM	Variance	Missing	NonMissing
WT	7	1.39853	0.82757	0.31279	0.68488	0	7
PostnKO	6	2.34373	1.02165	0.41709	1.04378	0	6
Spp1KO	6	0.8574	0.77975	0.31833	0.60802	0	6

Overall

	N	Mean	SD	SEM	Variance	Missing	NonMissing
	19	1.52613	1.03216	0.23679	1.06535	0	19

ANOVATwoWay (7/31/2023 14:24:01)

ANOVA

Overall ANOVA

	DF	Sum of Squares	Mean Square	F Value	P Value
Gender	1	1.96602	1.96602	2.57367	0.13266
Genotype	2	6.73755	3.36877	4.40999	0.03452
Interaction	2	0.47526	0.23763	0.31108	0.73796
Model	5	9.24557	1.84911	2.42064	0.09247
Error	13	9.93065	0.7639		
Corrected Total	18	19.17622			

At the 0.05 level, the population means of **Gender** are **not significantly** different.
At the 0.05 level, the population means of **Genotype** are **significantly** different.
At the 0.05 level, the interaction between **Gender** and **Genotype** is **not significant**.

Means Comparisons

Tukey Test

Gender

	MeanDiff	SEM	q Value	Prob	Alpha	Sig	LCL	UCL
Male Female	0.65557	0.40158	2.30867	0.12655	0.05	0	-0.21199	1.52313

Gender 's Grouping Letters Table

	Mean	Groups
Male	1.87117	A
Female	1.2156	A

Means that do not share a letter are significantly different.

Genotype 's Grouping Letters Table

	Mean	Groups	
PostnKO	2.34373	A	
WT	1.39853	A	B
Spp1KO	0.8574		B

Means that do not share a letter are significantly different.

Interactions 's Grouping Letters Table

Gender	Genotype	Mean	Groups
Male	PostnKO	2.47952	A
Female	PostnKO	2.20794	A
Male	WT	1.74455	A
Male	Spp1KO	1.38944	A
Female	WT	1.13902	A
Female	Spp1KO	0.32537	A

Means that do not share a letter are significantly different.

Sig equals 1 indicates that the difference of the means is significant at the 0.05 level.
Sig equals 0 indicates that the difference of the means is not significant at the 0.05 level.

ANOVATwoWay (7/31/2023 14:14:11) **Table S8-B. Relative MMP-13 mRNA Levels**

Descriptive Statistics

Gender

	N	Mean	SD	SEM	Variance	Missing	NonMissing
Female	10	0.42725	0.61183	0.19348	0.37433	0	10
Male	9	0.60869	0.95152	0.31717	0.9054	0	9

Genotype

	N	Mean	SD	SEM	Variance	Missing	NonMissing
WT	7	1.2939	0.81233	0.30703	0.65988	0	7
PostnKO	6	0.09958	0.07974	0.03255	0.00636	0	6
Spp1KO	6	0.01599	0.01267	0.00517	1.60528E-4	0	6

Overall

	N	Mean	SD	SEM	Variance	Missing	NonMissing
	19	0.51319	0.77345	0.17744	0.59823	0	19

ANOVA

Overall ANOVA

	DF	Sum of Squares	Mean Square	F Value	P Value
Gender	1	0.28506	0.28506	1.14314	0.30444
Genotype	2	7.20553	3.60277	14.44784	4.972E-4
Interaction	2	0.4188	0.2094	0.83974	0.45395
Model	5	7.5264	1.50528	6.03649	0.00421
Error	13	3.24173	0.24936		
Corrected Total	18	10.76813			

At the 0.05 level, the population means of **Gender** are **not significantly** different.
At the 0.05 level, the population means of **Genotype** are **significantly** different.
At the 0.05 level, the interaction between **Gender** and **Genotype** is **not significant**.

ANOVATwoWay (7/31/2023 14:14:11)

ANOVA

Means Comparisons

Tukey Test

Gender

	MeanDiff	SEM	q Value	Prob	Alpha	Sig	LCL	UCL
Male Female	0.18145	0.22944	1.11839	0.44324	0.05	0	-0.31423	0.67713

Genotype

	MeanDiff	SEM	q Value	Prob	Alpha	Sig	LCL	UCL
PostnKO WT	-1.19432	0.27782	6.07954	0.00232	0.05	1	-1.92788	-0.46075
Spp1KO WT	-1.27791	0.27782	6.50508	0.00134	0.05	1	-2.01148	-0.54435
Spp1KO PostnKO	-0.0836	0.28831	0.41006	0.95487	0.05	0	-0.84485	0.67766

Gender 's Grouping Letters Table

	Mean	Groups
Male	0.60869	A
Female	0.42725	A

Means that do not share a letter are significantly different.

Genotype 's Grouping Letters Table

	Mean	Groups	
WT	1.2939	A	
PostnKO	0.09958		B
Spp1KO	0.01599		B

Means that do not share a letter are significantly different.

Interactions 's Grouping Letters Table

Gender	Genotype	Mean	Groups	
Male	WT	1.67015	A	
Female	WT	1.01171	A	B
Male	PostnKO	0.13289		B
Female	PostnKO	0.06627		B
Male	Spp1KO	0.02304		B
Female	Spp1KO	0.00893		B

Means that do not share a letter are significantly different.

Sig equals 1 indicates that the difference of the means is significant at the 0.05 level.
Sig equals 0 indicates that the difference of the means is not significant at the 0.05 level.

ANOVATwoWay (7/31/2023 14:16:16) **Table S8-C. Relative CD31 (Pecam1) mRNA Levels**

Descriptive Statistics

Gender

	N	Mean	SD	SEM	Variance	Missing	NonMissing
Female	10	1.00039	0.95841	0.30308	0.91855	0	10
Male	9	0.58936	0.69562	0.23187	0.48389	0	9

Genotype

	N	Mean	SD	SEM	Variance	Missing	NonMissing
WT	7	1.43557	1.05581	0.39906	1.11474	0	7
PostnKO	6	0.57796	0.53325	0.2177	0.28435	0	6
Spp1KO	6	0.29856	0.19831	0.08096	0.03933	0	6

Overall

	N	Mean	SD	SEM	Variance	Missing	NonMissing
	19	0.80569	0.84782	0.1945	0.71879	0	19

ANOVA

Overall ANOVA

	DF	Sum of Squares	Mean Square	F Value	P Value
Gender	1	0.50282	0.50282	0.90985	0.35755
Genotype	2	4.12406	2.06203	3.73126	0.05241
Interaction	2	0.55757	0.27878	0.50446	0.61518
Model	5	5.75403	1.15081	2.08239	0.13303
Error	13	7.18428	0.55264		
Corrected Total	18	12.93831			

At the 0.05 level, the population means of **Gender** are **not significantly** different.
At the 0.05 level, the population means of **Genotype** are **not significantly** different.
At the 0.05 level, the interaction between **Gender** and **Genotype** is **not significant**.

ANOVATwoWay (7/31/2023 14:16:16)

ANOVA

Means Comparisons

Tukey Test

Gender

	MeanDiff	SEM	q Value	Prob	Alpha	Sig	LCL	UCL
Male Female	-0.41102	0.34157	1.70179	0.2503	0.05	0	-1.14893	0.32689

Genotype

	MeanDiff	SEM	q Value	Prob	Alpha	Sig	LCL	UCL
PostnKO WT	-0.85761	0.41359	2.93251	0.1342	0.05	0	-1.94966	0.23444
Spp1KO WT	-1.13701	0.41359	3.88787	0.04104	0.05	1	-2.22906	-0.04496
Spp1KO PostnKO	-0.2794	0.4292	0.92061	0.79508	0.05	0	-1.41267	0.85388

Gender 's Grouping Letters Table

	Mean	Groups
Female	1.00039	A
Male	0.58936	A

Means that do not share a letter are significantly different.

Genotype 's Grouping Letters Table

	Mean	Groups	
WT	1.43557	A	
PostnKO	0.57796	A	B
Spp1KO	0.29856		B

Means that do not share a letter are significantly different.

Interactions 's Grouping Letters Table

Gender	Genotype	Mean	Groups
Female	WT	1.7545	A
Male	WT	1.01033	A
Male	PostnKO	0.62301	A
Female	PostnKO	0.5329	A
Female	Spp1KO	0.46238	A
Male	Spp1KO	0.13474	A

Means that do not share a letter are significantly different.

Sig equals 1 indicates that the difference of the means is significant at the 0.05 level.
Sig equals 0 indicates that the difference of the means is not significant at the 0.05 level.

ANOVATwoWay (7/31/2023 14:22:15) **Table S8-D. Relative Ctsk mRNA Levels**

Descriptive Statistics

Gender

	N	Mean	SD	SEM	Variance	Missing	NonMissing
Female	10	0.88761	0.37302	0.12434	0.13915	1	9
Male	9	0.93633	0.50651	0.16884	0.25655	0	9

Genotype

	N	Mean	SD	SEM	Variance	Missing	NonMissing
WT	7	1.1134	0.5299	0.21633	0.28079	1	6
PostnKO	6	0.97906	0.31362	0.12803	0.09836	0	6
Spp1KO	6	0.64345	0.33984	0.13874	0.11549	0	6

Overall

	N	Mean	SD	SEM	Variance	Missing	NonMissing
	19	0.91197	0.43225	0.10188	0.18684	1	18

ANOVA

Overall ANOVA

	DF	Sum of Squares	Mean Square	F Value	P Value
Gender	1	0.01068	0.01068	0.0632	0.80576
Genotype	2	0.70307	0.35154	2.08017	0.16764
Interaction	2	0.43458	0.21729	1.28578	0.31193
Model	5	1.14833	0.22967	1.35902	0.30592
Error	12	2.02792	0.16899		
Corrected Total	17	3.17625			

At the 0.05 level, the population means of **Gender** are **not significantly** different.
At the 0.05 level, the population means of **Genotype** are **not significantly** different.
At the 0.05 level, the interaction between **Gender** and **Genotype** is **not significant**.

ANOVATwoWay (7/31/2023 14:22:15)

ANOVA

Means Comparisons

Tukey Test

Gender

	MeanDiff	SEM	q Value	Prob	Alpha	Sig	LCL	UCL
Male Female	0.04872	0.19379	0.35553	0.80576	0.05	0	-0.37351	0.47095

Genotype

	MeanDiff	SEM	q Value	Prob	Alpha	Sig	LCL	UCL
PostnKO WT	-0.13434	0.23734	0.80047	0.84034	0.05	0	-0.76753	0.49886
Spp1KO WT	-0.46995	0.23734	2.80022	0.15957	0.05	0	-1.10315	0.16324
Spp1KO PostnKO	-0.33561	0.23734	1.99976	0.36485	0.05	0	-0.96881	0.29758

Gender 's Grouping Letters Table

	Mean	Groups
Male	0.93633	A
Female	0.88761	A

Means that do not share a letter are significantly different.

Genotype 's Grouping Letters Table

	Mean	Groups
WT	1.1134	A
PostnKO	0.97906	A
Spp1KO	0.64345	A

Means that do not share a letter are significantly different.

Interactions 's Grouping Letters Table

Gender	Genotype	Mean	Groups
Male	WT	1.34482	A
Female	PostnKO	1.12195	A
Female	WT	0.88199	A
Male	PostnKO	0.83617	A
Female	Spp1KO	0.6589	A
Male	Spp1KO	0.628	A

Means that do not share a letter are significantly different.

Sig equals 1 indicates that the difference of the means is significant at the 0.05 level.
Sig equals 0 indicates that the difference of the means is not significant at the 0.05 level.

ANOVATwoWay (7/31/2023 14:20:33) **Table S8-E. Relative TRAP (Acp5) mRNA Levels**

Descriptive Statistics

Gender

	N	Mean	SD	SEM	Variance	Missing	NonMissing
Female	10	0.7905	0.74372	0.23519	0.55312	0	10
Male	9	0.69266	0.79182	0.26394	0.62697	0	9

Genotype

	N	Mean	SD	SEM	Variance	Missing	NonMissing
WT	7	1.32254	0.93795	0.35451	0.87974	0	7
PostnKO	6	0.6641	0.21587	0.08813	0.0466	0	6
Spp1KO	6	0.14942	0.07113	0.02904	0.00506	0	6

Overall

	N	Mean	SD	SEM	Variance	Missing	NonMissing
	19	0.74415	0.74682	0.17133	0.55773	0	19

ANOVA

Overall ANOVA

	DF	Sum of Squares	Mean Square	F Value	P Value
Gender	1	0.00741	0.00741	0.01754	0.89665
Genotype	2	4.46655	2.23328	5.28427	0.02092
Interaction	2	0.03617	0.01808	0.04279	0.95825
Model	5	4.54505	0.90901	2.15086	0.12345
Error	13	5.49415	0.42263		
Corrected Total	18	10.0392			

At the 0.05 level, the population means of **Gender** are **not significantly** different.
At the 0.05 level, the population means of **Genotype** are **significantly** different.
At the 0.05 level, the interaction between **Gender** and **Genotype** is **not significant**.

ANOVATwoWay (7/31/2023 14:20:33)

ANOVA

Means Comparisons

Tukey Test

Gender

	MeanDiff	SEM	q Value	Prob	Alpha	Sig	LCL	UCL
Male Female	-0.09783	0.2987	0.46319	0.74849	0.05	0	-0.74313	0.54747

Genotype

	MeanDiff	SEM	q Value	Prob	Alpha	Sig	LCL	UCL
PostnKO WT	-0.65844	0.36168	2.57458	0.20157	0.05	0	-1.61343	0.29655
Spp1KO WT	-1.17312	0.36168	4.58705	0.01644	0.05	1	-2.12812	-0.21813
Spp1KO PostnKO	-0.51468	0.37533	1.93926	0.38349	0.05	0	-1.50573	0.47636

Gender 's Grouping Letters Table

	Mean	Groups
Female	0.7905	A
Male	0.69266	A

Means that do not share a letter are significantly different.

Genotype 's Grouping Letters Table

	Mean	Groups	
WT	1.32254	A	
PostnKO	0.6641	A	B
Spp1KO	0.14942		B

Means that do not share a letter are significantly different.

Interactions 's Grouping Letters Table

Gender	Genotype	Mean	Groups
Male	WT	1.33481	A
Female	WT	1.31335	A
Female	PostnKO	0.74668	A
Male	PostnKO	0.58152	A
Male	Spp1KO	0.16166	A
Female	Spp1KO	0.13717	A

Means that do not share a letter are significantly different.

Sig equals 1 indicates that the difference of the means is significant at the 0.05 level.
Sig equals 0 indicates that the difference of the means is not significant at the 0.05 level.

ANOVATwoWay (7/31/2023 14:09:23) **Table S8-F. Relative COX-2 (Ptgs2) mRNA Levels**

Descriptive Statistics

Gender

	N	Mean	SD	SEM	Variance	Missing	NonMissing
Female	10	0.52079	0.73338	0.23191	0.53784	0	10
Male	9	0.57401	0.68809	0.22936	0.47347	0	9

Genotype

	N	Mean	SD	SEM	Variance	Missing	NonMissing
WT	7	1.20939	0.67437	0.25489	0.45477	0	7
PostnKO	6	0.28617	0.40944	0.16715	0.16764	0	6
Spp1KO	6	0.03187	0.02554	0.01043	6.52091E-4	0	6

Overall

	N	Mean	SD	SEM	Variance	Missing	NonMissing
	19	0.546	0.69289	0.15896	0.4801	0	19

ANOVA

Overall ANOVA

	DF	Sum of Squares	Mean Square	F Value	P Value
Gender	1	0.0728	0.0728	0.27728	0.60736
Genotype	2	5.08015	2.54008	9.67406	0.00267
Interaction	2	0.08447	0.04224	0.16086	0.85308
Model	5	5.22839	1.04568	3.98254	0.02067
Error	13	3.41335	0.26257		
Corrected Total	18	8.64175			

At the 0.05 level, the population means of **Gender** are **not significantly** different.
At the 0.05 level, the population means of **Genotype** are **significantly** different.
At the 0.05 level, the interaction between **Gender** and **Genotype** is **not significant**.

ANOVATwoWay (7/31/2023 14:09:23)

ANOVA

Means Comparisons

Tukey Test

Gender

	MeanDiff	SEM	q Value	Prob	Alpha	Sig	LCL	UCL
Male Female	0.05321	0.23544	0.31964	0.8247	0.05	0	-0.45542	0.56184

Genotype

	MeanDiff	SEM	q Value	Prob	Alpha	Sig	LCL	UCL
PostnKO WT	-0.92322	0.28508	4.57989	0.0166	0.05	1	-1.67595	-0.17049
Spp1KO WT	-1.17752	0.28508	5.84139	0.00316	0.05	1	-1.93025	-0.42478
Spp1KO PostnKO	-0.25429	0.29584	1.21561	0.67405	0.05	0	-1.03544	0.52685

Gender 's Grouping Letters Table

	Mean	Groups
Male	0.57401	A
Female	0.52079	A

Means that do not share a letter are significantly different.

Genotype 's Grouping Letters Table

	Mean	Groups	
WT	1.20939	A	
PostnKO	0.28617		B
Spp1KO	0.03187		B

Means that do not share a letter are significantly different.

Interactions 's Grouping Letters Table

Gender	Genotype	Mean	Groups
Male	WT	1.27187	A
Female	WT	1.16253	A
Male	PostnKO	0.43584	A
Female	PostnKO	0.1365	A
Female	Spp1KO	0.04944	A
Male	Spp1KO	0.01431	A

Means that do not share a letter are significantly different.

Sig equals 1 indicates that the difference of the means is significant at the 0.05 level.
Sig equals 0 indicates that the difference of the means is not significant at the 0.05 level.
